# Glucose-dependent insulinotropic polypeptide (GIP)

**DOI:** 10.1016/j.molmet.2025.102118

**Published:** 2025-02-28

**Authors:** Timo D. Müller, Alice Adriaenssens, Bo Ahrén, Matthias Blüher, Andreas L. Birkenfeld, Jonathan E. Campbell, Matthew P. Coghlan, David D'Alessio, Carolyn F. Deacon, Stefano DelPrato, Jonathan D. Douros, Daniel J. Drucker, Natalie S. Figueredo Burgos, Peter R. Flatt, Brian Finan, Ruth E. Gimeno, Fiona M. Gribble, Matthew R. Hayes, Christian Hölscher, Jens J. Holst, Patrick J. Knerr, Filip K. Knop, Christine M. Kusminski, Arkadiusz Liskiewicz, Guillaume Mabilleau, Stephanie A. Mowery, Michael A. Nauck, Aaron Novikoff, Frank Reimann, Anna G. Roberts, Mette M. Rosenkilde, Ricardo J. Samms, Philip E. Scherer, Randy J. Seeley, Kyle W. Sloop, Christian Wolfrum, Denise Wootten, Richard D. DiMarchi, Matthias H. Tschöp

**Affiliations:** 1 Institute for Diabetes and Obesity, Helmholtz Munich, Germany; 2 German Center for Diabetes Research, DZD, Germany; 3 Walther-Straub Institute for Pharmacology and Toxicology, Ludwig-Maximilians-University Munich (LMU), Germany; 4 Centre for Cardiovascular and Metabolic Neuroscience, Department of Neuroscience, Physiology, and Pharmacology, University College London, London, UK; 5 Department of Clinical Sciences, Lund, Lund University, Lund, Sweden; 6 Medical Department III-Endocrinology, Nephrology, Rheumatology, University of Leipzig Medical Center, Leipzig, Germany; 7 Helmholtz Institute for Metabolic, Obesity and Vascular Research (HI-MAG) of the Helmholtz Zentrum München at the University of Leipzig and University Hospital Leipzig, Leipzig, Germany; 8 Department of Internal Medicine IV, University Hospital Tübingen, Tübingen 72076, Germany; 9 Institute of Diabetes Research and Metabolic Diseases of the Helmholtz Centre Munich, Tübingen, Germany; 10 German Center for Diabetes Research, Neuherberg, Germany; 11 Duke Molecular Physiology Institute, Duke University, Durham, NC, USA; 12 Department of Medicine, Division of Endocrinology, Duke University, Durham, NC, USA; 13 Department of Pharmacology and Cancer Biology, Duke University, Durham, NC, USA; 14 Lilly Research Laboratories, Eli Lilly and Company, Indianapolis, IN 46285, USA; 15 School of Biomedical Sciences, Ulster University, Coleraine, UK; 16 Department of Biomedical Sciences, University of Copenhagen, Copenhagen, Denmark; 17 Interdisciplinary Research Center “Health Science”, Sant'Anna School of Advanced Studies, Pisa, Italy; 18 Indianapolis Biosciences Research Institute, Indianapolis, IN, USA; 19 The Lunenfeld-Tanenbaum Research Institute, Mt. Sinai Hospital, and the Department of Medicine, University of Toronto, Toronto, Ontario, Canada; 20 Diabetes Research Centre, School of Biomedical Sciences, Ulster University, Coleraine, Northern Ireland BT52 1SA, UK; 21 Institute of Metabolic Science-Metabolic Research Laboratories & MRC-Metabolic Diseases Unit, University of Cambridge, Cambridge, UK; 22 Department of Biobehavioral Health Sciences, School of Nursing, University of Pennsylvania, Philadelphia, PA, USA; 23 Department of Psychiatry, Perelman School of Medicine, University of Pennsylvania, Philadelphia, PA, USA; 24 Neurodegeneration Research Group, Henan Academy of Innovations in Medical Science, Xinzheng, China; 25 Department of Biomedical Sciences and the Novo Nordisk Foundation Centre for Basic Metabolic Research, University of Copenhagen, Copenhagen, Denmark; 26 Center for Clinical Metabolic Research, Herlev and Gentofte Hospital, University of Copenhagen, Hellerup, Denmark; 27 Clinical Research, Steno Diabetes Center Copenhagen, Herlev, Denmark; 28 Department of Clinical Medicine, Faculty of Health and Medical Sciences, University of Copenhagen, Copenhagen, Denmark; 29 Touchstone Diabetes Center, University of Texas Southwestern Medical Center, Dallas, TX, USA; 30 Department of Physiology, Faculty of Medical Sciences in Katowice, Medical University of Silesia, Katowice, Poland; 31 Univ Angers, Nantes Université, ONIRIS, Inserm, RMeS UMR 1229, Angers, France; 32 CHU Angers, Departement de Pathologie Cellulaire et Tissulaire, Angers, France; 33 Diabetes, Endocrinology and Metabolism Section, Department of Internal Medicine I, St. Josef-Hospital, Ruhr-University Bochum, Bochum, Germany; 34 Department of Biomedical Sciences, Faculty of Health and Medical Sciences University of Copenhagen, Copenhagen, Denmark; 35 Department of Surgery, University of Michigan, Ann Arbor, MI, USA; 36 Institute of Food, Nutrition and Health, ETH Zurich, 8092, Schwerzenbach, Switzerland; 37 Drug Discovery Biology, Monash Institute of Pharmaceutical Sciences, Monash University, Parkville, VIC, Australia; 38 ARC Centre for Cryo-electron Microscopy of Membrane Proteins, Monash Institute of Pharmaceutical Sciences, Monash University, Parkville, VIC, Australia; 39 Department of Chemistry, Indiana University, Bloomington, IN, USA; 40 Helmholtz Munich, Neuherberg, Germany; 41 Division of Metabolic Diseases, Department of Medicine, Technical University of Munich, Munich, Germany

**Keywords:** Diabetes, GLP-1, GIP, Incretin, Insulin, Obesity

## Abstract

**Background:**

Glucose-dependent insulinotropic polypeptide (GIP) was the first incretin identified and plays an essential role in the maintenance of glucose tolerance in healthy humans. Until recently GIP had not been developed as a therapeutic and thus has been overshadowed by the other incretin, glucagon-like peptide 1 (GLP-1), which is the basis for several successful drugs to treat diabetes and obesity. However, there has been a rekindling of interest in GIP biology in recent years, in great part due to pharmacology demonstrating that both GIPR agonism and antagonism may be beneficial in treating obesity and diabetes. This apparent paradox has reinvigorated the field, led to new lines of investigation, and deeper understanding of GIP.

**Scope of Review:**

In this review, we provide a detailed overview on the multifaceted nature of GIP biology and discuss the therapeutic implications of GIPR signal modification on various diseases.

**Major Conclusions:**

Following its classification as an incretin hormone, GIP has emerged as a pleiotropic hormone with a variety of metabolic effects outside the endocrine pancreas. The numerous beneficial effects of GIPR signal modification render the peptide an interesting candidate for the development of pharmacotherapies to treat obesity, diabetes, drug-induced nausea and both bone and neurodegenerative disorders.

## 1 Identification of GIP

At the turn of the 19th century, Ivan Pavlov [1] and others [2,3] established that the secretion of pancreatic juice is induced upon entry of acidic chyme into the duodenum, and that this pancreatic secretion is accelerated by infusion of hydrochloric acid (HCL) into the stomach. Pavlov hypothesized that secretion of pancreatic juice is induced via a neuronal reflex [1]; however, pancreatic secretion prevailed in dogs following denervation of the intestinal vagal and splanchnic nerves [2,3], indicating that pancreatic secretion must be mediated by another, as yet unknown, mechanism. Then in 1902, William Bayliss and Ernest Starling discovered a mucosal substance they named secretin, which is released from the duodenal epithelium upon contact with acidic chyme, and is transported via the blood to the pancreas to stimulate the secretion of pancreatic juice [4]. By their demonstration that the digestive organs regulate nutrient homeostasis through exchange of blood-borne factors, Bayliss and Starling, who proposed the name hormone for such a substance (from Greek *hormōn* meaning “set in motion”) set the stage for a series of seminal discoveries that, using crude tissue extracts and impure hormone preparations, established the current view of the gut as an endocrine organ.

In 1905, soon after the identification of secretin, administration of gastrointestinal mucosal extracts in dogs led to the discovery of an endocrine factor regulating gastric acid secretion with the proposed name gastrin [5,6]. This was followed in 1928 by the discovery of an endocrine factor regulating gall bladder motility, cholecystokinin (CCK) [7]. By using crude tissue homogenates it was also shown that the pancreas produces hormone-like substances that decrease [8,9] or elevate [10,11] blood glucose levels, which subsequently led to the identification of insulin in 1921 [12] and of glucagon in 1923 [13]. A series of studies in the early 20^'th^ century further pointed to the existence of hormonal signals in the small and large intestines that inhibit gut motility and gastric acid secretion in response to ingestion of fat. Consistent with this was the observation that gastric emptying is delayed upon ingestion of a fatty meal [14,15], that lipid-induced inhibition of gastric motility depends on the presence of fat in the duodenum rather than in the stomach itself [16], and that intraduodenal administration of olive oil inhibits meal-stimulated gastric acid secretion [17,18]. The observation that dogs with completely denervated gastric pouches retained the inhibition of gastric secretion induced by ingestion of fat led Takashi Kosaka and Robert Kho-Seng Lim in 1930 to hypothesize that fat-mediated inhibition of gastric secretion is mediated by another unidentified intestinal hormone, which they named enterogastrone (i.e. a gut-derived hormone that decreases gastric secretion and motility) [18,19]. Kosaka and Lim further observed that dog intestinal extracts inhibit gastric secretion, and since CCK inhibited gastric secretion only at very large doses, they hypothesized that a preserved enterogastrone in the preparation gave rise to this inhibitory effect [19]. Despite intense efforts, robust evidence for the enterogastrone remained elusive. In 1970, John Brown and Raymond Pederson compared the gastric inhibitory effect of two crude, differentially concentrated, porcine duodenal and jejunal CCK preparations [20]. When given to dogs with vagally and sympathetically denervated stomach pouches, inhibition of gastric acid secretion was greater with the less-concentrated CCK preparation as opposed to the higher one, suggesting that the cruder preparation contained an unknown gastric inhibitory factor that was removed during further CCK purification [20]. In agreement with this, only the less-pure preparation inhibited gastrin-induced gastric acid secretion [21]. In 1969/70, and with the help of Viktor Mutt and Erik Jorpes, who had already succeeded in purifying and characterizing secretin [22] and CCK [23], Pederson and Brown purified a substance from porcine intestinal extracts that showed potent inhibitory action on gut motility and gastric acid secretion, but without the classical effects of CCK to induce gallbladder contraction and pancreatic enzyme secretion, nor of secretin to induce bicarbonate secretion [24,25]. Based on the observation that the substance inhibited gastric motility and gastric acid secretion, albeit only at high doses, the substance was named “gastric inhibitory polypeptide (GIP)” [26].

## 2 Identification of GIP as an incretin hormone

Evidence pointing to the intestine as a source of glucoregulatory hormones dates back to the work of Benjamin Moore, who in 1906 reported that repeated oral administration of an intestinal mucosal extract decreased glucosuria in people living with diabetes [27]. Following the concept of hormonal signaling established by Bayliss and Starling [4], Moore hypothesized that this effect was mediated by an intestinal hormone that signals to the pancreas to lower blood glucose [27]. In 1929, Edgard Zunz and Jean LaBarre showed that intravenous (i.v.) infusion of intestinal mucosal extracts lowered blood glucose in rabbits and dogs [28]. Hypothesizing that the glucose-lowering effect results from an intestinal hormone that stimulates insulin secretion, the factor was named ‘incretin’ (stimulating endocrine pancreatic secretion) [28,29], as opposed to ‘excretin’ (stimulating exocrine pancreatic secretion).

Although not undisputed [[30], [31], [32], [33], [34]], multiple lines of evidence emerged over the next decades, which supported the overall notion that the intestine exerts hormonal control over glucose metabolism, which Roger Unger first referred to as the entero–insular axis [35,36]. Among the numerous studies that linked the intestine to glucose metabolism was the demonstration of enhanced blood glucose disposal in rodents when glucose is given orally relative to i.v. infusions [37]. Following the development of the first insulin radioimmunoassay (RIA) by Rosalyn Yalow and Solomon Berson in 1959/60 [38,39], studies in dogs [40] and humans [41,42] revealed that plasma insulin concentrations were greatly enhanced when glucose passes through the gut relative to parenteral administration, thereby supporting the existence of the ‘incretin effect’, ascribed to the insulinotropic action of gut-derived incretin hormones.

**Figure 1:**
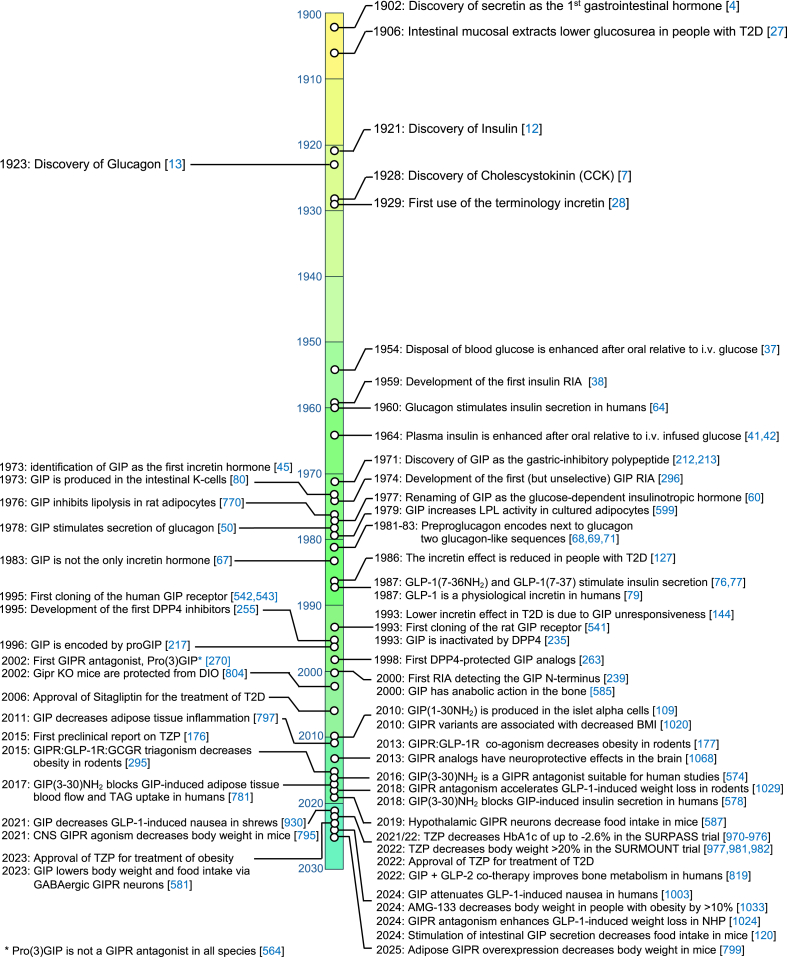
Timeline highlighting major achievements in glucose and incretin hormone metabolism.

In 1964/65, using a mucosal-derived CCK preparation of comparable impurity as the one used by Brown and Pederson [20,21], John Dupre showed that i.v. co-infusion of glucose with this preparation accelerated glucose disposal in humans [43,44], and that this was accompanied by elevated serum levels of immunoreactive (IR) insulin [44]. The extract did not affect glucose tolerance or insulin levels in subjects with juvenile (type 1) diabetes [44], suggesting that the extract harbored an unknown hormone with insulinotropic actions. Soon after Brown, Mutt and Pederson purified GIP from mucosal CCK extracts [24,25], Dupre hypothesized that GIP might also be responsible for the insulinotropic effect of his mucosal preparation. Dupre and Brown then jointly demonstrated in healthy humans that i.v. infusion of GIP together with glucose increases serum IR insulin and improves glucose tolerance, hence identifying GIP as the first incretin hormone [45] (Figure 1). Studies in isolated rat islets [[46], [47], [48], [49]], and in the perfused pancreas of dogs [50] and humans [51] then confirmed that GIP acts directly on the pancreas to enhance glucose-stimulated insulin secretion, followed by the demonstration in humans that GIP augments insulin secretion and reduces levels of blood glucose after meal ingestion [52]. While GIP, at very high doses, seemingly fulfills the criterion of being an enterogastrone, its physiological role to regulate gastric function in humans has been questioned [21,[53], [54], [55], [56]]. Dedicated studies showed that GIP affects neither gastric emptying [57] nor gastric acid secretion [54] when administered to mimic the physiological post-prandial concentrations in humans, and GIP did now show no meaningful inhibitory effects on gastric acid secretion induced by pentagastrin, whether at near-physiological doses [58], or at supraphysiological concentrations [54]. Since the insulinotropic action of GIP prevails at physiological concentrations in humans [26,45] and in the isolated perfused rat pancreas [59], this led to the redefinition of the acronym GIP as the glucose-dependent insulinotropic polypeptide [60] (Figure 1). Personal reflections on the discovery and early research on GIP have been chronicled in detail by Brown and his associates elsewhere [[61], [62], [63]]. In summary, although GIP was discovered as a gut-derived hormone that stimulated gastric acid secretion, this effect was, in contrast to its insulinotropic action, not observed under physiological conditions. This led to its redefinition as the glucose-dependent insulinotropic polypeptide.

## 3 Identification of GLP-1 as the second incretin hormone

It was already reported in 1960 [64], and later confirmed by other studies [65], that i.v. infusion of glucagon increases plasma insulin levels in humans. The glucagon-induced rise in plasma insulin peaked around 4 min after i.v. infusion, and was not related to glucagon's ability to increase blood glucose [65]. In 1980, Kjeld Lauritzen observed that individuals with ileal resection exhibited a diminished incretin effect despite normal elevation of plasma GIP in response to oral glucose, hence indicating that GIP is not the only incretin hormone [66]. This was confirmed in 1983 by Werner Creutzfeldt's group, who showed that immuno-neutralization of GIP in gut extracts diminished the incretin effect in rats by less than 50% [67] (Figure 1). Around the same time, Pauline K. Lund, Joel Habener and colleagues identified a glucagon-like sequence in the anglerfish preproglucagon cDNA [[68], [69], [70]], followed by identification of two glucagon-like peptides within the preproglucagon sequence of hamsters [71], rats [72,73], and humans [74]. Based on their ∼50% sequence homology to glucagon, the peptides putatively corresponding to these cDNA sequences were named glucagon-like peptide-1 and -2 (GLP-1 and GLP-2) [71]. Independent studies led by Svetlana Mojsov in Boston [75] and Jens J. Holst in Copenhagen [76] then suggested that processing of the C-terminal part of proglucagon gives rise to different products in the intestine (mainly GLP-1 and GLP-2) and the pancreas (mainly the major proglucagon fragment, MPGF), and that N-terminally truncated forms of GLP-1 produced in the intestinal mucosa, GLP-1(7-36)NH2 and GLP-1(7-37), promote insulin secretion in the isolated perfused pancreas of pigs [76] and rats [77], respectively, at low and probably physiological concentrations. Following the demonstration by Daniel Drucker that GLP-1 autonomously stimulates insulin secretion in rat islet insulinoma “RIN” 1046-38 cells [78], Bernhard Kreymann from Steve Bloom's laboratory then showed that physiological concentrations of GLP-1(7-36)NH2 enhances glucose-stimulated insulin secretion in humans [79], hence establishing GLP-1 as the second incretin hormone (Figure 1).

## 4 Localization and characterization of GIP-producing cells

### 4.1 Localization in the intestine

**Figure 2:**
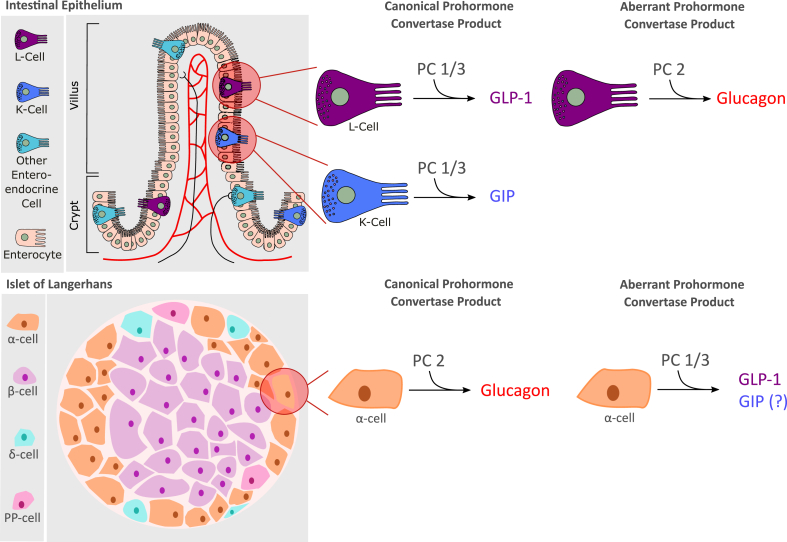
**Processing of incretin hormones by prohormone convertase.** In normal physiology proglucagon is processed into glucagon by prohormone convertase 2 (PC2) in the islet alpha cells, and into GLP-1 by prohormone convertase 1/3 (PC1/3) in intestinal L-cells. ProGIP is processed into GIP by PC1/3 in intestinal K-cells. Under certain conditions intra-islet production of GLP-1 and GIP potentially occurs through increased PC1/3 activity. Glucagon may be aberrantly produced in intestinal L-cells via PC2.

Intestinal localization and characterization of GIP-producing cells were first assessed using immunofluorescence [80] and radioimmunoassays (RIAs) [81], identifying the duodenal and jejunal mucosa as the primary source of circulating GIP in humans and dogs (Figure 2). Ultrastructural and immunocytochemical analysis using intestinal mucosal samples from pigs, dogs and humans then identified the GIP-producing cells as K-cells [82,83]. The density of the K-cells, and hence intestinal secretion of GIP, is highest in the upper intestine with a gradual decrease along the gut, contrasting with GLP-1 producing L-cells which increase in density towards the distal gut [82,[84], [85], [86], [87], [88], [89], [90], [91], [92]]. In line with this distribution of the K-cells, studies in humans that underwent partial intestinal resection due to Crohn's disease accordingly show that the rise in plasma GIP after oral administration of glucose correlates positively with the length of the residual jejunum, but is unaffected by resection of the ileum [66]. Moreover, serum levels of IR-GIP are unchanged in rhesus monkeys with 50% resection of the distal small bowel [93,94]. Although K-cells are the predominant source of GIP in the intestine, as also independently confirmed in mice that express yellow fluorescent protein (YFP) under control of the GIP promoter [95], a small subset of enteroendocrine cells in the small intestine produce both incretin hormones [84]. Interestingly, postprandial levels of serum IR-GIP were reported to be enhanced in people who have undergone pancreaticoduodenectomy (Whipple's procedure), i.e. after resection of the antrum, duodenum, the head of the pancreas and the proximal part of the jejunum [96], but this has not been confirmed in other studies [97]. Potential explanations for the greater rise in serum IR-GIP in these individuals include faster passage of ingested nutrients into the residual jejunum, reduced pancreatic feedback inhibition due to partial pancreatectomy, or decreased pancreatic uptake of GIP due reduced in islet mass [96]. In agreement with this, gastric emptying is enhanced in pancreatectomized individuals [98], and glucagon suppresses GIP secretion [99,100], although a direct inhibitory action of glucagon on K-cells seems unlikely. Several studies further report decreased GIP secretion in response to oral nutrient ingestion under conditions of hyperinsulinemia [101,102], but this is not confirmed by other studies [[103], [104], [105]]. In summary, intestinal GIP is produced in the enteroendocrine K-cells, which show high abundance in the upper intestine with a gradual decrease along the gut.

### 4.2 Localization of GIP expression outside the intestine

Although the intestine is the primary source of circulating IR-GIP [66], GIP immunoreactivity and/or expression has in some studies also been observed in pancreatic α-cells [[106], [107], [108], [109], [110], [111]], especially under conditions of islet stress imposed by pregnancy, high-fat feeding, insulin resistance or β-cell toxins [[112], [113], [114]] (Figure 2). Some studies suggest a function for α-cell-derived GIP [109] and GLP-1 [115,116] in the paracrine stimulation of β-cells, as indicated by studies using incretin receptor knock-out mice [[112], [113], [114]]. It should, however, be noted, that pancreatic production of GIP remains questionable since GIP was not identified in human or mouse pancreatic islets by mass spectrometry [117], nor was GIP mRNA detected in murine α-, β- or *δ*-cells by RNAseq [118,119] or using mice that express YFP under control of the GIP promoter [120]. In any case, even if GIP is produced in the islets, pancreatic GIP is much lower relative to its production in the intestinal mucosa.

GIP is also located in the central nervous system (CNS) [85,[121], [122], [123]], the submandibular salivary gland [86,124], and the stomach [125]. Immunohistochemical studies of the adult rat brain report highest CNS levels of GIP immunoreactivity in the olfactory bulb, hippocampus, and the Purkinje cells in the cerebellum, and moderate levels in the cerebral cortex, amygdala, substantia nigra, hypothalamus and the hindbrain [121]. In the CNS, GIP co-localizes with the neuronal marker NeuN (a.k.a. Fox3), but not with the glial marker glial fibrillary acidic protein (GFAP), excluding astrocytes as a source of GIP [121]. Notably, in contrast to reports indicating that GIP is also expressed in the CNS [85,[121], [122], [123]], mice with expression of yellow fluorescent protein (YFP) under control of the GIP promoter do not show YFP reporter expression in either the pancreas or the brain [120]. In summary, GIP is mainly produced in the upper intestine, with conflicting data regarding its production in the pancreas.

## 5 The incretin effect in people living with obesity and T2D

In healthy humans, the incretin effect accounts for 50–70% of the insulin secretory response to oral glucose [[126], [127], [128], [129], [130]]. The majority of this effect is attributed to the action of GIP and GLP-1, as supported by almost complete abrogation of the incretin effect upon simultaneous antagonization [131,132] or concomitant deletion of the GLP-1 receptor (GLP-1R) and the GIP receptor (GIPR) [133] in mice. Similar results are obtained by using specific antagonists at GIPR and GLP-1R during and after an oral glucose tolerance test, or a mixed meal, in healthy humans [131,134]. Apart from the decreased insulin secretion, double incretin receptor knock-out mice show abnormally high levels of blood glucose and prolonged glycemic excursion after oral glucose challenge relative to wildtype or single incretin receptor KO mice [133]. The quantitative impact of the incretin effect increases in healthy humans with the load of ingested glucose [126,129]. Plasma glucose excursions following oral or intravenously (i.v.) infused glucose further remain similar, in spite of increasing oral dosing, where the oral doses increasingly exceed the i.v. infused doses [129], hence indicating that the incretin effect plays an essential role in maintaining normal glucose tolerance. The incretin effect is most prominent in lean subjects with normal glucose tolerance and tends to be lower in those with oral glucose intolerance or higher than normal body-mass-index (BMI) [135,136]. In individuals with normal glucose tolerance, the incretin effect is decreased in obese relative to lean individuals [137].

As shown in 1986 by Michael A. Nauck, Werner Creuztfeldt and coworkers [127], and later confirmed by other studies [126,138,139], the incretin effect is blunted in people living with T2D, accounting in these individuals for only ∽35%, and often less, of postprandial insulin secretion. Although a large-scale population-based study suggests that meal-induced GLP-1 secretion is slightly reduced in people with T2D [140], fasting or postprandial levels of total and intact GLP-1 and GIP are in most studies not overtly different between individuals living with T2D and healthy controls [126,141,142]. Both incretins are rapidly cleared from the circulation via the kidneys. While enzymatic inactivation of the incretins, which is primarily catalyzed by dipeptidyl-peptidase 4 (DPP4), is not affected by kidney function, circulating levels of the inactive metabolites, GLP-1(9-36)NH2 and GIP (3-42), are increased in people with impaired renal function [143]. In individuals with normal kidney function, elimination rates of GLP-1 [144] and GIP [145] are similar between healthy subjects and those with T2D, indicating that the impaired incretin effect in people living with T2D does not result from impaired secretion or enhanced clearance of the incretins [146]. As demonstrated by Michael Nauck using hyperglycemic clamp studies [144], and later confirmed by other studies [[147], [148], [149], [150], [151]], the insulinotropic effect of GIP is strikingly reduced in people with T2D relative to healthy controls, while the insulinotropic [144,151,152] and glucagon-inhibitory effects [153,154] of GLP-1 are largely preserved. And while both incretins additively increase insulin secretion in healthy humans [155], GIP is unable to enhance GLP-1-induced insulin secretion in subjects with T2D [156]. While these studies support the notion that the impaired incretin effect in individuals living with T2D originates from a diminished insulinotropic action of GIP, the relative contribution of GIP to the incretin effect remains controversial and is potentially species-specific. Some human studies observed that the insulinotropic effect of exogenous GLP-1 is greater relative to exogenous GIP, but GIP was in this study given at less than physiological concentrations [79,157]. Other studies, using higher doses, observed that GIP is the predominant incretin hormone [131,155], or that GLP-1 and GIP nearly equally contribute to the incretin effect [158]. Notably, the relative contribution of the incretins to postprandial insulin secretion is best determined using incretin receptor antagonists, and postprandial insulin secretion is lower upon antagonization of GIPR relative to GLP-1R, hence indicating that GIP is the predominant incretin hormone in humans [134] and mice [159].

An attenuated insulinotropic response to GIP is found not only in individuals living with T2D, but, at least partially, also in their first-degree relatives [128,150]. Impaired GIP-induced insulin secretion is further observed in the setting of chronic pancreatitis, and in individuals with mutations in HNF1α (MODY3 diabetes) [160]. Hence, the impaired insulinotropic action of GIP may reflect impaired β-cell function and may be secondary to diabetes and β-cell failure. Challenging this assumption, however, the late-phase insulin response to GIP is greater in subjects with chronic pancreatitis (CP) and normal glucose tolerance relative to individuals with CP and impaired glucose tolerance, hence indicating that impaired GIP amplification of insulin secretion also develops during the progression of glucose intolerance [161]. The incretin effect is restored in people with type 1 diabetes (T1D) after pancreas transplantation [162]. Since the transplanted pancreas in these subjects is denervated from the autonomic nervous system, this indicates that the incretins promote insulin secretion primarily via endocrine/paracrine rather than neuronal signaling [162]. In agreement with this, the incretin effect is fully preserved in subjects with truncal vagotomy [163].

Several preclinical studies show that the impaired insulinotropic action of GIP under conditions of hyperglycemia is paralleled by decreased expression of the GIP receptor (*Gipr)* in pancreatic islets [[164], [165], [166]]. Expression of *Gipr* is decreased in rat insulinoma INS(832/13) cells under conditions of high glucose [167], and in islets of hyperglycemic Vancouver Diabetic Fatty (VDF) Zucker rats [165], but is restored in VDF rats upon normalization of glycemia [165]. Other studies using clonal β-cells suggest possible additional effects involving desensitization of distal steps in the stimulus-exocytosis cascade [168,169]. GIP-induced cAMP production is blunted when rat or human pancreatic islets are cultured under conditions of high glucose, and treatment of isolated islets with the proteasomal inhibitors lactacystin or MG-132 prevents glucose-stimulated downregulation of *Gipr*, and preserves GIP-induced cAMP production, suggesting that hyperglycemia promotes degradation of GIPR by stimulating its ubiquitination [170]. The expression of *Gipr* is subject to differential splicing in the β-cells under conditions of hyperglycemia, ultimately influencing GIP sensitivity in diet-induced obese (DIO) mice [171]. Consistent with glucose-stimulated defects in GIP responsiveness, individuals with pancreatitis show higher levels of blood glucose relative to healthy controls, and fail to appropriately increase plasma insulin levels after oral glucose challenge despite rapid elevation of IR-GIP [172]. Near-normalization of hyperglycemia through 4 weeks of insulin therapy [173], or through administration of sulphonylurea [174] or DPP4 inhibitor [175] improves the insulinotropic effect of GIP in people living with T2D, supporting that GIPR agonism may offer pharmacological benefits on glucose control when given together with drugs that decrease hyperglycemia. The same principle may potentially also account for the observation that unimolecular GIPR:GLP-1R co-agonists improve glucose control with superior efficacy relative to GLP-1R agonism alone in preclinical [176,177] and clinical [[178], [179], [180]] studies. However, in patients with T2D, normalization of blood glucose through a 6 h GLP-1 infusion is not capable of restoring GIP responsiveness [156], and the insulin response to GLP-1 and GIP co-infusion is indistinguishable from GLP-1 treatment in individuals with obesity during isoglycemic i.v. glucose infusion [181]. In summary, the decreased incretin effect in patients with T2D is attributed mainly to an impaired insulinotropic action of GIP, potentially because of impaired β-cell function and down-regulation, degradation or alternative splicing of the GIP receptor under conditions of hyperglycemia. Nonetheless, in individuals living with T2D, the insulin secretory response to GIP is impaired in only the late, but not the early phase of insulin secretion, suggesting that a GIPR defect unlikely accounts for the perturbed insulinotropic action of GIP in these subjects [151,160,182].

## 6 Transcriptional regulation of GIP production

The GIP promoter region contains two binding sites for cAMP response element binding protein 1 (CRE-BP1), and while both of them are required for basal GIP promoter activity, only the one at position −158 bp is required for the cAMP-inducibility of the GIP promoter [183]. In rodents, duodenal expression of *Gip* is induced upon duodenal perfusion with a lipid-meal [86], and upon oral administration of either glucose or corn-oil [184]. Nutrient-regulation of GIP expression seems to be under control of the transcription factor c-Jun, which is upregulated in HIT T15 cells under conditions of glucose deprivation [185], and which suppresses cAMP-inducibility of the GIP (and insulin) promoter [183,185]. Studies in hamster insulinoma HIT T15 cells transfected with human GIP reporter genes show that transcription may be induced by increased level of cAMP, and that the ∽180 bp region prior to the transcription start of the GIP gene is sufficient for basal GIP transcription [183].

Transcriptional control of GIP inevitably depends on the functionality of the K-cells, which are continually produced from pluripotent stem cells located in the crypts of the intestinal gut epithelium [186]. Like other enteroendocrine cells, the development of the K-cells is under tight control from a variety of transcription factors, which determine the fate of the stem cells during cellular differentiation. Transcription factors affecting K-cell development include *pancreatic and duodenal homeobox 1* (*Pdx1*), paired box gene 4 (*Pax4*) and 6 (*Pax6*), and *aristaless-related homeodomain transcription factor* (*Arx*). Mice deficient for any of these factors show strikingly reduced GIP immunoreactivity in the intestine [[187], [188], [189], [190]]. Beyond its role in K-cell differentiation [188], PDX1 enhances *Gip* expression via binding to the *Gip* promoter, as demonstrated by enhanced *Gip* reporter activity in mouse intestinal tumor STC-1 cells transfected to overexpress *Pdx1* [189]. Knock-down of *Pdx1* in STC-1 cells further decreases the expression, cellular content and secretion of GIP [191]. The number of intestinal K-cells, as well as expression and secretion of GIP, is elevated in old relative to young mice, and this correlates with enhanced expression of *Pdx1* [192]. Although the GIP response to oral glucose may be exaggerated in the elderly relative to middle aged humans [193,194], a meta-analysis comprising 22 studies shows that the GIP secretory response to oral glucose, or to a mixed meal, is reduced with increasing age [141]. Nonetheless, in line with a role for PDX1 in age-related changes in GIP secretion, suppression of PDX1 using intestine-specific gene transfer in mice reduces K-cell number, *Gip* expression and GIP content in the small intestine [192]. Other transcription factors known to stimulate *Gip* expression through binding to the GIP promoter include *islet-1* (*Isl-1*) [195] and *GATA binding protein-4* (*Gata-4*) [189,195]; ISL-1 and GATA-4-mediated activation of the GIP promoter accounts for 85–90% of *Gip* expression in STC-1 cells [195]. Suppression of GATA-4 in GIP-producing GTC-1 cells, a subclone of STC-1 cells, decreases GIP promoter activity, while its overexpression in mouse insulinoma βTC-3 cells increases expression and secretion of GIP [196]. Microarray analysis further identified the transcription factor *Regulatory factor X6* (*Rfx6*) to be exclusively expressed in K-cells in the gut, and knockdown of *Rfx6* in STC-1 cells decreases expression, cellular content and secretion of GIP [191]. *Rfx6* binds to the *Gip* promoter region, and when overexpressed in STC-1 cells, it increases *Gip* expression without affecting *Pdx-1* expression [191]. Loss-of-function variants of *RFX6* are further associated with an unclassified form of MODY diabetes, and heterozygous carriers of such variants exhibit reduced circulating levels of GIP at baseline and after oral glucose ingestion [197].

Of note, the transcriptional machinery regulating K-cell differentiation is similar to that of pancreatic β-cells, and since secretion of GIP increases in response to food intake [26,101,103,[198], [199], [200], [201], [202], [203], [204], [205], [206], [207], [208], [209]], this spurred interest to ectopically express insulin in the K-cells. Genetic reprogramming of K-cells via viral introduction of *Neurogenin3* and *Nkx6.1* induces expression of insulin in K-cells [210], and transgenic mice expressing the human *insulin* gene under control of the rat *Gip* promoter show *insulin* expression targeted to intestinal K-cells [211]. Such mice with K-cell-specific insulin production are protected from development of streptozotocin (STZ)-induced diabetes and show preserved glucose tolerance upon such chemical destruction of the β-cells [211]. In summary, the GIP promoter is regulated by cAMP and a variety of transcription factors that also regulate K-cell development and function. Data on age-related changes of GIP secretion are partially conflicting, with most data showing age-related reduction of GIP secretion in response to oral glucose or a mixed meal.

## 7 Translational regulation of GIP production

**Figure 3:**
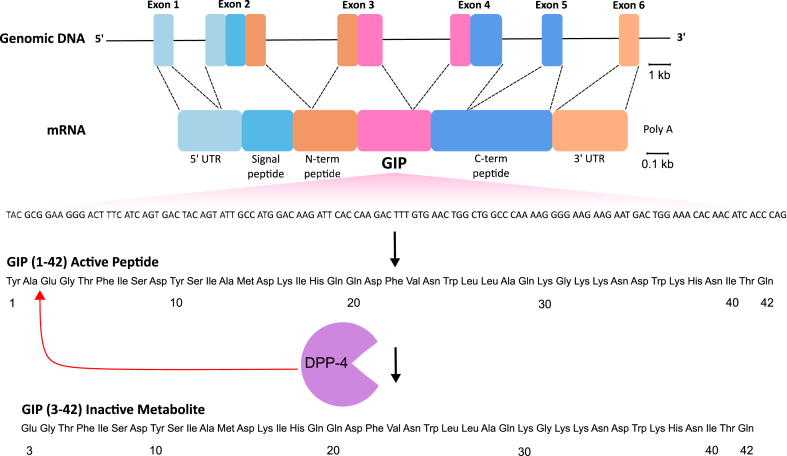
**The genetic encoding and peptide processing of GIP.** GIP in encoded by the *GIP* gene on chromosome 17, consisting of six exons. The majority of the sequence encoding GIP peptide is localised to exon 3 (highlighted in pink). A 153-amino acid long proGIP precursor is processed by prohormone convertase 1/3 to produce bioactive GIP1-42. GIP1-42 is rapidly degraded by dipeptidyl peptidase-4 (DPP4) into the inactive GIP3-42.

The amino acid sequence of porcine GIP was reported in 1971 [212,213], but was revised a decade later [214], now establishing intestinal-derived GIP as a 42 amino acid polypeptide, GIP(1-42). The peptide is derived through posttranslational cleavage of pro-GIP by the action of the prohormone convertase 1/3 (PC1/3), which is encoded by the *PCSK1* gene [215,216] (Figure 3). Pro-GIP comprises 144 (rodent) or 153 (human) amino acids and harbors the GIP sequence flanked by N- and C-terminal peptide sequences [86,[217], [218], [219], [220], [221], [222]]. Pro-GIP possesses cleavage sites for PC1/3 at the positions at P^21^R^22^↓Y^23^ and Q^64^R^65^↓E^66^, and has, other than GIP, no known functional cleavage products [86,[216], [217], [218]]. Notably, the C-terminal residue of GIP(1-42) is Q^64^, so PC1/3 cleavage after R^65^ suggests that there is another proteolytic event which removes R^65^ to generate the 42 amino acid polypeptide, likely to be carboxypeptidase E mediated in analogy to general prohormone processing [223]. Although the vast majority of circulating GIP originates from PC1/3 cleavage of pro-GIP, and hence refers to the 42-mer polypeptide [216], pro-GIP also contains a cleavage site for PC2 at position K^52^G^53^K^54^↓K^55^ [216]. PC2-mediated cleavage of GIP(1-42) at this position and subsequent processing by peptidylglycine α-amidating monooxygenase (PAM) leads to generation of GIP(1-30)NH2, which is in some studies found in pancreatic α-cells [109,224] (Figure 2) and accounts for ∽5–15% of murine GIP immunoreactive cells in the intestine [215]. Seemingly in line with the generation of GIP(1-30)NH2 in the islet α-cells [109] is the observation that intraislet IR-GIP is only found using antibodies that recognize the GIP middle or N-terminus, but not when using antibodies that recognize the GIP(1-42) C-terminus [109,215,225]. Nonetheless, expression of GIP(1-30)NH2 in the pancreatic islets is questioned by other studies, which failed to find *Gip* expression in the α-cells by scRNAseq analysis [119], and no GIP transcriptional activity is being detected in the islets using mice that express YFP under control of the GIP promoter [120]. Low amounts of GIP(1-30)NH2 are further also found in people that underwent total pancreatectomy [226], indicating that GIP(1-30)NH2 is produced in the intestine rather than the islets.

In the mouse intestine, GIP is highly co-localized with PC1/3, but not with PC2, and pro-GIP processing to GIP(1-42) is severely impaired in mice deficient for PC1/3, but largely preserved in mice deficient for PC2 [216]. Furthermore, overexpression of pro-GIP in mouse pituitary AtT-20 cells, which show high expression of PC1/3 and low expression of PC2, robustly induces GIP production [216]. Interestingly, however, processing of GIP and GLP-1 is largely preserved in an individual living with a homozygous loss-of-function mutation in *PCSK1* [227]. However, although the vast majority of circulating GIP originates from PC1/3 processing of pro-GIP into GIP(1-42), studies in mouse pancreatic α-TC1.9 cells, which express PC2 but not PC1/3, show that PC2 cleavage of pro-GIP gives rise to a C-terminally truncated GIP, most likely GIP(1-31) [216], which is then under *in vivo* conditions further processed to GIP(1-30)NH2 [109,215]. Notably, pro-GIP needs to be cleaved at two positions to generate GIP. Generation of GIP(1-31) in α-TC1.9 cells is hence only possible if there is either residual PC1/3 activity or if PC2 also cleaves pro-GIP at the position P^21^R^22^↓Y^23^, or if this site is cleaved by another protease encountered during prohormone maturation. Although GIP(1-30)NH2 is as potent as GIP(1-42) in GIPR binding and cAMP production [228], and to stimulate insulin secretion in either the perfused mouse pancreas [109], rat islet insulinoma RIN1046-38 cells [229], mouse pancreatic *β*TC-3 cells [229], and healthy human subjects [230], its circulating levels are only ∽1–3 pmol/l, which is ∽15 fold lower than the fasting GIP(1-42) serum concentration [226,231]. Levels of GIP(1-30)NH2 further rise only marginally after oral glucose administration [226,231], hence indicating that GIP(1-30)NH2 represents only a minor fraction of active GIP in the general circulation. However, enzyme resistant analogues of the two peptides are equally effective in diabetic HFD-fed mice, suggesting possible therapeutic utility of this truncated form of GIP [232]. Collectively, GIP(1-42) is processed through proteolytic cleavage of pro-GIP by PC1/3, and while expression of GIP(1-30)NH2 in the islet α-cells remains controversial, GIP(1-30)NH2 represents only a small fraction of active GIP in the circulation, and as such makes no major contribution to the regulation of blood glucose under physiological conditions.

## 8 Degradation of GIP

In 1987, Wolfgang Schmidt observed, using HPLC analysis, that commercially available preparations of porcine GIP(1-42) contain a considerable amount of N-terminally truncated GIP(3-42) [233]. In isolated rat islets, GIP(3-42) does not increase insulin secretion under high glucose conditions [233,234], or affect insulin secretion induced by GIP(1-42) [233], suggesting that it may be a biologically inactive metabolite of GIP(1-42). Subsequently, exposure of GIP(1-42) to human [235,236] or rat [237] serum revealed that the intact peptide was rapidly (within minutes) cleaved into GIP(3-42), with the enzyme responsible being identified as dipeptidyl peptidase 4 (DPP4).

GIP(1-42) is rapidly cleaved into GIP(3-42) when incubated with purified DPP4 [235], but is preserved in the serum of DPP4 deficient rats [237] or mice [238], or when co-incubated in serum along with the DPP4 inhibitors Lys-pyrrolidine or diprotin A [235]. GIP is extensively degraded *in vivo*, with the intact biologically active peptide accounting for 37% of circulating IR-GIP in humans [239], and only 15% in pigs [240], and accordingly, both its levels and insulinotropic effects are increased following DPP4 inhibition in humans [241] and pigs [240]. The estimated plasma half-life of active GIP in the general circulation is ∽7 min [143,239,242], which is ∽5 fold greater than the half-life of GLP-1 [143,239,242]. Thus, GIP is first converted to GIP 3-42, and is then cleared from the circulation via the kidneys, and C-terminally detected GIP vanishes from the circulation with a t_1/2_ of ∽16–27 min [239], corresponding roughly to glomerular filtration. Supporting a role of the kidneys in GIP clearance, circulating levels of total GIP are lower in the renal relative to the hepatic and femoral vein [243], and are elevated in people living with impaired renal function [143,[244], [245], [246], [247]] and in nephrectomized rats after intraduodenal infusion of glucose [243]. Studies in rats and dogs find no evidence that hepatic extraction contributes to GIP clearance [248,249], and this is confirmed in pigs using a C-terminally directed assay [240]. However, significant clearance across the liver is found using an assay which detects the intact N-terminus of GIP, and which is eliminated during DPP4 inhibition [240]. This suggests that hepatic degradation of GIP does occur, and is dependent on the action of DPP4, consistent with its presence in high concentrations on hepatocytes [250]. Since GIP(3-42) is the major form in the circulation, studies in human and rodent models have investigated it's possible biological significance, revealing that the metabolite is inactive or a weak GIPR antagonist in rodents [251,252], pigs [251] and humans [253]. However, recent studies suggest a possible role of GIP(3-42) to promote β-cell health under conditions of functional islet stress [254]. In summary, GIP(1-42) represents the main bioactive form of GIP in the circulation, which is degraded by DPP4 into GIP(3-42) and cleared via the kidneys with a t_1/2_ of ∽16–27 min.

### 8.1 DPP4 inhibition and GIP

With the realization that GLP-1 and GIP were rapidly degraded in the circulation by DPP4, Carolyn Deacon and colleagues from the University of Copenhagen were quick to realize that inhibitors of the enzyme might represent a new therapeutic avenue to potentially treat T2D by enhancing incretin action [255]. Much activity followed and although the precise sequence of events is disputed [256,257], the first member of a new family of DPP4 inhibitor drugs was approved in 2006 (Figure 1). For many years, the effectiveness of DPP4 inhibitors to treat 2TD was solely attributed to increased bioactivity of GLP-1 but it is now clear that GIP also contributes significantly to the glucose-lowering action of these inhibitors. The GLP-1R antagonist exendin(9-39) blocks approximately 50% of the glucose-lowering and insulinotropic effects of DPP4 inhibition [258] and DPP4 inhibition leads to a much greater increase in bioactive GIP than GLP-1 in humans [259]. Studies using KO mice lacking the receptors for GIP, GLP-1 or both also demonstrate an important contribution from both incretin hormones [133,260,261]. Furthermore, recent studies using the GIPR antagonist GIP(3-30)NH2 in patients with T2D established that endogenous GIP makes a substantial contribution to β-cell function and blood glucose control in the presence of DPP4 inhibitors [262]. Although once doubted, this is consistent with expression of a fully functional GIPR and the ability to restore the actions of GIP in patients with T2D [175].

### 8.2 Early development of stable DPP4-resistant GIP analogues

Generation of stable forms of GIP was an attractive alternative to DPP4 inhibition and the first DPP4-protected GIP analog was reported by Peter Flatt and colleagues from Ulster University, Coleraine in 1998/9 [263] (Figure 1). The molecule was protected from DPP4 recognition via N-terminal glycation of the Tyr(1) residue, and had enhanced insulinotropic action in rat pancreatic insulinoma BRIN-BD11 cells relative to native GIP [264]. The group subsequently developed and characterized a family of GIP analogues with N-terminal amino acid substitutions/modifications [[264], [265], [266], [267], [268]]. Specific modifications at the N-terminal positions one or two resulted in DPP4 resistance that led to enhanced insulin secretion in BRIN-BD11 cells and improvement of glucose tolerance in ob/ob mice. In contrast, some amino acid substitutions at position 3 gave weak DPP4-resistant forms that were either not more biologically active compared to native GIP, or weakly antagonized the action of native GIP [[269], [270], [271]]. In contrast, Pro(3)GIP was fully DPP4-resistant and strongly antagonized the actions of native GIP at physiological concentrations both *in vitro* and *in vivo* [159,270,272], while Hyp(3)GIP exhibited intermediate activity [271]. The Vancouver group led by Chris McIntosh was also active in this area for some time and focused attention on DPP4-resistance and the particular benefit of d-Ala(2) and Ser(2) substitutions in GIP [[273], [274], [275]]. To improve pharmacokinetics by circumventing rapid renal filtration, the Coleraine group were instrumental in developing second generation stable GIPR agonists and antagonists using fatty acid acylation or PEGylation strategies [[276], [277], [278], [279], [280], [281], [282], [283], [284], [285]]. These peptides exhibited good therapeutic utility in type 2 diabetic animal models and, together with Amylin Pharmaceuticals, some of these long-acting GIPR agonists entered the therapeutic pipeline in 2006 [286]. Despite the identification of GIPR as a therapeutic target, the program was halted by Amylin Pharmaceuticals in 2011. Thereafter the Coleraine team demonstrated that longer-acting acylated forms [287] rather than solely DPP4-resistant forms [288,289] in co-treatment of obese ob/ob mice with GLP-1R and GIPR agonists improved glycemic control relative to either agonist alone, but with limited ability to induce short-term body weight loss [[287], [288], [289]]. These early studies at Coleraine promoted discovery of long-acting peptides capable of activating both GIP and GLP-1 receptors [290] or triple action peptides signaling additionally at the glucagon receptor [[291], [292], [293]]. These novel analogues were shown to exhibit beneficial effects in HFD-fed mice, but they were highly imbalanced in receptor activity with relatively low potency, which compromised the beneficial virtue in coordinated agonism at each receptor. Subsequent, *in vivo* studies with fully balanced, high-potency peptides demonstrated the superior pharmacology achievable when simultaneously activating two or three receptors [177,294,295].

## 9 Regulation of GIP secretion

### 9.1 Regulation of GIP secretion by fasting and feeding

The first RIAs to detect circulating GIP were reported in the mid 1970's [198,296] (Figure 1), but the assays were plagued by low specificity due to cross-reactivity of the antisera with GIP-like moieties of various sizes and other serum components [[297], [298], [299], [300], [301], [302]]. The use of antisera against porcine GIP, which differs from the human peptide at positions 18 and 34, and the use of porcine GIP as standard in these RIAs, further reduced the comparability and reproducibility of measurements using these assays [[297], [298], [299], [300], [301]]. In healthy humans, RIAs optimized for quantification of the 5 kDa human polypeptide show that serum levels of total IR-GIP are <20 pmol/l in the fasting state, and rapidly increase up to ∽300 pmol/l within the first hour after the ingestion of a standard meal [301,303,304]. Circulating levels of total IR-GIP rise sharply within 30 min after a standard meal [96,198,302,304,305], or when nutrients are placed directly into the duodenum [[306], [307], [308]], and remain elevated for several hours depending on the meal size and composition. Secretion of GIP is greater after consumption of high vs. low caloric meal [309], and is further augmented when a small protein load (55g whey) is ingested 30 min prior to a mixed meal [[309], [310], [311]]. Meal-induced GIP secretion after dinner is nonetheless unaffected by whether breakfast or lunch has been eaten, and also the speed of meal intake does not seem to affect GIP secretion [309]. Yet, while the GIP response to oral glucose does not depend on the temperature of the glucose load, meal-induced GIP secretion is somewhat higher at lunch time relative to when identical meals are eaten in the morning or the afternoon [309], potentially suggesting adaptation to the rate of gastric emptying, alterations in K-cell nutrient sensitivity, or carry-over effects from previous meals.

A limitation of most GIP-detecting RIAs is that they used antisera recognizing either the middle or C-terminus of the peptide, and hence measure total GIP without discriminating between the active peptides and their N-terminally truncated metabolites. The first RIA capable of detecting the GIP N-terminus was developed in 2000 by Carolyn Deacon and Jens Holst [239] (Figure 1). Comparative analysis of IR-GIP using N- and C-terminal detecting RIAs revealed that active GIP accounts for ∽40–50% of circulating total GIP under baseline [312,313], and postprandial conditions [239,[312], [313], [314]]. In summary, the first assays used to detect GIP faced issues with low specificity and reliability. Optimized RIAs show GIP levels of <20 pmol/l during fasting, and of ∼300 pmol/l after ingestion of a standard meal in healthy humans. Meal-induced secretion of GIP is influenced by the caloric content and enhanced by protein preloads but is unaffected by meal timing or the speed of food intake.

### 9.2 Regulation of GIP secretion by nutrients

**Figure 4:**
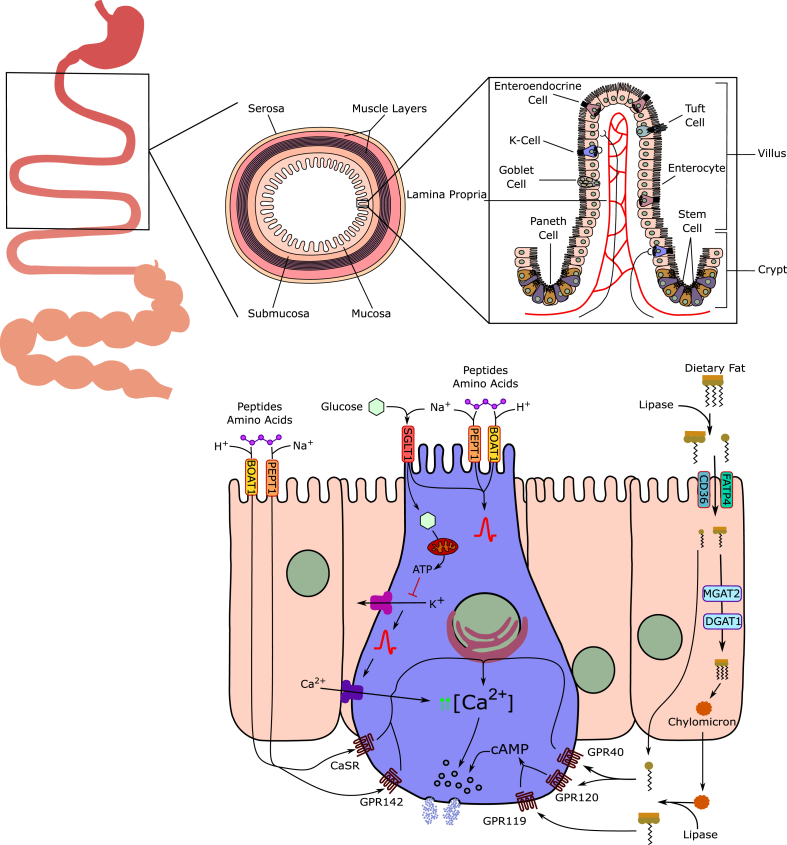
**Mechanisms underlying the release of GIP from intestinal enteroendocrine cells.** GIP is released from enteroendocrine K-cells that line the epithelium of the small intestine. K-cells span the crypt–villus axis and are equipped to sense nutrients and regulatory signals from both their luminal and basolateral surfaces. Dietary glucose is sensed through SGLT1 which couples glucose transport with sodium influx, thereby depolarising the cell membrane. Glucose is then metabolised to ATP, causing K_ATP_ channel closure, further membrane depolarisation, and the subsequent opening of voltage-gated calcium channels (VGCC). Rising intracellular Ca^2+^ concentrations stimulate GIP secretory vesicle release. Dietary oligopeptides and amino acids are transported into K-cells via sodium-coupled PEPT1 or proton-coupled BOAT1, resulting in membrane depolarisation. Amino acids and oligopeptides absorbed by neighbouring enterocytes may also act on the basolateral surface of K-cells through binding G_q_-coupled GPCRs including CaSR and GPR142. CaSR and GPR142 binding stimulate the release of calcium from intracellular calcium stores, thereby increasing intracellular Ca^2+^ concentrations and promoting GIP vesicle release. Dietary fat is mainly sensed post-absorption at the basolateral surface of K-cells. Fatty acid transporters CD36 and FATP4 mediated free fatty acid (FFA) uptake by neighbouring enterocytes. FFAs are reesterified into triacylglycerols (TAGs) via the enzymes MGAT2 and DGAT1. TAGs are released from enterocytes, possibly as chylomicrons, to the basolateral side of the epithelium. Here, monoacylglycerols (MAGs) and long-chain fatty acids (LCFAs) bind GPCRs on K-cells to elicit GIP vesicle release. LCFA binding of GPR40 (FFAR1) or GPR120 (FFAR4) recruits Gαq, stimulating the release of calcium from intracellular calcium stores. Alternatively, MAG binding of GPR119 signals via the G_s_-coupled pathway, stimulating cAMP levels and recruiting PKA and EPAC signalling pathways to enhance GIP vesicle release. B0AT1, neutral amino acid transporter 1; cAMP, cyclic adenosine monophosphate; CaSR, calcium-sensing receptor; CD36, cluster of differentiation 36; DGAT1, diacylglyceride acyltransferase 1; EPAC, exchange protein directly activated by cAMP; FATP4, fatty acid transporter protein 4; GPCR, G-protein-coupled receptor; LCFA, long-chain fatty acid; MAG, monoacylglycerol; MGAT2, monoacylglyceride acyltransferase 2; PKA, protein kinase A; PEPT1, peptide transporter 1; SGLT1, sodium-coupled glucose cotransporter 1; TAG, triacylglycerol; VGCC, voltage-gated calcium channel.

As demonstrated in humans, dogs, pigs, mice and rats, circulating levels of total GIP increase upon oral or intraduodenal administration of fat [26,[198], [199], [200], [201], [202], [203], [204],^315^,^316^], glucose [26,101,103,198,[200], [201], [202],[204], [205], [206], [207], [208], [209],^317^], galactose [200,317], and leucine [198] (Figure 4). Although not confirmed in every study [201], the rise in serum IR-GIP is slower, but greater and more prolonged, upon ingestion of fat relative to glucose [26,198,200], and this is confirmed also when equicaloric amounts of glucose or fat are administered directly into the duodenum [300]. The slower rise in plasma GIP following ingestion of fat relative to glucose might be attributed to delayed gastric emptying, which is typically observed following ingestion of fat [14,15,318]. Complex protein meals, such as steak [198] or steamed cod [319] do not stimulate GIP secretion, but circulating levels of GIP increase upon intraduodenal administration of individual amino acids or protein hydrolysates in dogs [320] and humans [202,[321], [322], [323]] (Figure 4). In humans, intraduodenal perfusion with a perfusate containing arginine, histidine, isoleucine, leucine, lysine, and threonine leads to a greater rise in serum GIP and insulin relative to a perfusate containing methionine, phenylalanine, tryptophan, and valine [322]. It has also been shown that secretion of GIP is stimulated by oral but not i.v. administration of an amino acid mixture [324]. Peptone, a protein hydrolysate, increases circulating levels of GIP in dogs [325], rats [326], and humans [323]. In ob/ob mice, a model of obesity-diabetes with markedly elevated levels of circulating GIP, functional K-cell hyperplasia and intact β-cell GIP responsiveness [316,327,328], a range of essential and nonessential neutral and basic amino acids, including arginine, cysteine, histidine, alanine, hydroxyproline and lysine increased plasma levels of GIP [329]. In primary K-cell cultures, secretion of GIP increases upon treatment with glucose, glutamine, and linoleic acid, and this is further potentiated by elevation of cAMP using forskolin plus 3-isobutyl-1-methylxanthine (IBMX) [95]. Glucose, sucrose, galactose, and 3-O-methylglucose, 2-deoxyglucose and alpha-methyl-glucoside enhance GIP secretion in ob/ob mice or the isolated perfused rodent intestine, while mannose, 6-deoxygalactose, N-acetyl glucosamine, myoinositol, fructose and lactose have no effect [317,330]. Although not confirmed in every study [331], fructose shows only negligible effects on GIP secretion in mice, rats, dogs, and humans [332,333], but slightly (1.25 fold) elevates GIP secretion in primary K-cell cultures [95]. Consumption of artificial sweeteners, such as sucralose or a tagatose/isomalt mixture, does not affect GIP release in healthy human subjects [334,335]. Collectively, GIP secretion is stimulated by oral or intraduodenal administration of fats, sugars, and specific amino acids, while complex proteins and artificial sweeteners have minimal or no effect.

Ingestion of fat increases plasma levels of GIP by stimulating the expression and secretion of GIP [184] (Figure 4). A short-lasting increase in plasma GIP is further observed in healthy human subjects upon CCK-induced gallbladder emptying, hence indicating that GIP secretion may also be stimulated by the passage of bile into the intestine [336]. Lipid-induced secretion of GIP varies depending on the length and type of the fatty acid and is typically greater and more prolonged upon ingestion of long-relative to short-chain fatty acids, and upon ingestion of monounsaturated relative to saturated fatty acids [316,337,338]. In healthy humans, ingestion of olive oil, which comprises 74% monounsaturated fatty acids, induces a greater and more prolonged rise in plasma GIP and GLP-1 relative to the ingestion of butter, which comprises 72% saturated fatty acids [338]. As shown in obese *ob/ob* mice, the saturated short-chain fatty acids propionic acid (C3:0) and capric acid (C:10:0) are unable to induce GIP secretion, while the long-chain saturated fatty acid stearic acid (C18:0) moderately increases plasma GIP levels [316]. In *ob/ob* mice, the greatest effect on GIP secretion is observed after treatment with the unsaturated long-chain fatty acids oleic acid (C18:1), linoleic acid (C18:2) and linolenic acid (C18:3) [316]. In healthy humans, an oral load of triglycerides leads to a greater rise in plasma GIP relative to long-chain fatty acids, and no impact on GIP secretion is observed upon ingestion of glycerol or medium-chain triglycerides [339]. In agreement with the ability of dietary lipids to enhance expression and secretion of GIP, an increase in intestinal K-cell density is observed in high-fat diet (HFD)-fed obese *ob/ob* mice relative to chow-fed *ob/ob* controls, and this is paralleled by elevated levels of GIP in the plasma and the intestine [340]. In summary, fat-stimulated secretion of GIP is greater after ingestion of long-chain and monounsaturated fatty acids relative to saturated short-chain fatty acids and medium-chain triglycerides.

Apart from the nutrient composition of a meal, GIP secretion, and subsequently the rise in plasma insulin, also depend on the meal size and further on the rate of intestinal nutrient absorption [341,342]. In line with this notion, plasma responses of GIP and insulin to an oral load of glucose (25 g, 75 g or 125 g) are greater and more prolonged with the higher relative to the lower glucose load in both people with T2D and healthy controls [343]. The area under the GIP time-response concentration curve is further increased upon ingestion of a meal containing 80 g fat relative to one containing 20 g fat [342], an observation that might also be attributed by a greater passage of bile into the intestine [336]. In healthy humans, rapidly-absorbed carbohydrates cause a greater and more prolonged rise in plasma GIP relative to slowly-absorbed carbohydrates [203], and the rise in plasma GIP following ingestion of a standard meal is decreased by co-ingestion of guar gum [344] and lower in individuals with tropical malabsorption [341] or with coeliac disease [345] relative to healthy controls. Collectively, GIP secretion is influenced by the type of nutrients, the rate of nutrient absorption, and the meal size. Fat stimulated GIP secretion is slower but more prolonged compared to glucose, with long-chain and unsaturated fatty acids inducing the greatest stimulatory effects.

### 9.3 Cellular mechanisms regulating GIP secretion in response to carbohydrates

With their primary location within the duodenal and jejunal epithelia, the GIP-secreting K-cells are well positioned to rapidly respond to ingested nutrients. The apical surface of the K-cells opens directly into the gut lumen, where they have direct contact with ingested nutrients [82,83] (Figure 4). K-cells express a glucoregulatory machinery similar to that of the pancreatic β-cells, including glucokinase [95,211] and K_ATP_ channels with the Kir6.2 and Sur1 subunits [95,346], although it does not appear that the K_ATP_ channel pathway underlies the stimulation of GIP secretion after glucose ingestion. Glucose is actively transported into the K-cells via the sodium-glucose linked transporter 1 (SGLT1) [95,347], which is located at the apical side of the epithelium that faces the gut lumen [[347], [348], [349]] (Figure 4). The molecular events leading to glucose-stimulated GIP secretion include rapid uptake of glucose with sodium into the K-cells via SGLT1, leading to membrane depolarization and opening of L-type voltage-dependent Ca^2+^ (VDC) channels. The resulting Ca^2+^ influx then triggers vesicular exocytosis and secretion of GIP into the circulation [350] (Figure 4). Consistent with this, GIP secretion is rapidly induced when duodenal and jejunal cell preparations of dogs [351], or murine primary small intestinal cultures [352] are exposed to membrane depolarizing stimuli, such as potassium (K^+^) or the Ca^2+^ ionophore A23187. Secretion is also increased after elevation of cAMP using forskolin or IBMX [352]. Further supporting the key role of SGLT1 in K-cell glucose entry and cell depolarization, oral administration of 3-O-methylglucose, a nonmetabolizable SGLT1 substrate, stimulates GIP and GLP-1 release in healthy humans [335], and the glucose-stimulated rise in plasma GIP is strikingly diminished in mice deficient for SGLT1 [353], and is abrogated in rats [209], mice [346] and in the isolated perfused rat intestine [330] upon administration of the SGLT1 inhibitor phlorizin. Reduced plasma levels of GIP are also observed in obese humans after 12-wks treatment with the SGLT1/2 inhibitor licogliflozin [354]. Surprisingly, as demonstrated in STC-1 cells [355,356] and GIP/Ins cells [356], K_ATP_ channels do not seem to play a major role in glucose-stimulated GIP secretion, and accordingly, treatment with sulfonylureas does not affect glucose-stimulated GIP secretion in humans [357,358]. GIP secretion in response to oral glucose is further fully preserved in K_ATP_ channel deficient *Kir6.2* KO mice [56]. Moreover, while insulin secretion induced by oral glucose administration is preserved in *Kir6.2* deficient mice, it is almost completely abrogated in mice with concomitant deletion of *Gipr* [359]. Although the K_ATP_ channel inhibitor tolbutamide slightly enhances GIP secretion in cultured K-cells [95], treatment of mice with the sulfonylurea glimepiride does not induce GIP secretion [346]. The ability of tolbutamide to stimulate GIP secretion further vanishes upon co-treatment with forskolin [360,361], suggesting that tolbutamide may additionally affect GIP secretion independent of K_ATP_ channel activity, e.g. by direct interaction with Epac2. It is important to note that GIP is secreted when glucose or other carbohydrates are absorbed. The mere presence of glucose or more complex carbohydrates in the gut lumen does not induce GIP secretion. As an example, a sucrose load leads to a GIP response, but when an α-glucosidase inhibitor, such as acarbose, is administered to prevent the breakdown of sucrose into absorbable monosaccharides (glucose and fructose), the GIP response is blunted [[362], [363], [364]]. Similar results were reported when using voglibose [364]. Collectively, the intestinal K-cells respond to ingested nutrients via mechanisms involving SGLT1-mediated glucose uptake, membrane depolarization, and Ca^2+^ influx to promote GIP secretion. Although K_ATP_ channels are expressed in K-cells, they do not seem to play a major role in glucose-stimulated GIP secretion.

### 9.4 Cellular mechanisms regulating GIP secretion in response to proteins

The mechanisms underlying protein-stimulation of GIP secretion are not well understood. While protein hydrolytes (peptone) or mixed amino acid solutions stimulate GIP secretion in humans [321,322], ingestion of more complex protein-rich meals, like steak [198] or steamed cod [319], does not affect GIP secretion. Notably, intact proteins are only poor stimuli of gastrin release and gastric acid secretion, while protein hydrolysates of the same peptides are potent gastrin and gastric acid secretagogues [[365], [366], [367]]. These data hence indicate that protein-stimulation of GIP secretion may originate from the acid-stimulatory properties of the hydrolyzed proteins [325]. Consistent with this, although not confirmed by every study [368], oral or intraduodenal administration of HCl increases circulating or duodenal levels of GIP in rats [326,369] and humans [369,370], and pretreatment of rats with omeprazole, an inhibitor of gastric acid secretion, attenuates peptone-stimulated GIP secretion [325,326]. Transport of amino acids via sodium-coupled PEPT1 or proton-coupled BOAT1 will also serve depolarize K-cells, thereby promoting GIP release. Protein triggered GIP secretion may also involve the G protein-coupled receptors GPR142 and the calcium sensing receptor (CASR) (Figure 4). GIP secretion triggered by oral tryptophan is abolished in GPR142 KO mice [371], and in pig duodenal perfusion studies, GIP secretion triggered by phenylalanine is impaired by the CASR antagonist NPS 2143 [372].

### 9.5 Cellular mechanisms regulating GIP secretion in response to lipids

The ability of the K-cells to secrete GIP in response to ingestion of fat depends on the hydrolytic breakdown of triglycerides, and hence the function of gastric and pancreatic lipases. People living with cystic fibrosis show exocrine pancreas deficiency, and hence display impaired pancreatic lipase secretion and activity, and this is associated with maldigestion and, consequently, increased excretion of non-digested fat (steatorrhea) [373]. Fasting levels of GIP are normal in these individuals but fail to increase upon oral administration of corn oil [339]. Stimulation of triglyceride breakdown through exogenous supplementation of pancreatic enzymes normalizes lipid-induced GIP secretion in people with cystic fibrosis [339], and, conversely, treatment with the lipase inhibitor orlistat decreases meal-induced GIP secretion in healthy humans [374] and in individuals with T2D [375].

Chylomicrons stimulate GLP-1 secretion from murine intestinal endocrine L-cells derived from colonic tumors (GLUTag cells) and accelerate secretion of GLP-1 and GIP in human and murine duodenal cultures [376]. Inhibition of chylomicron formation using pluronic L-81 inhibits lipid-induced GLP-1 and GIP secretion in mice [377] and rats [378]. Triglyceride re-esterification, and hence chylomicron formation, is impaired in mice deficient for MGAT2 or DGAT1, and these mice show reduced GIP secretion upon oral administration of triglycerides [379]. Thus, lipid-induced GIP secretion is triggered via processes that depend on the physiological breakdown, uptake and intracellular processing of the lipids, overall coupled to the formation of chylomicrons (Figure 4).

The K-cells express a variety of lipid-sensing G-protein coupled receptors, including free fatty acid receptor 1 (FFAR1, a.k.a. GPR40), FFAR4 (a.k.a. GPR120) and GPR119 [95,[380], [381], [382], [383]]. While FFAR1 and FFAR4 are activated by medium and long chain fatty acids [384,385], GPR119 is activated by certain lysophospholipids but mainly by 2-monoacyl glycerides (MAGs), including 2-oleoyl glycerol (2-OG) and esters of palmitic acid or stearic acid [[386], [387], [388]]. The MAGs may also be absorbed without further hydrolysis. The role of the free fatty acid receptors in lipid-induced GIP secretion is somewhat controversial. Mice deficient for either FFAR4 or FFAR1 show a 50–80% reduction in GIP secretion following oral administration of corn- or lard-oil [381,389], and FFAR1 deficient mice show decreased circulating levels of GIP when fed with a HFD [380]. Pharmacological inhibition of FFAR4 in mice attenuates GIP secretion induced by lard-oil [381], and while FFAR1/4 double knock-out (KO) mice show normal glucose-stimulated GIP secretion, lipid-induced GIP secretion is impaired [382]. Although FFAR4 deficient mice show diminished GIP secretion following administration of corn-oil [389], triglyceride-induced GIP secretion is normal in these mice [390]. Oil-triggered GIP secretion in FFAR4-deficient mice is restored by administration of CCK, suggesting that the impaired GIP response may be due to a loss of CCK-dependent gall bladder contraction in this model [389]. When administered acutely together with glucose, the FFAR4 agonist GW-9508 further improves glucose tolerance, increases plasma GIP and enhances glucose-stimulated insulin release in normal but not *Gipr* knock-out (KO) mice [383]. The FFAR4 agonists GSK137647 and Compound A also increase circulating GIP in HFD-fed mice [391]. When fed with a HFD, GPR119-deficient mice show decreased postprandial levels of GLP-1 with unchanged circulating levels of GIP, hence indicating that GPR119 is of minor importance for lipid-induced GIP secretion [392]. Other studies, however, show that lipid-induced GIP secretion is diminished in mice deficient for either FFAR1 or GPR119, but not in mice deficient for FFAR4 [390]. Depending on the ligand, FFAR1 signals through either Gαq and phosphatidylinositol (IP3) pathways, or additionally via also Gαs recruitment and elevation of cAMP [393], although the rise in cAMP downstream of FFAR1 activation has more recently been linked to Gq-dependent adenylate cyclase 2 activation [394]. Gαq selective FFAR1 agonists include α-linolenic acid and docosahexaenoic acid, as well as the synthetic orthosteric agonists TAK-875, AMG-837, MK-2305 and AM-8182, while the ago-allosteric agonists AM-1638 and AM-5262 signal through both Gαq and possibly Gαs recruitment [393]. As demonstrated in mice and murine intestinal primary cell cultures, secretion of GLP-1 and GIP is greater after treatment with super-agonists for FFAR1 such as AM-1638 and AM-5262, which elevate cAMP and Ca^2+^ concentrations, compared with TAK-875 and MK-2305 which predominantly elevate only Ca^2+^ [393]. Interestingly, GPR55 agonists Abd-CBD and AM251 have also been shown to increase plasma GIP in response to glucose in HFD-fed mice [395], with the metabolic effects of Abd-CBD diminished in *Gipr* KO mice [396]. In summary, GPRs play an important role in K-cell function, but the contribution of the individual fatty acid transporters and receptors in lipid-induced GIP secretion varies across studies, and depends on the specific lipid.

The most commonly used cell lines for studies of incretin hormone secretion are GLUTag, STC-1 and NCI–H716 cells [397]. While all of these cell lines show increased GLP-1 secretion in response to administration of glucose [397] or lipids [384], only STC-1 cells show high release and expression of GIP, which can further be enhanced by administration of glucose [397]. Treatment of GLUTag and STC-1 cells with fatty acids increases the cytoplasmic concentration of Ca^2+^ [398,399], and treatment of STC-1 cells with the L-type Ca^2+^ channel inhibitor nicardipine abolishes Ca^2+^ elevation and decreases lipid-induced hormone secretion [398]. In STC-1 cells, knock-down of FFAR4 decreases secretion of GLP-1 induced by α-linolenic acid, while knock-down of FFAR1 has no effect on lipid-induced GLP-1 secretion [384]. The ability of the K-cells to secrete GIP in response to glucose or fat also seems to depend on FAPB5, since mice deficient for FAPB5 show reduced GIP secretion in response to an oral load of glucose or fat [400]. The impaired secretion of GIP in these mice is not related to changes in K-cell number or GIP content [400]. Collectively, the GIP secretory response to orally ingested lipids varies depending on the nature of the lipids, and lipid-induced GIP secretion is likely regulated by mechanisms that include the fat-induced slowing of gastric emptying and stimulation of Ca^2+^ influx via VDC channels.

In conclusion, fat-induced GIP secretion requires the breakdown of triglycerides into monoglycerides and free fatty acids, which is facilitated by pancreatic lipase. In subjects with chronic pancreatitis and reduced exocrine function, this process is impaired. Consequently, such individuals secrete less GIP when fed a triglyceride-rich meal, unless they receive a replacement of pancreatic enzymes (pancreatin) [401,402]. Again, GIP secretion only takes place when the respective substrate triggering secretion is being absorbed.

### 9.6 Neuronal regulation of GIP secretion

Insulin secretion is under control of the autonomic nervous system [403], although cephalic phase secretion is not pronounced in humans [404]. Notably, the pancreatic islets are well innervated by parasympathetic, sympathetic and non-adrenergic, non-cholinergic neurons [405]. In rats, cephalic phase insulin secretion is abolished upon blockade of the parasympathetic nervous system (PNS) by the nicotinic acetylcholine (ACh) receptor inhibitor hexamethonium, or by the muscarinic ACh receptor inhibitor atropine [406]. In healthy humans, cephalic insulin secretion is not related to changes in GIP secretion, but is blocked upon inhibition of sympathetic and parasympathetic neurotransmission by either atropine or trimethaphan [407]. In rats [408] and humans [409], vagal stimulation does not induce GIP secretion, and in healthy humans, modulation of the autonomic nervous system through infusion of phentolamine or atropine during intraduodenal glucose perfusion does not affect GIP secretion [410]. Studies in humans [55] and dogs [411] showed increased basal serum GIP levels after total vagotomy, but postprandial GIP secretion is unchanged in dogs [208,412] or humans [411] upon vagotomy, or after blockade of the cervical vagus [205]. Diminished GIP secretion in response to intrajejunal glucose infusion has nonetheless been reported in some people with truncal vagotomy and pyloroplasty [413], but this has not been confirmed by other studies [163,[414], [415], [416]]. In contrast to the PNS, which do not seem to affect GIP secretion [205,208,[408], [409], [410],^412^], β-adrenergic stimulation of the sympathetic nervous system (SNS), using either terbutaline or isoproterenol, increases plasma GIP levels in dogs [417] and humans [418], and this effect is blocked by treatment with the β-adrenergic receptor antagonist propranolol [418]. Co-infusion of propranolol with epinephrine decreases GIP secretion in response to oral glucose in healthy humans [419], possibly reflecting unshielding of α2-receptor inhibitory action, but other studies report no effect of propranolol on GIP secretion upon intraduodenal infusion of glucose [410]. Direct bolus administration of neuropeptide Y (NPY) [420] or neurotensin [403,421] into the 3rd ventricle of fasted dogs increases plasma levels of GIP and insulin, but given that GIP does not stimulate insulin secretion under conditions of low blood glucose [47,103,200,[422], [423], [424]], the observed elevation of plasma insulin might be independent of the enhanced GIP secretion. Collectively, GIP secretion does not seem to be under control of the PNS, but some studies suggest an involvement of the SNS and the hypothalamus in GIP secretion.

### 9.7 Hormonal regulation of GIP secretion

#### 9.7.1 Regulation of GIP secretion by xenin

In ∽50% of GIP immunoreactive cells, GIP is co-secreted with xenin [425], a 25 amino acid neurotensin-like peptide, which is post-translationally cleaved from the 35 amino acid pro-xenin precursor, derived from the widely-expressed cytoplasmic coatomer protein [426]. In dogs, xenin stimulates secretion of the exocrine pancreas [427,428], inhibits gastric acid secretion induced by pentagastrin [428], and increases plasma levels of insulin and glucagon [428]. Xenin suppresses food intake in rodents [[429], [430], [431], [432], [433]] and delays gastric emptying in humans [434] and mice [435]. Xenin improves glucose metabolism in mice [433,436,437] and healthy humans [438] by potentiating glucose-stimulated insulin secretion, and further by amplifying insulin secretion induced by GIP and GLP-1 [436,438]. Xenin infusion also reverses the reduced insulinotropic action of GIP in patients with impaired glucose tolerance but not those with established T2D [438]. In rat β-cell BRIN-BD11 cells [433] and mouse clonal β-cells [436], xenin enhances insulin secretion under conditions of low and high glucose, but without affecting cAMP levels, membrane potential or Ca^2+^ influx [433]. Other studies report no direct effect of xenin on insulin secretion in isolated murine islets, mouse β-cell MIN6 cells, and the perfused mouse pancreas [437]. Transgenic mice with depletion of GIP-producing K-cells through GIP promoter-driven expression of diphtheria toxin show normal secretion of GLP-1, but abrogated insulin secretion in response to oral glucose [439]. Acute treatment of these mice with either xenin or GIP prior to bolus glucose administration fails to affect glucose tolerance and only minimally increases plasma insulin levels [437]. Yet, the insulinotropic action and improvement of glucose tolerance is restored when xenin and GIP are administered together [437]. Xenin-induced potentiation of insulin secretion is inhibited by blockade of muscarinic but not of nicotinic acetylcholine receptors, indicating that the insulinotropic effect of xenin may be partly mediated by cholinergic neurons that innervate the islets [437]. Interestingly, enzymatically stable analogues of xenin, including a GIP/xenin hybrid have been shown to restore GIP sensitivity, promote the survival and function of pancreatic β-cells and improve glucose homeostasis in DIO mice [440,441]. In summary, the GIP-producing K-cells co-secrete xenin, which synergizes with GIP to enhance glucose metabolism by potentiating insulin secretion. The insulinotropic effect of xenin may involve cholinergic neurons rather than direct β-cell stimulation.

### 9.8 Regulation of GIP secretion by gastrin-releasing peptide (GRP)

As demonstrated in duodenal and jejunal cell preparations of dogs [237], and later confirmed *in vivo* in rats [442,443], dogs [444], and humans [[445], [446], [447]], GIP secretion is induced by gastrin-releasing peptide (GRP), the mammalian homolog of bombesin. In mice, however, GIP secretion is substantially less sensitive to bombesin than GLP-1 release [448], consistent with low expression of the bombesin 2 receptor. GRP is abundantly expressed in neurons and nerve fibers of the proximal gut, the pancreas and the CNS [449,450], and when infused into humans under fasting conditions, it dose-dependently increases the secretion of pancreatic polypeptide, insulin, glucagon, gastrin, CCK and GIP [[445], [446], [447]]. Hormonal secretion induced by bombesin, and hence GRP, is mediated by enhanced Ca^2+^ release from the ER via increased IP3 signaling [451], and is independent of external Ca^2+^ influx, as shown by preserved bombesin-induced Ca^2+^ elevation upon culturing of rat pancreatic acinar cells in Ca^2+^ free medium, or upon blockade of L-type voltage-dependent Ca^2+^ channels using nifedipine [452]. GIP does not increase bombesin-like immunoreactivity in the isolated perfused rat stomach, hence indicating that GIP does not enhance GRP signaling to accelerate its own secretion [453]. In summary, GRP enhances the secretion of GIP by stimulating Ca^2+^ release from the ER through via enhanced IP3 signaling.

### 9.9 Regulation of GIP secretion by somatostatin (Sst)

GIP stimulates somatostatin secretion *in vitro* [352], *ex vivo* [48,[453], [454], [455]] and *in vivo* [[456], [457], [458]], which not only powerfully inhibits its own secretion, but also counteracts GIP-induced insulin secretion. Original observations indicating that somatostatin regulates GIP secretion date back to the work of Pederson and Brown, who in 1975 showed in dogs that i.v. infusion of somatostatin suppresses the rise in plasma IR-GIP following ingestion of glucose or fat in dogs [459]. Accordingly, administration of somatostatin also inhibits the rise in plasma IR-insulin in response to GIP infusion [459], which is in agreement with the role of somatostatin to inhibit secretion of insulin [460,461], glucagon [461], gastrin [462,463], and growth hormone [464].

Supporting its paracrine role in regulating GIP secretion, the somatostatin receptors *Sstr2*, *Sstr3* and *Sstr5* are highly expressed in murine K-cells [352], and somatostatin secreting D-cells are distributed throughout the small intestine [465,466], where they are located in close proximity to the GIP-producing K-cells [467]. Antagonism of SSTR5 in primary small intestinal cultures partially blocks the inhibitory effect of somatostatin on IBMX-induced GIP secretion [352], hence indicating that somatostatin-induced inhibition of GIP secretion is, at least in part, mediated via SSTR5. As demonstrated in rats [456], sheep [457] and humans [458], administration of somatostatin does not affect GIP levels under baseline conditions, but suppresses the secretion of GIP and insulin in response to oral [456,458] or i.v. infused [457] glucose. GIP stimulation of somatostatin secretion has also been demonstrated in the perfused rat pancreas [48], and GIP increases secretion [453] and immunoreactivity [454] of somatostatin in the perfused rat stomach. Therefore, GIP seems to control its own secretion by stimulating the release of somatostatin, which then directly acts on the K-cells to shut-down GIP secretion. In the isolated perfused rat stomach, vagal stimulation [468] or administration of mu-opioid receptor antagonists [469,470] inhibit GIP-stimulated somatostatin secretion. Purified murine K-cells also express the cannabinoid receptor 1 (Cnr1, CB1), and treatment of primary small intestinal cultures with the CB1 agonist methanandamide (mAEA) inhibits GIP secretion induced by IBMX [352]. Treatment of rats with the CB1 antagonist AM251 further increases basal plasma GIP levels, while administration of mAEA prior to oral bolus glucose administration suppresses glucose-stimulated GIP secretion [352]. Collectively, GIP regulates its own secretion by stimulating the release of somatostatin, which acts via the somatostatin receptors on the K-cells to inhibit the release of GIP.

### 9.10 Regulation of GIP secretion by other hormones

Recent transcriptomic and peptidomic analysis of human K-cells showed expression and translation of an array of peptide hormones in addition to GIP [471]. This includes gastrin, CCK, PYY, motilin and somatostatin with expression also detected for numerous GPCRs, such as receptor for somatostatin and secretin, which significantly inhibited and stimulated GIP secretion from human organoids, respectively. Nevertheless, there is relatively little information on the hormonal modulation of GIP secretion in addition to effects of GRP and somatostatin considered above. This may be partly due to fear of complicating indirect effects mediated through interference in nutrient digestion and absorption. However, various studies suggest negative feedback by insulin or proinsulin and C-peptide, but such effects have been reported in some but not all human or rodent studies [105,327,472,473]. Glucagon has also been reported to inhibit fasting- and meal-stimulated GIP release in normal human volunteers [99], although this likely is indirect, as the GCGR, whilst detectable in K-cells, would be expected to stimulate GIP-secretion downstream of Gs-activation. Additionally, negative feedback by the incretin hormones themselves has also been proposed based on observations that the elevated levels of intact/active GIP and GLP-1 induced by DPP4 inhibitors are, paradoxically, associated with reduced overall secretion [357,474]. These data support observations of K-cell function in incretin receptor KO mice [133]; whilst both GIPR and GLP1R mRNA expression was detectable in human organoid derived K-cells, the inhibition is more likely to be indirect, for example through Sst release from D-cells, which also express the incretin receptors [133]. With our updated understanding of targets for GIP action, further studies are required to examine whether hormonal K-cell modulation is also derived from adipose tissue (leptin, other adipokines), bone (osteocalcin, PTH), gonads (brain-reproductive axis) and hypothalamus (via ANS as considered above).

### 9.11 Secretion of GIP under conditions of hyperglycemia, diabetes and obesity

Studies related to secretion of GIP under conditions of impaired glucose metabolism are conflicting. While some studies show exaggerated glucose-stimulated GIP secretion in people living with T2D [26,[475], [476], [477], [478], [479], [480], [481]], this is not confirmed in other reports [151,193,312,314,[482], [483], [484], [485]], including a large-scale meta-analysis comprising 22 studies [141]. A greater rise in IR-GIP following ingestion of a test meal was also observed in people with chronic pancreatitis [486], which led to the hypothesis that GIP hypersecretion may originate from the loss of a pancreatic feedback loop that normally inhibits GIP secretion under conditions of hyperinsulinemia. Indeed, GIP responses to ingestion of triglycerides are diminished in healthy humans upon i.v. infusion of insulin [26]. However, while several studies support the notion that hyperinsulinemia attenuates the rise in plasma GIP following oral ingestion of glucose [101,102], this is not supported by other studies [[103], [104], [105]]. Furthermore, while infusion of insulin alone, or together with glucose, suppresses the rise in serum IR-GIP induced by oral administration of fat in people with T1D, no such effect is observed after oral ingestion of glucose [105]. Studies in mice made insulin-deficient through administration of STZ show elevated plasma levels of GIP in the non-fasted state and following an oral load of fat, reflecting hyperphagia and possibly a lack of feedback inhibition by insulin [472,487]. However, in obese-hyperglycemic ob/ob mice, which exhibit remarkably elevated GIP concentrations, even very high doses of exogenous insulin failed to suppress basal, fat- or glucose-stimulated GIP release [327]. Data regarding the impact of T1D are equally conflicting, with normal [105,303,314,483,[488], [489], [490]], or elevated [491] levels of GIP in the fasted state, and normal [105,483,488,489,492], elevated [493] or decreased [303,494] levels postprandially being reported. However, while the use of different assays with widely varying specificities for GIP quantification might have contributed to the observed variation across the different studies, the majority of data indicate that postprandial levels of GIP are not overtly different between healthy subjects and individuals with T1D or T2D. Interestingly, at least 20% of GIP extracted from the intestines of ob/ob mice is N-terminally glycated and therefore resistant to DPP4 [495]. This suggests that GIP in secretory granules is normally glycated during storage in the highly glucose rich environment of intestinal K-cells and partially protected from degradation when it is released into the blood.

While individuals living with T2D show normal fasting and postprandial levels of GIP, a high BMI is associated with increased GIP secretion following ingestion of glucose or a mixed meal [141]. Indeed, meal-induced GIP secretion correlates positively with the amount of visceral fat and the waist-to-hip ratio [496]. Exaggerated nutrient-stimulated GIP secretion is also observed in obese leptin-deficient ob/ob mice [327,328,340] and in humans with obesity [481,[497], [498], [499], [500]], but this has not been confirmed by other studies [492,[501], [502], [503], [504]]. In individuals with obesity, GIP hypersecretion in response to glucose or a mixed meal is unrelated to hyperinsulinemia and is only observed after ingestion of a high calorie meal, potentially as consequence of an increased rate of gastric emptying [498]. In people living with obesity and T2D, plasma levels of intact GIP after breakfast, lunch and dinner are, as expected, increased in individuals receiving DPP4 inhibitors (saxagliptin, sitagliptin or vildagliptin) relative to those receiving placebo, but this does not reflect increased secretion [505]. Collectively, while the findings presented indicate that postprandial levels of GIP are not overtly different between people with or without T2D, plasma levels of GIP are elevated in states associated with excess adiposity.

### 9.12 Secretion of GIP under conditions of gastric bypass surgery

Elevated GIP in obesity, and related observations of GIP effects on adipose tissue, led Vincent Marks in 1988 to describe GIP as ‘The Obesity Hormone’ [506]. There is also some evidence that diminished GIP secretion may be a feature of certain forms of bypass surgery associated with weight loss and remission of T2D [507,508]. However, in contrast to GLP-1, data related to circulating levels of GIP after gastric bypass are conflicting, with postprandial GIP levels being elevated [[509], [510], [511]], unchanged [512,513] or decreased [[514], [515], [516], [517], [518], [519]]. Possible explanations for the overall rather minor and conflicting effects of gastric bypass on circulating GIP levels may be the retention of functional capacity by duodenal K-cells located in the alimentary limb [520], and further potential differences in sampling time, meal composition, lengths of the intestinal limb and the assay used to measure GIP. Nonetheless, separate and combined antagonization of GLP-1R and GIPR during a mixed meal showed that the relative importance of GIP and GLP-1 for postprandial glucose control shifts after gastric bypass, with GIP being the more important incretin in unoperated individuals, but with GLP-1 becoming the dominant incretin after sleeve gastrectomy or RYGB [514]. The observation that postprandial levels of GIP are reduced in individuals who underwent RYGB relative to adjusted gastric banding (AGB) have also let to the hypothesis that the lower GIP levels may contribute to the greater weight loss that is typically observed after RYGB relative to AGB [517]. But no clinical study has yet assessed the effect of GIPR antagonism on weight loss after AGB. Collectively, the effect of gastric surgery on GIP levels remains controversial. While GLP-1 predominates in postprandial glucose control after bypass surgery, the role of GIP may be diminished, with lower GIP levels potentially contributing to greater weight loss after RYGB compared to AGB.

### 9.13 Secretion and action of GIP in pancreatitis and cystic fibrosis related diabetes

Although less well researched, there is evidence for significant involvement of GIP in other physiological and pathophysiological conditions. Postprandial GIP secretion is impaired in chronic pancreatitis and cystic fibrosis-related diabetes (CFRD), and as might be expected improved following pancreatic enzyme substitution [521,522]. The aberrant secretion of GIP in CFRD is accompanied by failure of islet β-cells to respond to GIP, but not to GLP-1 infusions [523], illustrating subtle differences in the insulin secretory pathways of the two incretins resulting from disturbances in the cystic fibrosis transmembrane conductance regulator (CFTR) on β-cells.

### 9.14 Secretion and action of GIP in gestational diabetes, pregnancy, lactation and cold exposure

Data related to postprandial GIP levels under conditions of gestational diabetes (GD) are conflicting, with GIP responses to oral glucose or fat being mildly reduced [524,525] or increased [526]. Nonetheless, current consensus is that neither pregnancy nor GD have clinically relevant effects on GIP responses [527,528]. More detailed studies of GIP during pregnancy/lactation have been conducted in rats [529]. In pregnancy, no significant changes in circulating GIP are observed apart from a modest decrease in response to oral glucose. Along with significant islet expansion, GIP was co-expressed with glucagon in *α*-cells, but GIP is not a player in the adaptive responses to pregnancy as confirmed by studies in *Gipr* KO mice [113]. In lactation, the GIP response to oral glucose was unchanged, but unlike pregnancy GIP concentrations were markedly raised in association with substantial hyperphagia and intestinal hypertrophy. These changes were not key in the accompanying modest improvement of glucose tolerance but they were associated with prominent increases in mammary gland expression of *Gipr* and genes involved in energy turnover, free fatty acid and glucose uptake, thereby promoting enhanced triglyceride formation [529]. Interestingly, this study also revealed exceptionally high plasma GIP concentrations in the pups throughout lactation, also suggesting possible involvement of GIP in lipid metabolism during development.

Further studies have been performed to evaluate changes in GIP during cold acclimation in rats, a situation associated with hyperphagia, increased metabolic demand and adaptive, non-shivering, thermogenesis [530]. Animals housed under normal husbandry conditions, but at 4 °C, exhibited normal body weights, improved glucose tolerance and insulin sensitivity and raised circulating GIP accompanied by exaggerated GIP response to oral glucose. Despite the persistence of hyperphagia, these effects on GIP were observed up to 24 days but by day 42 when BAT mass was doubled, only GIP intestinal stores were increased. GIP may therefore play a role initially in adaptive energy metabolism to cold exposure, but other mechanisms most likely triggered by chronic sympathetic activation are important in the longer term. Summarizing these data, levels of GIP change only minimally during pregnancy and gestational diabetes, and GIP does not seem to play a major role in the metabolic adaptation to pregnancy or lactation.

### 9.15 Secretion and action of GIP in food-dependent Cushing's syndrome, other neuroendocrine tumors and insulinoma

ACTH-independent Cushing's syndrome is due to the development of ectopic GIPR expression in the adrenal cortex, which arises secondary to germline or somatic loss of *KDM1A* expression [531] and gives rise to the secretion of cortisol upon release of GIP from the gut [532,533]. This results in characteristic features of Cushing's syndrome and, as noted by Vincent Marks [62], this discovery was the first evidence of a causative role for GIP in human disease. Disturbances relating to GIP are also encountered in cases of insulinoma, including the production of GIP and expression of GIP receptors [[534], [535], [536]]. Another example is the expression of the GIPR in somatotropinomas (growth hormone-secreting pituitary adenomas). Due to this expression, GIP-secretion has been linked to paradoxical increases in GH during an OGTT in approximately 30% of patients with acromegaly [537]. A recent study described that the antagonist GIP(3-30)NH2, when administered during an OGTT in patients with acromegaly, inhibited the paradoxical GH secretion in some patients, suggesting that targeting the GIP/GIPR axis could be a potential therapeutic approach for managing acromegaly in patients with GIPR-expressing pituitary adenomas [253]. Indeed, GIPRs are found on many neuroendocrine tumors, offering the possibility of exploitation for tumor imaging, diagnosis and treatment using radioligand of GIP, PET analysis and GIPR antagonists [253,538]. Human insulinomas generally exhibit markedly disturbed stimulus-secretion coupling pathways [539], explaining why the insulinotropic effect of GIP is almost abolished in many insulinoma patients [540].

## 10 Identification and characterization of the GIP receptor (GIPR)

The rat GIP receptor (rGIPR) was cloned in 1993 from cDNA libraries of rat cerebral cortex and rat insulinoma RINm5F cells [541] (Figure 1), characterizing GIPR as a seven-transmembrane G protein-coupled receptor (GPCR) of the class B (a.k.a. secretin receptor) family. Two years later, the human GIP receptor (hGIPR) was identified by decoding cDNA clones from pancreatic islets [542] and an insulinoma [543] (Figure 1). Like all other members of the secretin receptor family, GIPR is a Gα_s_-coupled receptor that amplifies cAMP production through activation of adenylate cyclase [[541], [542], [543], [544], [545]]. Members of the secretin receptor family are mostly activated by a single endogenous ligand, after which the receptor is historically named (e.g. GIPR, GLP-1R, GLP-2R, etc.) [544,546], but some promiscuity is often found. For example, zebrafish GIP promotes insulin secretion and/or cAMP production also via the human and rat GLP-1R [547]. Furthermore, GIP, GLP-1 and glucagon all have similar affinity and potency to promote cAMP production via their cognate receptors, GIPR, GLP-1R and glucagon receptor (GCGR), respectively [548,549]. However, glucagon, in addition to activating the glucagon receptor (GCGR), also activates GLP-1R in pancreatic β-cells to stimulate insulin secretion, albeit with lower affinity and potency than GLP-1 [[550], [551], [552]]. In line with the notion that GIP is the most important endogenous ligand for GIPR and robustly promotes cAMP reporter activity in LGIPR2 cells transfected to express *Gipr*, while no such effect is observed after treatment with GLP-1, GLP-2, glucagon, VIP, GHRH, gastrin, calcitonin, or GRP [541].

Notably, GIPR differs markedly from GLP-1R in intracellular signaling and trafficking. While both are coupled to the Gs pathway, GLP-1R is in parallel also coupled to Gq signaling, an attribute potentially contributing its preserved insulinotropic effectiveness under conditions of diabetes [553]. Additionally, ligand-induced recruitment of Gs to its receptor is lower for GIPR relative to GLP-1R, despite near preserved ability for cAMP production, suggesting that GIPR more efficiently engages in signal amplification within the Gs-cAMP pathway [554]. Despite differences in Gs recruitment, both GIPR and GLP-1R activation result in nearly equal amounts of GTP-loaded active Gs at the plasma membrane, while GIPR exhibits less active Gs in endosomal compartments, indicating a preferential signaling hub at the plasma membrane for GIPR [554]. This may be associated with the GIPR internalizing to a lesser degree in both total volume and rate relative to GLP-1R, which is reflected by relatively minimal preceding ligand-stimulated GIPR clustering within the plasma membrane [[554], [555], [556]]. Canonical GPCR internalization and plasma membrane aggregation is typically facilitated via arrestin recruitment. However, while the GLP-1R acts atypically in its arrestin-independent internalization, evidence suggests that arrestins mediate at least some degree of ligand-stimulated internalization of the GIPR [[555], [556], [557], [558], [559]]. The GIPR exhibits ligand-free constitutive receptor internalization and rapid recycling back to the plasma membrane, both of which are arrestin-independent and not observed with the GLP-1R [556,557]. However, the disappearance of the GIPR from the plasma membrane upon ligand stimulation does not appear to be due to a ligand-induced increase in internalization rate but rather an inhibition of receptor recycling back to the plasma membrane, a process that depends on arrestins [556,557]. Supporting the significance of arrestins for overall GIPR function, a recent study of over 40 naturally occurring GIPR variants established arrestin recruitment as a central property for maintaining GIPR function in variants with loss-of-function in G protein activation [560]. However, whether arrestins directly interact with the GIPR or act as scaffolding remains unclear. Both GLP-1R and GIPR signaling are negatively modulated by arrestins [555,561], with the GLP-1R exhibiting robust colocalization with arrestins in both direct complementation and proximity-based assays [549,554]. In contrast, the GIPR presents a more enigmatic profile, showing evidence of direct interaction with arrestins only under synthetically optimized conditions designed to enhance arrestin signals (e.g., the use of helper peptides or stable cumulative luminescent products) [549,560,562]. However, in real-time complementation or proximity-based assays measuring direct native interactions without synthetic enhancements, GIPR-arrestin interactions are not robustly detected [554]. Additionally, non-trivial influences of linker composition and co-overexpression of GPCR kinase 2 (GRK2) on relevant arrestin interactions has complicated the understanding behind the mechanistic role of arrestins with GIPR [563].

Among the receptors of the secretin receptor family, GIPR shows highest homology (∼40%) to the receptors for glucagon and GLP-1 [542]. However, GIP and its receptor are evolutionarily less conserved than the GLP-1 system. Thus, while the GLP-1 peptide sequence is 100% conserved between humans, rats, and mice, GIP shows only 93–97% sequence identity between these species [564,565]. And while human (h)GLP-1R shows 92% and 93% sequence homology to rat and mouse GLP-1R [564], hGIPR is only 81% homologous to the rat and mouse GIPR [543,564]. These species-specific differences translate into notable pharmacodynamic differences. While rat and mouse GIP are more potent than hGIP to promote cAMP production via the human, mouse and rat GIPR, hGIP does not maximally induce cAMP production via the rat and mouse GIPR, even at high concentrations [564]. Furthermore, although human and rodent GIPR exhibit similar potencies for Gαs recruitment after stimulation with their cognate ligands, the rodent GIPR, and in particular mGIPR, shows reduced receptor desensitization, internalization and β-arrestin recruitment relative to hGIPR [562].

Activation of GIPR by its ligand is assumed to be initiated by the interaction of the alpha-helical part of the GIP middle and C-terminus with the N-terminal extracellular domain (ECD) of the receptor, which optimally positions the GIP N-terminus for engagement with the receptor core, where it forms extensive polar and non-polar interactions that are crucial for receptor activation [[566], [567], [568], [569]]. Key residues within the core that for GIP-interaction that leads to receptor activation include Y141^1.43^, R183^2.60^, R190^2.67^, Q224^3.37^ R300^5.40^, R370^7.35^, L374^7.39^. Moreover, GIPR antagonists are reported to inhibit receptor activation via putative disruption of the K293^5.33/ECL2^-E362^ECL3^ salt bridge that is observed in GIP-bound GIPR cryo-EM structures [570]. Emphasizing the importance of the two-step GIP binding process for receptor activity, exchanging the GIP C-terminus between human, rat and mouse GIP extinguishes the ability of such C-terminally modified hGIP to recruit β-arrestin, but without changing Gαs signaling [562]. Nonetheless peptide structure–activity studies using synthetically generated GIP fragments in the perfused rat pancreas [571], monkey kidney COS-7 cells [572] and Chinese hamster ovary (CHO) cells transfected to express *Gipr* [228,573] demonstrate the crucial role of the GIP N-terminus for binding, receptor activation and signalling. While human and porcine GIP(1-30) are nearly as high affine and potent as hGIP(1-42) in GIPR binding and cAMP production, respectively [228,543,574], and GIP(1-30)NH2 has similar effects on glucose and bone metabolism in healthy individuals as GIP(1-42) [230], GIP(19-30) is largely without effect [228]. Although GIP(1-30) improves glucose tolerance with equal efficacy to GIP(1-42) in rats, 10-fold higher doses of GIP(19-30) are required to affect glucose clearance [228]. In GIPR expressing CHO cells, GIP(6-30) binds to GIPR with comparable affinity to GIP(1-42), but competitively inhibits cAMP production induced by GIP(1-42), hence identifying GIP(6-30) as the first GIPR antagonist [575]. A variety of N-terminally truncated GIP fragments were subsequently identified to inhibit GIP(1-42)-induced insulin secretion in rat β-cell BIN-BD11 cells, including human (h) and murine (m) GIP(3-30) and hPro(3)GIP(3-30), but not hGIP(5-30), hGIP(5-42) or hGIP(3-42) [576,577]. hPro(3)GIP is a weak partial agonist at the rodent GIPR and a partial to full agonist at hGIPR, dependent on the level of receptor expression [564]. Therefore in systems with low receptor reserve, such as *in viv*o in rodent models, hPro(3)GIP can behave as a competitive antagonist of GIP(1-42)-induced responses. Notably, while hGIP(3-30)NH2 also inhibits GIP(1-42)-induced cAMP production and β-arrestin recruitment at hGIPR [574], and shows GIPR selectivity across 62 human GPCRs, its antagonistic activity is species-specific, with high affinity for hGIPR, but with only relatively low affinity at the mouse and rat GIP receptor [559]. In line with this notion, hGIP(3-30)NH2 blocks GIP-induced insulin secretion in humans [578]. In summary, GIPR is a primarily Gαs-coupled GPCR, which differs from its related receptors in weaker arrestin recruitment and reduced receptor internalization. Ligand-induced activation of GIPR involves a two-step process, where the interaction of the GIP N-terminus with the ECD of GIPR is crucial for receptor activation.

### 10.1 Peripheral distribution of the GIP receptor

Due to availability of only insufficiently selective GIPR antibodies, studies to detect *Gipr*/GIPR relied on radioligand-binding studies, *in situ* hybridization, qPCR, and RNA-sequencing [579]. More recently, fluorescently labeled ligands have been employed to detect GIPR in the CNS [580,581]. High-affinity binding of radiolabeled GIP has been shown in rat insulinoma RINm5F cells [545], hamster pancreatic β-cells [582], and membrane preparations of hamster β-cell tumors [583]. In adult humans, expression of *GIPR* is high in the pancreas and the trachea, moderate in the heart, gut, spleen, blood cells, thymus, lung, kidney and bones, and absent in the liver, placenta, testis, uterus, and the adrenals [584,585]. In human fetal tissue, *GIPR* expression is found in the lung, heart, and kidney, but not in the brain, liver, spleen and thymus [584]. Using *in situ* hybridization, expression of *Gipr* is found in the rat pancreas, gut, adipose tissue, heart, pituitary, the vasculature, the inner layers of the adrenal cortex, and several regions of the brain, including the cerebral cortex, hippocampus, and the olfactory bulb, with no expression in the kidney, spleen, and liver [541]. Radioligand-binding studies show GIP binding in the rat cerebral cortex, anterior olfactory nucleus, lateral septal nucleus, subiculum, inferior colliculus, and inferior olive [586]. Mice that express Cre-dependent enhanced yellow fluorescent protein (EYFP) under control of the *Gipr* promoter show EYFP fluorescence in pancreatic *α*-, β- and δ-cells, the exocrine pancreas, the pituitary, ovary, uterus as well as a subset of adipocytes of the interscapular brown adipose tissue (iBAT) and the inguinal white adipose tissue (iWAT) [587,588]; signals in adipocytes were subsequently considered a lineage tracing result in which the Cre-reporter was activated at the preadipocyte level [589]. In the embryonic mouse pancreas, ∽95% of the *Gipr* positive cells correspond to the endocrine pancreas, with only limited expression of *Gipr* in the exocrine pancreas (∽1%), blood vessels (∽2.8%), immune cells (∽0.4%) and mesenchymal cells (0.8%) [590,591]. In agreement with scRNA-seq data showing that *Gipr* is abundantly expressed in the *α*- and β-cells [590], qPCR analyses in mice show high expression of *Gipr* in pancreatic islets, but low expression in BAT and the adrenals [592]. In the white adipose tissue (WAT), scRNAseq analysis show *Gipr* expression in pericytes, mesothelial cells and a subpopulation of adipocytes [589,593]. Expression of *GIPR* is also found in cultured human adipocytes [559,594,595] and 3T3-L1 cells [594,596,597], hence under conditions where no stromal-vascular fraction is present. *Gipr* is further expressed and functionally active in murine and human white adipocytes [598], which is in agreement with the observation that GIPR agonism autonomously promotes either storage or utilization of lipids in 3T3L1 adipocytes [594,[599], [600], [601]] and in adipocytes isolated from humans [600,602,603], rats [594,604,605] and mice [603].

### 10.2 Central nervous system distribution of the GIP receptor

Soon after the identification of *Gipr* [541], central nervous system expression of *Gipr* was shown using Northern blot [541], *in-situ* hybridization [541], radiolabeled GIP binding studies [586], immunohistochemistry [121] and qPCR analysis [541,606]. Collectively, these data show widespread expression of *Gipr* throughout the rodent brain, including the olfactory bulb, telencephalon, diencephalon, hypothalamus, brainstem and cerebellum [121,541,586,606]. More recently, central *Gipr* expression was confirmed using scRNAseq [587,[607], [608], [609]] and RNAscope [587]. Transcriptional activity of the *Gipr* gene was also studied using mice that express Cre-dependent yellow fluorescent protein (YFP) under control of the *Gipr* promoter [587,609]. Such mice show widespread YFP immunostaining throughout the CNS, including the hypothalamic arcuate nucleus (ARC), paraventricular (PVH) and dorsomedial hypothalamic nuclei (DMH), and the area postrema (AP) of the hindbrain [587]. As demonstrated using scRNAseq analysis in the hypothalamus [587,591,608,609] and hindbrain [607,610,611], *Gipr* is robustly expressed in mural cells, but with substantial populations also of oligodendrocytes and neurons. In the hypothalamus and hindbrain, *Gipr* expressing neurons are primarily GABAergic, with only minor expression of *Gipr* in glutamatergic neurons [587,[607], [608], [609], [610], [611]]. In line with the observation that GLP-1R neurons implicated in food intake control are primarily glutamatergic [612], very few neurons in the hypothalamus or the hindbrain express both incretin receptors [587,591,[607], [608], [609], [610], [611]]. In the rodent hypothalamus, *Gipr* is expressed in only 1–1.5% of cells that express the leptin receptor [591], and while ∽80% of *Gipr* positive neurons in the hypothalamus co-express *Sst* [587], only a fraction (<1%) of POMC and AgRP neurons express *Gipr* [587,591,608]. These data nonetheless have to be regarded with caution, since regional and spatiotemporal expression of *Gipr/GIPR* may vary across species, and has yet not been thoroughly assessed in humans beyond the demonstration that *GIPR* is expressed in the human hypothalamus using RNA scope [587] and sc/snRNAseq [608]. Furthermore, single cell (sc) and single nucleus (sn)-RNAseq carries the limitation that transcripts expressed at low levels (such as *Gipr*) might be underrepresented (or remain undetected) during RNA library preparation. In addition, the use of Cre-reporter strains picks up cells that expressed *Gipr* earlier in their lineage, in addition to those actively expressing *Gipr*. In any case, *Gipr* is widely expressed in the rodent CNS, including the hypothalamus and the brainstem, and with predominant expression in GABAergic neurons.

### 10.3 Regulation of Gipr expression

The regulation of *Gipr* expression is under control of the peroxisome proliferator-activated receptor gamma (PPAR*γ*), which binds to a PPAR response element in the *Gipr* promoter to enhance *Gipr* expression [613]. Expression of *Gipr* is reduced by 70% in pancreatic islets of PPAR*γ* deficient mice and is 2-fold upregulated in 60% pancreatectomized rats overexpressing PPAR*γ* [613]. Pharmacological activation of PPAR*γ* using thiazolidinediones (TZDs) increases GIPR levels and accelerates glucose-stimulated insulin secretion in isolated mouse islets [613]. Despite discrepant results related to the expression of *Gipr* in adipocytes *in vivo* vs. *ex vivo* [589], mRNA or protein level of GIPR have been reported to increase during adipocyte differentiation in human [594,595,598] and 3T3-L1 preadipocytes *ex vivo* [594,596], and this is associated with increased activation (acylation) of histones H3/H4, and enhanced expression of PPAR*γ*, which all bind to a PPAR response element in the *Gipr* promoter to induce its expression [596]. In VDF rats, WAT expression of *Ppparγ* is closely paralleled by expression of *Gipr*, with high expression in the epididymal and retro-peritoneal WAT, moderate expression in mesenteric WAT and low expression in the interscapular BAT and the perirenal, inguinal and intracardial WAT [614]. Treatment of differentiated 3T3-L1 adipocytes with the PPAR*γ* agonists LY171883 or rosiglitazone increases *Gipr* expression, while treatment with the PPAR*γ* antagonist GW9662 has the opposite effect [596]. Additionally, treatment of obese mice with rosiglitazone increases the expression of the *Gipr* in the white and brown adipose tissue [615]. Expression of *Gipr* further decreases in INS(832/13) cells under conditions of high glucose [167]. Moreover, while *Gipr* expression increases upon treatment of INS(832/13) cells with either palmitic acid or the PPAR*α*-selective agonist WY 14643 under conditions of low but not high glucose, pharmacological or genetic blockade of PPAR*α* decreases *Gipr* expression [167]. Overall, these data indicate that lipids may promote islet *Gipr* expression via PPAR*α* in a glucose-dependent manner, with stimulation of *Gipr* expression under normoglycemic conditions, and suppression of *Gipr* expression under conditions of hyperglycemia [167]. In line with this notion, diabetic VDF rats show blunted insulin secretion after treatment with GIP, along with reduced expression of *Gipr* in the pancreatic islets [164,165], and restoration of normoglycemia in VDF rats increases pancreatic expression of *Gipr* and improves GIP responsiveness to improve glucose tolerance [165]. *GIPR* expression levels in response to diabetes, impaired glucose homeostasis or metabolic derangements are not known in humans.

## 11 GIP regulation of pancreatic hormone secretion

### 11.1 Regulation of insulin secretion

**Figure 5:**
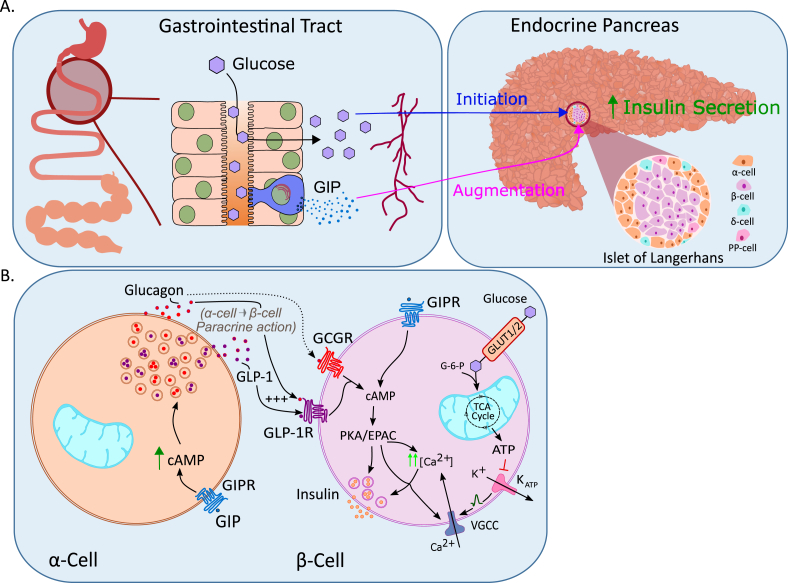
**Incretin-mediated regulation of pancreatic function.** (A) Postprandial insulin release is initiated by direct sensation of circulating glucose in pancreatic islet cells. Dietary glucose triggers GIP secretion from intestinal K-cells, which augments insulin release from pancreatic beta cells both directly and indirectly. (B) Glucose uptake into pancreatic beta cells is mediated by GLUT1 (human) of GLUT2 (rodent) transporters. Glucose is then metabolised via glycolysis and the tricarboxylic acid (TCA) cycle into ATP. The subsequent increase of intracellular ATP/ADP closes K_ATP_ channels, leading to membrane depolarisation and the opening of voltage-gated calcium channels (VGCC). Increased intracellular calcium triggers insulin granule exocytosis. GIP directly augments insulin release through binding GIPR expressed on the surface of beta cells. GIPR engagement increases cAMP levels through the recruitment of Gαs, leading to increased PKA/EPAC activity. Increased PKA/EPAC signalling enhances Ca^2+^ influx through VGCCs and primes insulin granules for Ca^2+^-dependent exocytosis. GIP indirectly increases insulin release through stimulating alpha cell activity and promoting alpha → beta cell paracrine regulation. Pancreatic alpha cells produce GLP-1 in addition to glucagon through alternative processing of the proglucagon precursor. The release of both glucagon and GLP-1 from alpha cells is thought to augment glucose stimulated insulin release through GLP-1R- and (to a lesser extent) GCGR-mediated increases in cAMP. cAMP, cyclic adenosine monophosphate; EPAC, exchange protein directly activated by cAMP; G-6-P, glucose-6-phosphatase; GPCR, G-protein-coupled receptor; GGCR, glucagon receptor; PKA, protein kinase A; TCA, tricarboxylic acid; VGCC, voltage-gated calcium channel.

The meal-induced increase of plasma GIP is closely paralleled by the rise of plasma insulin [304] (Figure 5). However, it was already noted in 1975 that the ability of GIP to enhance insulin secretion greatly depends on the levels of blood glucose [47,200], an observation that also applies for the insulin secretory response to GLP-1 [616]. Although ingestion of fat leads to a greater rise in serum GIP relative to that achieved with glucose [26,198,200,300], fat-stimulated GIP secretion does not stimulate substantial insulin secretion [203], and the rise in circulating GIP is only paralleled by enhanced insulin secretion after ingestion of glucose, but not after sole ingestion of fat [[198], [199], [200],^424^] or proteins [198]. Under normoglycemic conditions, insulin does not inhibit fat-stimulated GIP secretion [424], and while exogenous administration of porcine GIP in dogs fails to stimulate insulin secretion under baseline conditions, it rapidly increases insulin levels when co-infused with glucose [200]. As demonstrated in isolated rat islets [47], and later confirmed in human studies [103,[422], [423], [424]], GIP has negligible effects on insulin secretion under conditions of hypoglycemia, but strongly potentiates insulin secretion when circulating glucose levels reach or exceed 6–8 mmol/l [158,422]. Meal-stimulated levels of GIP also stimulate insulin secretion at fasting glucose levels (provided these are maintained by clamping) [158].

Although mechanistic studies on the insulinotropic action of the incretin hormones are largely based on GLP-1, there is reasonable evidence indicating that GIP promotes insulin secretion through shared and complementary mechanisms (Figure 5). In hamster β-cell HIT-T15 cells, insulin secretion is reduced after repeated episodes of short-term perifusion with GLP-1 or GIP, suggesting that both incretins rapidly desensitize their cognate receptors [616]. For example, GLP-1 and exendin-4 acutely desensitize GLP-1R-dependent cAMP production in rat insulinoma INS-1 cells [617]. However, pretreatment with GIP does not affect insulin secretion induced by GLP-1, and pretreatment with GLP-1 does not affect the insulin secretory response to GIP [616]. Furthermore, repeated episodes of GLP-1 infusion over the course of 6 h does not lead to alterations in insulin secretion in healthy humans clamped at pre- and postprandial plasma glucose concentrations [158]. GLP-1 and GIP further additively enhance insulin secretion in healthy humans [155] and in the isolated perfused rat pancreas [618,619] (Figure 5). Mice with concomitant deletion of GLP-1R and GIPR show a greater impairment of oral glucose-stimulated insulin secretion relative to mice that lack only one of the incretin receptors [133,620,621], and similar results have been observed in healthy humans upon pharmacological blockade of either GLP-1R or GIPR [131]. Collectively, these data indicate that the incretins affect insulin secretion via shared and complementary mechanisms [553].

Glucose enters the β-cells via specific glucose transporters (GLUT), and while GLUT1 and GLUT2 are expressed in rodent β-cells [622], there is very little GLUT2 in the majority of human β-cells [623,624]. Following glucose entry, glucose metabolism generates ATP, and the resulting increase in the ATP/ADP ratio leads to closure of ATP-sensitive potassium (K_ATP_) channels, depolarization of the cell membrane and opening of voltage-dependent Ca^2+^ (VDC) channels [[625], [626], [627]] (Figure 5). The subsequent Ca^2+^ influx triggers vesicular exocytosis and release of insulin into the circulation [625,626]. The K_ATP_ channels comprise four pore-forming Kir6.2 subunits and four regulatory SUR1 subunits. In SUR1 deficient murine islets, GLP-1 and GIP normally elevate cAMP, but fail to potentiate glucose-stimulated insulin secretion [628,629]. In Kir6.2 deficient mice, however, GLP-1, but not GIP enhances glucose clearance following bolus glucose administration, seemingly suggesting that functional K_ATP_ channels play a greater role for the insulinotropic effect of GIP relative to GLP-1 [56]. However, studies in isolated rat islets [630] and mouse pancreatic β-cells [631] show that GLP-1 inhibits K_ATP_ channels to promote Ca^2+^ influx via membrane depolarization and opening of VDC channels. In agreement, GLP-1 and GIP both promote Ca^2+^ influx into the β-cells under conditions of high glucose [632,633], and cause membrane depolarization by decreasing whole-cell K_ATP_ conductance in isolated human β-cells [634] (Figure 5). In addition, Ca^2+^ influx induced by GLP-1 and GIP is blocked by inhibition of L-type VDC channels, or when the cells are kept under conditions of low extracellular Ca^2+^ [632,635]. Both incretins hence promote insulin secretion by modulating K_ATP_ channel activity and by accelerating Ca^2+^ entry via VDC channels (Figure 5).

The ability of the incretins to promote cAMP production has been demonstrated in various cell lines, including rat insulinoma RIN1046-38 cells [78], isolated rat islets [48,636], hamster pancreatic In111 cells [637], hamster β-cell HIT-T15 cells [616], and mouse insulinoma βTC-3 cells [168]. At submaximal binding concentrations, GLP-1 and GIP additively enhance cAMP production [545], and elevation of cAMP using either forskolin [[638], [639], [640], [641], [642]] or IBMX [636,643] increases islet insulin secretion under conditions of high glucose. Although cAMP production induced by either GIP or forskolin is independent of the cultured glucose condition [168,638,641,642], cAMP promotes insulin secretion only in the presence of high glucose [638,641,642]. These data indicate that cAMP alone is not able to induce membrane depolarization, and hence to accelerate Ca^2+^ influx via opening of VDC channels, but that cAMP potentiates insulin secretion under conditions where the VDC channels are open. In keeping with this notion, under conditions of high glucose, elevation of cAMP using either forskolin or db-cAMP increases Ca^2+^ influx in rat pancreatic β-cells similar to treatment with GLP-1(7-36)NH2, and this effect is blocked by inhibition of L-type VDC channels using nitrendipine [635]. In the pancreatic β-cells, cAMP is rapidly degraded by the cyclic 3′, 5′-nucleotide phosphodiesterase 3B (PDE3B), and overexpression of PDE3B in rodent islets or rat insulinoma INS-1 cells decreases insulin secretion in response to glucose and GLP-1 [644,645]. Consistent with this finding, the opposite response, an increase in cAMP-mediated exocytosis and accelerated glucose-stimulated insulin secretion, is seen with inhibition of PDE3B [644]. In summary, while the generation of cAMP does not depend on extracellular Ca^2+^ influx, and under low glucose conditions is not sufficient to stimulate insulin secretion, it potentiates glucose-stimulated insulin release when ambient glucose is elevated above fasting concentrations.

### 11.2 PKA-dependent effects of cAMP on insulin secretion

Elevation of cAMP leads to activation of protein kinase A (PKA) [646,647], which affects the insulin secretory machinery via multiple mechanisms. PKA is a holoenzyme, comprising two catalytical and two regulatory subunits, which dissociate in a cAMP-dependent manner to become active [648,649]. Upon activation, PKA decreases K_ATP_ channel activity through phosphorylation of the Kir6.2 and SUR1 subunits [650,651], and treatment of isolated mouse pancreatic β-cells with the selective PKA inhibitor Rp-cAMP blocks GLP-1 inhibition of K_ATP_ channel activity [631]. PKA further activates L-type VDC channels through phosphorylation of their *α*_1_1.2 and β_2a_ subunits [[652], [653], [654], [655], [656]]. As shown in isolated rodent β-cells, this cAMP/PKA-mediated activation of VDC channels increases Ca^2+^ influx and accelerates exocytosis of the insulin granules [657], while inhibition of PKA using Rp-cAMP prevents the stimulatory effect of cAMP on L-type VDC channel activity [658]. At the same time, the activated PKA inhibits voltage-dependent K^+^ (K_v_)-channels, which delays membrane repolarization and thus increases Ca^2+^ influx via prolonged opening of the VDC channels [659] (Figure 5). Emphasizing its role in insulin secretion, inhibition of PKA in rat insulinoma RIN 1046-38 cells attenuates insulin secretion induced by GLP-1 or other cAMP elevating agents [660]. GLP-1-induced elevation of cAMP further increases Na^+^ influx in isolated hamster islets, and this effect is blocked by inhibition of PKA using H-89 [661]. Under conditions of high glucose, the PKA-independent cAMP analog 8-pCPT-2′-O-Me-cAMP also increases Na^+^ influx and insulin secretion in isolated hamster islets, hence indicating that cAMP increases Na^+^ permeability and insulin secretion via PKA-dependent and -independent mechanisms [661]. In summary, elevation of cAMP accelerates insulin secretion via PKA-dependent modulation of ion channel activity, which enhances the influx of Ca^2+^ and Na ^+^ via prolonged cell depolarization.

### 11.3 PKA-independent effects of cAMP on insulin secretion

Apart from accelerating insulin secretion through modulation of ion channel activity, cAMP also affects insulin granule exocytosis through mechanisms that include stimulation of granule mobilization [662], increasing the size of the readily releasable pool of insulin granules [634,663,664], and refilling this pool through enhanced insulin synthesis [46,78,664]. In contrast to initial belief that insulin granule exocytosis is exclusively mediated in a PKA-dependent manner [665], later studies showed that insulin exocytosis is also regulated via PKA-independent mechanisms [664]. In the β-cells, elevation of cAMP leads to activation of *exchange protein activated by cAMP* (Epac2; a.k.a. cAMP-GEFII) [[666], [667], [668], [669]], which regulates insulin granule exocytosis through opening of ryanodine receptor (RYR) Ca^2+^ channels in the ER, and hence by increasing the intracellular pool of Ca^2+^ [667,670]. Epac2-mediated opening of the RYR channels requires the extracellular influx of Ca^2+^ via the VDC channels [667,[671], [672], [673]]. This mechanism of calcium-induced calcium release (CICR) is in line with the observation that cAMP is incapable of promoting insulin secretion under conditions of low glucose [638,641,642], and ensures that the incretins only promote insulin secretion under conditions of hyperglycemia [78,674]. Accordingly, GLP-1R agonists do not induce hypoglycemia in people living with T2D, unless Ca^2+^ influx is increased by adjunctive therapy with sulfonylureas, which induce β-cell membrane depolarization independent of the ambient glucose concentration [[675], [676], [677], [678], [679], [680], [681], [682]]. In line with the role of PKA-dependent and –independent mechanisms in incretin-induced insulin secretion, single inhibition of either Epac2 or PKA decreases insulin secretion induced by GLP-1 or GIP by only ∽50%, while concomitant blockade of Epac2 and PKA decreases incretin-induced insulin secretion by ∽90% [669]. In summary, elevation of cAMP accelerates insulin secretion via Epac2-dependent modulation of RYR channel activity in the ER, which further increases cytosolic Ca^2+^ levels and accelerates insulin secretion under conditions of high glucose.

### 11.4 Regulation of exocytosis

The ability of the incretins to stimulate insulin granule exocytosis is closely linked to their ability to promote cAMP production (Figure 5). In isolated human β-cells, cAMP accelerates exocytosis even under conditions where cytosolic Ca^2+^ levels and membrane depolarization are kept constant [634]. Treatment of isolated islets with cAMP elevating agents is sufficient to accelerate insulin granule exocytosis under conditions of high glucose [634,636,[638], [639], [640], [641], [642], [643],^657^,^683^], and blockade of adenylate cyclase using MDL12330A inhibits GLP-1-induced granule exocytosis [663]. The stimulatory effect of GIP on whole-cell Ca^2+^ current and exocytosis is decreased by ∽65% upon treatment of isolated human β-cells with the PKA inhibitor Rp-8-Br-cAMP [634], indicating that the incretins affect exocytosis through PKA-dependent and -independent mechanisms. Although not yet demonstrated for GIP, GLP-1-induced exocytosis is partially inhibited upon treatment of isolated mouse β-cells with the PKA inhibitor H89 but is fully blocked upon concomitant immunoneutralization of Epac2 [663]. Epac2 regulates exocytosis of the insulin granules by interacting with a series of molecules that regulate transport and fusion of the insulin granules with the plasma membrane, such as Rab3, Rim2 and Piccolo [669,684,685]. When cAMP levels are low, Epac2 is inactive and bound to the SUR1 subunit of the K_ATP_ channels [686]. When levels of cAMP rise, Epac2 becomes activated and dissociates from the SUR1 subunit to form a complex with Rim2 and Piccolo in the cytosol, which then interacts with Rab3 located at the membrane of the insulin granules to promote fusion of the insulin granules with the plasma membrane and excretion of insulin into the circulation [663,686]. Emphasizing their relevance in insulin granule exocytosis, Epac2 interaction with Rim2 and Piccolo is crucial for cAMP-induced insulin secretion and is not blocked by inhibition of PKA [669,684,685].

Apart from regulating insulin secretion via Epac2, GLP-1 (and potentially also GIP) activates protein kinase C (PKC), which depolarizes the plasma membrane and accelerates insulin secretion via activation of *transient receptor potential cation channel subfamily M member 4* (TRPM4) and 5 (TRPM5) [687]. Islets deficient for either TRPM4 or TRPM5 show impaired insulin secretion in response to GLP-1, and PKC-induced membrane depolarization is neither blocked by inhibition of PKA, nor by blockade of K_ATP_ channels or L-Type VDC channels [687]. In line with the multifactorial effect of cAMP on insulin secretion, the rise in intracellular cAMP is paralleled by enhanced first and second phase insulin secretion [638,642,688], and both phases of cAMP-induced insulin secretion are attenuated upon blockade of Epac2 in isolated mouse islets [669]. Nonetheless, while GLP-1 depends on Epac2 to increase the readily releasable pool of insulin granules and to facilitate rapid Ca^2+^-dependent granule exocytosis, the subsequent prolonged granule exocytosis that occurs during continuous cell depolarization is PKA-dependent and is blocked when isolated mouse islets are treated with the PKA inhibitor H-89 [689].

### 11.5 Regulation of insulin synthesis and clearance

Apart from regulating insulin secretion via modulation of ion channel activity and stimulation of insulin granule exocytosis, the incretins are also suggested to improve glucose handling by accelerating insulin biosynthesis [46,78,[690], [691], [692], [693], [694]], and further by decreasing insulin clearance [[695], [696], [697]]. Consistent with the latter, insulin clearance is increased in mice deficient for both incretin receptors [698], but is normal in mice that lack only one of the incretin receptors [699]. The liver is the primary site of insulin clearance [700] and reduced hepatic insulin extraction has been observed in young first-degree relatives of patients with T2D in response to GIP infusion [697]. Studies in healthy humans, however, showed that hepatic insulin clearance is decreased in response to oral glucose but is unaffected by i.v. administration of GIP [182].

Evidence indicating that GIP stimulates insulin synthesis dates back to 1979, when studies in isolated rat islets showed increased radiolabeled (pro)insulin production following treatment with GIP, an observation that was notably unaffected by the ambient glucose concentration [46]. GLP-1 stimulation of insulin production was first shown in rat insulinoma RIN1046-38 cells [78] and later confirmed by other studies [[690], [691], [692], [693], [694]]. GLP-1-stimulation of proinsulin expression is mimicked by forskolin or dibutyryl (db)-cAMP [692,701], indicating that incretin-stimulation of insulin production is cAMP-dependent. Induction of insulin synthesis by cAMP is mediated by Pdx1, which binds to the insulin promoter to initiate proinsulin expression [660,702]. In rat insulinoma RIN 1046-38 cells, expression of *Pdx1* is increased upon treatment with GLP-1, forskolin or 8-Bromo-cAMP, and inhibition of PKA, or antagonism of cAMP, inhibits GLP-1-stimulation of *Pdx1* expression [660]. Further supporting the demonstration that GIP stimulates insulin synthesis, mice with β-cell specific deletion of *Gipr* [703], or with deletion of *Gipr* in cells that express the leptin receptor (*Lepr*) (∽ 75% of embryonic murine β-cells co-express *Gipr* and *Lepr*) [591] show decreased plasma insulin level [591,703], as well and reduced glucose- and GIP-induced insulin secretion [591].

### 11.6 Regulation of glucagon secretion

Even before Brown, Mutt and Pederson isolated GIP from duodenal and jejunal CCK-enriched extracts [24,25], Roger Unger in 1967 showed in dogs, that such impure CCK preparations enhance the release of IR-glucagon from the pancreas [36]. A similar crude CCK preparation was demonstrated in humans to potentiate arginine-induced IR-glucagon [704]. The glucagonotropic effect of the CCK extract was only notable under conditions of hypoglycemia, and was suppressed in dogs upon i.v. infusion of glucose [35]. Soon after its purification, GIP was then shown to increase IR-glucagon in the isolated perfused pancreas of rats [705] dogs [50], and pigs [706], and to potentiate arginine-stimulation of glucagon secretion under conditions of low glucose [705], hence indicating that the glucagonotropic effect of the CCK preparation is mainly attributed to the action of GIP in this preparation. In line with this notion, although *Gipr* deficient mice show normal [[707], [708], [709]] or decreased [112] fasting levels of blood glucose, the glucagonotropic effect of i.v. infused arginine, or orally applied proteins, is blunted in *Gipr* deficient mice [699]. Moreover, while rat GIP(1-42) increases glucagon secretion in the perfused rat pancreas, this effect is blocked by antagonism of GIPR using GIP(3-30)NH2 [710]. The ability of GIP to potentiate arginine-induced glucagon secretion has further been confirmed in isolated mouse islets, along with the demonstration that this effect depends on GIPR signaling in *α*-cells [711].

GIP stimulation of glucagon secretion has been verified in isolated perfused pancreata from humans [51], pigs [706] and rodents [705,710,712,713], in isolated murine islets [49,711,714], and *in vivo* in mice [[715], [716], [717]], rats [49,718,719], dogs [720], and humans [79,422,721]. As demonstrated in the isolated perfused rat pancreas [705] and later confirmed in healthy humans [422], the ability of GIP to stimulate secretion of either insulin or glucagon greatly depends on the level of blood glucose, with GIP potentiating glucagon secretion at blood glucose levels below ∽5.5 mM and stimulation of insulin secretion occurring when glucose concentrations reach or exceed ∽5.5 mM. In line with these observations, studies in healthy humans show that infusion of GIP increases plasma glucagon levels after overnight fasting [721], and that GIP stimulates glucagon secretion in reciprocal relation to blood glucose during euglycemia and hypoglycemia [422], but not under conditions of hyperglycemia [144,422,423]. Nonetheless, while GIP does not stimulate glucagon secretion in healthy individuals under conditions of hyperglycemia [144,422,423,722], it stimulates glucagon secretion in hyperglycemic subjects with T2D [156,476,723,724], and in people with hepatic cirrhosis and hyperglucagonemia [725]. The observation that the insulinotropic but not the glucagonotropic effect of GIP is blunted in people with T2D has led to the hypothesis that an exaggerated glucagonotropic response to nutrient-induced GIP secretion may account for the postprandial hyperglucagonemia that is frequently observed in individuals living with T2D [476]. In line with this notion, GIP inhibits the suppressive effect of glucose on glucagon secretion in isolated murine islets [713], and antagonism of the glucagon receptor improves glucose control in subjects with T2D [726,727]. Infusion of GIP during a mixed meal further increases circulating levels of glucagon in people with T2D, and this coincides with a greater glucose excursion and thus postprandial hyperglycemia [476,728]. However, more recent data have shown that paracrine cross-talk between the islet *α*- and β-cells is crucial for normal nutrient-induced insulin secretion, and that glucagon can stimulate insulin secretion via signaling through the receptors for both GLP-1 and glucagon in the β-cells [550,552,729,730]. This suggests that α-cell-derived glucagon can be insulinotropic through a paracrine stimulation of β-cells within the islets, since administration of glucagon to fed mice decreases rather than increases glycemia by stimulating insulin secretion [729]. Recent research has also indicated that the hyperglucagonemia of type 2 diabetes is more related to disruption of the liver-alpha cell axis which results in elevated levels of amino acids and hence hyperglucagonemia [731].

GIP stimulates cAMP production and/or glucagon secretion in *α*TC1-cells [728] and in isolated rat *α*-cells [732], hence indicating that GIP directly acts on the *α*-cells to enhance glucagon secretion. Interestingly, although GLP-1 and GIP have opposite effects on glucagon secretion *in vivo*, with GLP-1 inhibiting [154,[733], [734], [735], [736], [737], [738], [739]] and GIP stimulating [49,422,[715], [716], [717], [718], [719], [720], [721]] glucagon secretion, they both equally increase whole cell Ca^2+^ currents and exocytosis in isolated rat *α*-cells [740]. This incretin-induced *α*-cell exocytosis is mimicked by forskolin and is suppressed by somatostatin or inhibition of PKA, but is unaffected by treatment with insulin [740]. Thus, these data indicate that *in vitro* both incretins are capable of stimulating glucagon secretion via cAMP/PKA-dependent mechanisms, and that incretin-induced *α*-cell exocytosis is suppressed by somatostatin [740]. The observation that GLP-1 stimulates *α*-cell exocytosis *in vitro* [740], but decreases glucagon levels *in vivo* [144] suggests that GLP-1 indirectly inhibits *α*-cell function via paracrine signaling, potentially via its ability to stimulate pancreatic secretion of somatostatin. In line with this notion, expression of GLP-1R is absent [[741], [742], [743]] or restricted to only a fraction of *α*-cells [744,745], and GLP-1 is considerably more potent than GIP to stimulate the secretion of somatostatin in the isolated perfused rat pancreas [746]. *In vivo*, GLP-1-stimulated secretion of somatostatin may negate the otherwise stimulatory effect of GLP-1 on *α*-cell exocytosis, while GIP-induced glucagon secretion is preserved due to insufficient GIP-mediated induction of pancreatic somatostatin secretion. Continuous glucagon treatment in diet-induced obese (DIO) mice further decreases body weight and improves glucose tolerance with comparable efficacy relative to treatment with exendin-4 [745], an observation that is likely attributed to glucagon's ability to decrease food intake, to increase energy expenditure and to enhance hepatic lipid utilization [745]. These observations spurred interest to engage glucagon receptor agonism in unimolecular formulation with GLP-1R agonism for the treatment of obesity, diabetes, and metabolic dysfunction-associated steatohepatitis (MASH) [295].

## 12 GIP effects on β-cell survival and growth

Beyond stimulating insulin synthesis and secretion, the incretins also improve islet health by stimulating growth, proliferation and survival of the β-cells. Type 2 diabetes is often associated with β-cell dysfunction and decline in β-cell mass due to increased apoptosis and inflammation in response to cellular stress from gluco- and/or lipotoxicity [[747], [748], [749]]. Beta-cell mass can also be depleted in mice by multiple low dose STZ treatment, and twice daily treatment of these mice with [D-Ala_2_]GIP increases islet number and area, with retention of β-cell mass through beneficial effects on proliferation, apoptosis and *α*-cell transdifferentiation [750]. The stable GIP(LysPAL16) analogue has further been shown to enhance functional differentiation of mouse embryonic stem cells into cells expressing islet β-cell-specific genes, including insulin [751]. Cell viability is controlled by a complex signaling network that regulates cellular growth, proliferation and survival via activation of key signaling nodes that include the PKA/CREB, MAPK and PI3K pathways. Induction of PI3K leads to activation of PKB, which inhibits expression and activity of pro-apoptotic factors such as caspase 9, BAD, glycogen synthase kinase-3 (GSK-3), and certain Foxo transcription factors [[752], [753], [754], [755]]. The latter play critical roles in cell cycle progression and survival [[752], [753], [754], [755]] and Foxo1 plays a key role in maintaining β-cell mass and function. In INS-1 β-cells, GIP activates PI3K/PKB [[756], [757], [758]], and this leads under conditions of glucolipotoxic stress to inhibition of Foxo1, with the consequence of its nuclear exclusion and failure of Foxo1 to induce expression of its pro-apoptotic target gene *Bax* [756]. Treatment of INS-1 β-cells with GIP further increases expression of the anti-apoptotic protein Bcl-2 via mechanisms that include PKA/AMPK-dependent regulation of CREB/TORC2 activity [596,756]. Both incretins further promote β-cell survival in INS-1 β-cells via PKB/Akt-dependent inhibition of the *apoptosis signal regulating kinase 1* (ASK1), leading to suppression of MAPK p38 and JNK [759,760]. GIP and GLP-1 further reduce thapsigargin-induced ER stress in purified rat β-cells [761]. Moreover, GIP prevents STZ-induced cell death in INS-1 β-cells in a dose-dependent manner, an effect that correlates with decreased expression of *caspase-3* and *-8* [762]. GIP further decreases apoptosis in glucose-deprived INS-1 β-cells via inhibition of caspase-3 activity, while at the same time dephosphorylating the stress-induced p38 MAP kinase, an effect that is mimicked by forskolin and that is unaffected by inhibition of either PKA, Mek1/2 or PI3K [763]. However, most of the studies reporting β-cell neogenesis, proliferation, and improved survival under toxic conditions after GLP-1 and/or GIP treatment were performed in young rodents, which have an intrinsic ability for β-cell growth. It is notable, therefore, that although the GLP-1R agonist exenatide can induce β-cell proliferation in young rodents, this effect is less obvious in older animals [764]. In contrast to rodents, β-cell proliferation is restricted to three-time windows in humans: in the neonatal period, during puberty, and during pregnancy [765]. Nonetheless, GLP-1-induced β-cell regeneration is accelerated in STZ-treated rodents treated with a chimeric peptide that internalizes via GLP-1R to cell-specifically deliver the nuclear hormone estrogen into these cells [766]. To our knowledge, this has not been studied with respect to GIP-induced β-cell proliferation and targeting, and there is little evidence indicating that GLP-1 or GIP itself affect β-cell proliferation in humans, unless given together with drugs that promote β-cell proliferation [[766], [767], [768]].

## 13 GIP effects in the adipose tissue

### 13.1 GIP effects on lipid storage

**Figure 6:**
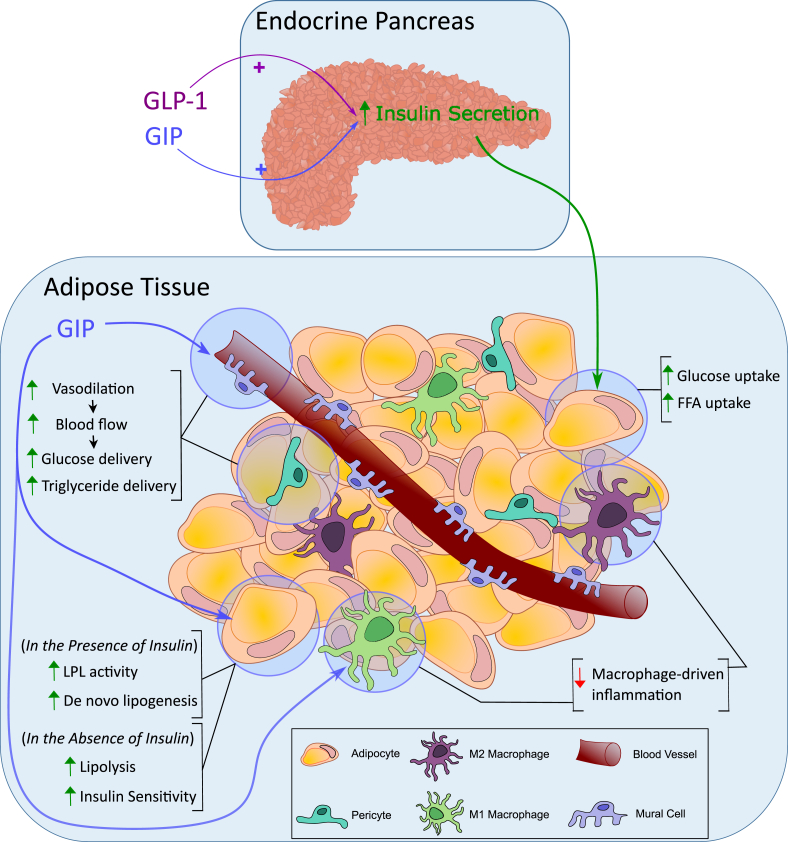
**The effect of GIP on adipose tissue.** Incretin action on the endocrine pancreas stimulates postprandial insulin release. Insulin signalling in adipocytes stimulates glucose and free fatty acid (FFA) update. GIP signals directly in white adipose tissue, stimulating blood flow and the delivery of circulating nutrients, increasing lipoprotein lipase (LPL) activity, de novo lipogenesis, insulin sensitivity and lipolysis. GIP decreases macrophage-dependent inflammation.

The first data indicating that GIP affects adipocyte lipid metabolism date back to 1976, where studies in isolated rat adipocytes showed that GIP inhibits lipolysis by suppressing glucagon-induced cAMP production [769,770] (Figure 1). Subsequently, GIP was shown to stimulate adipocyte lipid uptake by increasing the synthesis and release of lipoprotein lipase (LPL) in 3T3L1 adipocytes [[599], [600], [601]], and in adipocytes isolated from humans [600,602] and rats [771] (Figure 6). LPL catalyzes the breakdown of dietary lipoprotein-linked triglycerides into 2-monoacylglycerol and fatty acids, which are then taken up into the adipocytes to undergo triglyceride re-esterification [[772], [773], [774], [775]]. Supporting this notion, infusion of GIP at near physiological levels increases chylomicron-linked triglyceride breakdown in dogs [776], while immunoneutralization of endogenous GIP increases plasma triglyceride levels after intraduodenal lipid infusion [769]. The lipid storage effect of GIP is augmented by co-infusion with chylomicrons [776] and is not observed in healthy humans [777] and dogs [778] upon infusion of lipoprotein free lipids, or in the absence of insulin [779].

GIP-induced LPL activation and lipid storage is predominantly observed when GIP is given in addition to insulin [[600], [601], [602]] (Figure 6). As demonstrated in obese VDF rats*,* as well as in 3T3-L1 adipocytes and isolated human preadipocytes, GIP increases the phosphorylation of PKB in the presence of insulin, while decreasing phosphorylation of LKB1 and AMPK, leading to activation of LPL and acceleration of adipocyte lipid storage [600]. The ability of GIP to promote lipogenesis via the PKB/LKB1/AMPK/LPL pathway is potentially mediated by the adipokine resistin, which is released from 3T3-L1 adipocytes and the epididymal white adipose tissue (eWAT) of VDF rats upon treatment with GIP, and which mimics the effects of GIP to activate LPL via the PKB/LKB1/AMPK pathway [601]. As shown in human adipocytes, and in HEK293 cells transfected to express GIPR, GIP also stimulates phosphorylation of cAMP-response element binding protein (CREB) and nuclear translocation of the CREB coactivator 2 (TORC2), which binds to the LPL promoter to induce LPL expression [602]. GIP further increases GLUT4 translocation in 3T3L1 adipocytes [594] and enhances insulin receptor affinity in isolated rat adipocytes, thereby stimulating insulin-induced glucose uptake [594,604,605], and conversion of glucose into lipids [604] (Figure 6). GIP also stimulates fatty acid synthase in rat adipose tissue explants [780], increases expression AP2 (a.k.a. FABP4), a marker indicative of adipocyte differentiation, in 3T3L1 adipocytes [594], and promotes adipocyte lipid deposition by increasing adipose tissue blood flow in healthy humans under hyperinsulinemic/hyperglycemic clamp conditions [779,781] (Figure 6). GIP-induced vasodilation is blunted in adipose tissue of people living with obesity and insulin resistance [782], and in healthy humans upon antagonism of GIPR [781], and normalizes upon weight loss and restoration of insulin sensitivity [782]. GIP also suppresses adipose tissue inflammation, as assessed through reduced cytokine expression and decreased adipose tissue infiltration of IFN-γ-producing CD8(+) and CD4(+) T cells in mice with diet-induced obesity [783]. In contrast, deletion of *Gipr* in immune cells augments adipose tissue inflammation and insulin resistance, whereas co-deletion of *GIPR* and S100A8/A9 in immune cells ameliorates the dysregulated metabolic and inflammatory phenotypes observed in HFD-fed mice with myeloid deletion of *Gipr*, consistent with a suppressive role for GIP in the control of myeloid cell mediated adipose tissue inflammation [784]. In mice, GIPR agonism further decreases gut inflammation induced by 5-fluorouracil (5FU), and while 5FU-induced gut inflammation is enhanced in *Gipr* deficient mice, it is decreased in these mice after transplantation of bone marrow-derived GIPR cells [785].

### 13.2 GIP effects on adipocyte lipolysis

The observation that GIP inhibits lipolysis by suppressing glucagon-stimulated cAMP production [769,770] is somewhat unexpected, given that GIP increases cAMP production in a variety of cells, including isolated pancreatic islets [48,636], mouse betaTC-3 cells [168], hamster In11 β-cells [637], rat insulinoma RINm5F cells [545], isolated rat *α*-cells [732], *α*TC1-cells [728], HEK293 cells transfected to express GIPR [548], human subcutaneous adipocytes [559] and also isolated rat adipocytes [769] and differentiated 3T3L1 adipocytes [597]. Given that cAMP stimulates lipolysis in adipocytes [[786], [787], [788]] via activation of hormone-sensitive lipase (HSL) [789], and that insulin promotes its anti-lipolytic action by decreasing cAMP production [[790], [791], [792], [793]], GIP-induced cAMP production is hence expected to stimulate lipolysis rather than lipogenesis. Indeed, under normo- or hypoinsulinemic conditions, GIP-induction of cAMP production increases lipolysis in isolated rat adipocytes [769] and in differentiated 3T3L1 adipocytes [597], and GIP-induced lipolysis is antagonized by the addition of insulin or upon inhibition of adenylate cyclase using MDL12330A [597] (Figure 6). In isolated human adipocytes, GIP stimulates lipolysis via activation of HSL [794] and GIP promotes lipolysis in insulin resistant DIO mice [795], and in hyperglycemic people with T1D during basal insulin substitution [796]. In contrast to GIP, glucagon fails to stimulate lipolysis in 3T3L1 adipocytes, excluding a role of the glucagon receptor in the lipolytic action of GIP [597]. In DIO mice, treatment with a long-acting GIPR agonist decreases fat mass [581,591,795,797] and expression of proinflammatory genes in the adipose tissue [797], and this is paralleled by increased activity of LPL [797] and enhanced fatty acid oxidation [795]. Mice with overexpression of GIP [798], or with adipocyte-specific overexpression of *Gipr* [799], are relatively lean and show decreased fat mass when chronically exposed to a HFD. Similar results are observed in DIO mice treated with long-acting GIPR agonists [581,591,795]. In line with this is the observation that the body weight lowering effect of peripherally administered acyl-GIP is, in some studies, partially preserved in mice with CNS-deletion of *Gipr*, despite complete blockade of GIP-induced inhibition of food intake [795], hence indicating that GIPR agonism also decreases body weight via peripheral mechanisms not related to food intake. In summary, GIP promotes triglyceride storage under conditions of hyperinsulinemia, but enhances lipolysis and fatty acid oxidation under conditions of normo- or hypoinsulinemia [598] (Figure 6).

### 13.3 Data from GIPR deficient mice

Mice with global *Gipr* deficiency show decreased fat mass and protection from obesity induced by either ovariectomy [800] or HFD-feeding [[801], [802], [803], [804]]. Protection from diet-induced obesity is however also observed in some experiments studying mice that lack the GLP-1 receptor [805,806], and further, in mice with concomitant deletion of both incretin receptors (DIRKO) [805]; these findings complicate considerations of the pharmacological potential of GIPR signal modification. Nonetheless, the metabolic phenotype seen in *Gipr*-deficient mice is seemingly in line with a physiological role of GIP to promote adipocyte lipid deposition. Mice with Adipose protein 2 (Ap2)-Cre-mediated deletion of *Gipr* show decreased hepatosteatosis and improved glucose tolerance but exhibit only slightly decreased body weight without changes in fat mass [807]. Another study using mice with Ap2 Cre-mediated deletion of *Gipr* show decreased expression of *Gipr* in the white and brown adipose tissue, but these mice do not show overt differences in body weight, fat mass or glucose metabolism when fed with a HFD [589]. When kept at room temperature, mice with specific deletion of *Gipr* in the brown adipose tissue (BAT) show no protection from diet-induced obesity, but display slightly decreased body weight and enhanced BAT function when exposed to cold, or when treated with a β-adrenergic receptor agonist [808]. Lack of protection from diet-induced obesity is also observed in mice with Adiponectin Cre-mediated deletion of *Gipr,* but these data have to be regarded with caution, since these mice do not show alterations in *Gipr* expression in the white and brown adipose tissue [589]. In summary, data in mice with adipocyte-specific deletion of *Gipr* show no major differences in body weight and body composition, although a series of data show GIP to autonomously act on the adipose tissue to affect either lipid storage or utilization. Continuous infusion of GIP for 6 days nonetheless increases hepatic fat content in people living with T1D [809], and fat-specific overexpression of GIPR leads to protection from diet-induced obesity [799], overall supporting that GIP increases adipose tissue lipid utilization under conditions of insulin resistance.

## 14 GIP effects on the bone

Resistance to bone fracture is complex and dependent on bone mass, bone microstructure, and the properties of molecules composing the bone matrix, i.e. bone material properties [810]. Bone tissue is composed of trabecular and cortical compartments subjected to mineral and energy metabolism as well as mechanical stresses [811]. Bone mass, microstructure and material properties are constantly adapted to mechanical or metabolic stresses through a controlled and coupled action of bone cells during the bone remodeling process, represented by osteoblast-mediated bone formation and osteoclast-induced bone resorption [812]. Expression of *GIPR/Gipr* has been demonstrated in osteoblasts, osteoclasts, osteocytes, bone marrow pluripotent mesenchymal cells and osteoblast- and osteoclast-like cell lines [585,[813], [814], [815], [816], [817], [818], [819]]. Individuals that carry a functional mutation in *GIPR* that decreases signaling have been reported to have lower bone mineral density and an increased risk for fractures [820]. Moreover, in a large study of more than a million individuals, loss of function *GIPR* variants were not associated with reduced BMI were associated with a risk of reduced bone density or increased risk of fractures [821].

Bone remodeling is a bi-directional process that is tightly controlled by osteoblast-mediated bone formation and osteoclast-induced bone resorption [822]. This process is dynamically linked to nutrient availability, with increased bone resorption and decreased bone formation during fasting, and decreased bone resorption with increased bone formation postprandially [823]. Osteoporosis and loss of bone mass is frequently observed in patients receiving long-term parenteral nutrition [824,825], and bone remodeling is greater upon oral relative to intravenous glucose administration [826], indicating that bone remodeling is potentially under control of gastrointestinal hormones, including the incretins and the related peptide GLP-2 [822,827,828]. The gut hormone-bone resorption axis is conserved in humans [829,830], as inhibition of gastro-pancreatic peptide secretion through infusion of octreotide blocks the inhibitory effect of orally ingested glucose on bone turnover in healthy humans [829].

**Figure 7:**
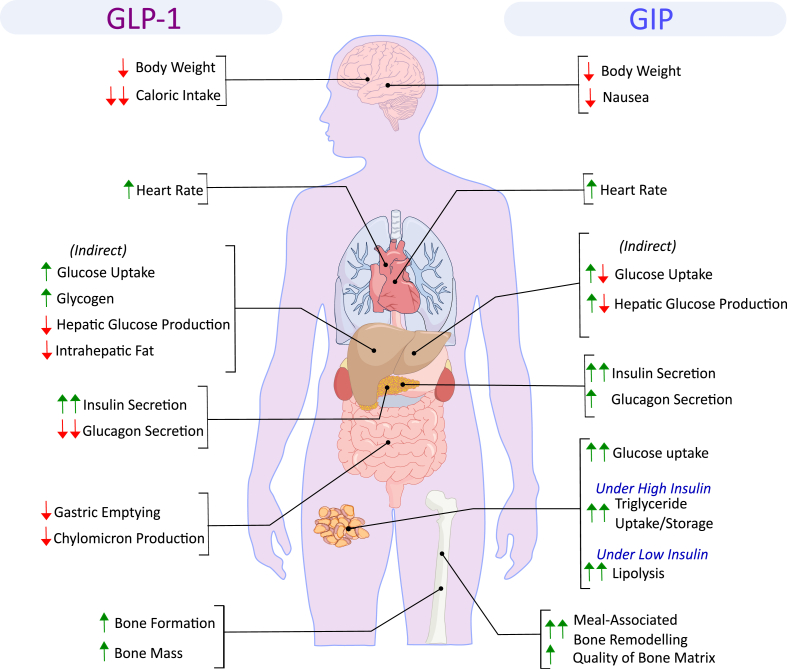
**Schematic on the effects of GIP and GLP-1 on metabolism and energy balance.** In addition to their role in regulating postprandial insulin release, both GIP and GLP-1 target multiple organ systems to affect energy balance. These include actions targeting the brain, cardiovascular system, liver, gastrointestinal tract, adipose tissue, and bone.

At the cellular level, GIP promotes cAMP production and accelerates intracellular Ca^2+^ levels in human osteosarcoma SaOS2 cells, and this is associated with increased expression of type I collagen, and enhanced activity of alkaline phosphatase (ALP), both markers indicative of enhanced bone matrix deposition [585,816]. GIP further inhibits apoptosis and stimulates proliferation and differentiation of osteoblastic cells [817]. While GIP effects on osteoblast-mediated bone formation have been confirmed in numerous studies, GIP effects on osteoclast function are somewhat controversial, with GIP showing either no [817] or an inhibitory [814,818] effect on bone resorption (Figure 7). In isolated human osteoclasts, GIP inhibits osteoclastogenesis and delays bone resorption, while increasing osteoclast apoptosis via mechanisms that include impaired nuclear translocation and action of NFATc1 and NFKB [831]. In primary human bone cells, GIP reduces osteoclast activity and improves osteoblast survival, and these effects are antagonized by treatment with GIP(3-30)NH2 [814]. In agreement with an anabolic role of GIP on bone formation, infusion of GIP decreases markers of bone resorption in healthy humans [823], postmenopausal women [832], and in people with type 1 or type 2 diabetes [833,834]. Nonetheless, in subjects living with T1D, GIP suppression of markers indicative of bone resorption was no longer evident after 6 days of continuous GIP infusion [809].

Transgenic mice overexpressing GIP show increased bone mass, and this is associated with elevated levels of markers indicative of bone formation, and decreased levels of markers indicative of bone resorption [835] (Figure 7). In contrast, global *Gip* deficient mice have decreased trabecular bone mass that is associated with an increase of osteoclasts [836]. Alterations in bone mass in *Gipr*-deficient mice seems to be age-dependent, with low trabecular bone mass in young animals and high trabecular bone mass in aged animals [817,837]. However, mice deficient for either *Gip* or *Gipr* exhibit reduced cortical bone mass and cortical thickness [836,838]. *Gipr*-deficient mice further show reduced bone marrow, along with a decreased number and proliferation of hematopoietic stem and progenitor cells (HSPCs) [839].

Loss of *Gipr* in mice also impairs bone marrow hematopoiesis [839] and dysregulates the hematopoietic bone marrow responses to energy excess, several Toll-Like receptor ligands, and the cytoxic agent 5-Fluourouracil [840]. Administration of human GIP analogues prevents osteoclast-mediated bone loss in mice with bone fragility induced by ovariectomy [841]. In vitro, GIP also modulates the quality of the bone material itself by increasing the expression of lysyl oxidase, resulting in higher enzymatic crosslinking of the collagen matrix in osteoblast-like MC3T3-E1 cells through an adenylate cyclase – β-catenin pathway [815,842]. *Gip-* and *Gipr*-deficient animals also show lower enzymatic collagen crosslinking that is directly correlated to lower mechanical resistance [836,838]. Administration of GIP analogues further enhances bone mineralization and enzymatic collagen crosslinking in rats [843], as well as in several mouse models of bone fragility, including mice with T1D and T2D [844,845].

Based on the ability of GIP and GLP-2 to affect bone remodeling and material properties [832,846], several GIP/GLP-2 co-agonists were developed and tested for their ability to affect bone fragility in preclinical studies [819,847]. Preliminary data in rodent models of bone fragility showed that these molecules potently improve bone strength by restoring enzymatic collagen crosslinking [847]. Further studies are required to assess whether such effects could also be encountered in humans affected by bone fragility disorders such as osteoporosis. In summary, GIP improves bone strength by enhancing bone material properties but also increases bone mass by stimulating osteoblast-mediated bone formation while at the same time suppressing osteoclast-mediated bone resorption (Figure 7). The observation that GIP directly inhibits bone resorption and stimulates bone formation in osteoclasts and osteoblasts, respectively *ex vivo* indicates that the anabolic and anti-resorptive effects of GIP on bone formation may be insulin-independent.

## 15 GIP effects on the cardiovascular system

### 15.1 GIP effects on the heart

Expression of *Gipr* has been shown in all four chambers of the murine [848,849] and human [849,850] heart, in mouse atrial cardiomyocyte HL-1 cells [849], and in isolated murine cardiomyocytes [848,849,851]. An acute increase in heart rate of ∽5-10 bpm, along with an acute decrease in blood pressure of ∽10–15 mmHg has been shown after GIP infusion in healthy humans [852,853], as well as in obese humans with normal or impaired glucose tolerance and T2D [438,854] (Figure 7). GIP effects on heart rate and blood pressure are transient and most pronounced within the first 60–90 min of GIP infusion [853,855]. An increase in heart rate of ∽10 bpm is also observed after GIP infusion in people with T1D clamped either at hyper- or hypoglycemia, along with a decrease in diastolic blood pressure of ∽5 mmHg and an increase in systolic blood pressure of ∽6 mmHg [855]. In contrast, chronic treatment with the long-acting GIPR agonist NNC0480-0389 for 34 weeks resulted in a small decrease in systolic blood pressure (EudraCT Number 2020-004863-14-Clinical trial results-EU Clinical Trials Register). Alternatively, a decrease in systolic but not diastolic blood pressure has been reported in humans that carry a missense mutation in the *GIPR* gene [856]. Nevertheless, at the age of 10–12 wks, mice with global *Gipr* deficiency do not show alterations in cardiac function, including heart rate, ejection fraction, fractional shortening, and left ventricular (LV) diameter [849,857], but these mice exhibit increased survival after ischemic cardiac injury and myocardial infarction (MI) [849]. Mice with targeted deletion of *Gipr* in adult cardiomyocytes further show reduced infarct size and improved survival after ischemic injury [849]. Protection from cardiac injury in *Gipr* deficient mice is not associated with changes in the phosphorylation of AKT, GSK3β or AMPK, or with markers indicative of MI, such as *macrophage inflammatory factor-1* (*MIF-1*), *secreted protein acidic and rich in cysteine* (*SPARC*), *caveolin-1*, and *angiotensin-converting enzyme 2* (ACE2), but is paralleled by increased myocardial TAG content and decreased activity of HSL in the viable myocardium [849]. Furthermore, GIP perfusion increases myocardial fatty acid oxidation, and treatment of cardiomyocyte HL-1 cells with [D-Ala_2_]GIP increases intracellular TAG level, and this effect is attenuated by genetic silencing of HSL [849]. One week daily treatment with [D-Ala_2_]GIP does not affect mortality in mice with experimental MI, but increases scar formation after experimental ischemia [849]. The cardioprotective effect of GIPR signal inhibition might be age-dependent, since *Gipr*-deficient mice show normal cardiac function at the age of 10–12 weeks [849,857], but LV dysfunction, associated with increased cardiac ceramide accumulation, cardiac apoptosis, oxidative stress and cardiac fibrosis at the age of 50 weeks [857]. Challenging these data, however, *Gipr* deficient mice show an extended lifespan without alterations in muscle endurance [858]. The cardioprotective effect of GIPR deficiency further seems to be restricted to myocardial infarction and is not observed in mice with experimental heart failure induced by treatment with doxorubicin [849].

Diabetic cardiomyopathy, commonly described as cardiac hypertrophy and decreased cardiac function in the absence of coronary artery disease and hypertension in individuals living with diabetes, is linked to an elevated risk of heart failure (HF) and mortality in people living with diabetes [859]. In wildtype and ApoE KO mice, GIP suppresses cardiomyocyte hypertrophy induced by treatment with Angiotensin II (Ang II), and this is paralleled by decreased cardiomyocyte apoptosis and reduced interstitial fibrosis [848]. The beneficial effects of GIP on Ang II-mediated cardiomyocyte hypertrophy are GIPR-dependent and are not observed in *Gipr* deficient mice [848]. In isolated murine cardiomyocytes, GIP increases cAMP level and decreases Ang II-induced expression of B-type natriuretic peptide (*Bnp*) and *Tgf-β1*, both of which are biomarkers of cardiac dysfunction and hypertrophy [848]. Decreased Ang II-mediated expression of *Tgf-β1* is also observed after GIP treatment in ApoE KO mice, and this is paralleled by decreased expression of *hypoxia inducible factor-1α* (*Hif-1α*) [848], a proinflammatory transcription factor that promotes cardiac hypertrophy in mice [860]. GIP further decreases cardiac hypertrophy and interstitial fibrosis in diabetic db/db mice and decreases NADPH oxidase-driven superoxide production and expression of markers indicative of fibrosis (*Ctgf*) and heart hypertrophy (*β-Mhc* and *Tgf-β2*) in isolated mouse cardiomyocytes [861]. In summary, GIPR signal inhibition shows cardioprotective effects under conditions of ischemic injury and MI, while GIPR signal amplification shows cardioprotective effects in animal models for cardiomyopathy. In line with a potential cardioprotective effect of GIP are small clinical studies showing that lower circulating levels of GIP are associated with higher mortality and worse CV outcome in high-risk patients with acute MI [862], and that serum levels of GIP are decreased in people with ST-elevation myocardial infarction (STEMI) relative to individuals without MI [863].

Fasting levels of GIP are difficult to compare across studies due to high variability of different assays. However, a meta-analysis comprising 8,044 subjects shows that elevated fasting levels of GIP are associated with a higher risk of mortality and death from CV diseases [864]. However, it is important to note that plasma GLP-1 level is a predictor of poor CV outcomes in patients with acute MI [865], yet chronic pharmacological agonism of the GLP-1 receptor produces beneficial CV outcomes in patients with obesity and/or T2D. Furthermore, lower GIP levels are associated with poorer CV outcome in high-risk patients with acute MI [862]. Relative to healthy controls, fasting levels of GIP are further increased in individuals with a history of CVD, and expression of *GIPR* is elevated in the carotid arterial wall of subjects with symptomatic CVD [866]. Caution is urged against inferring causality from this association when examining epidemiological data.

Human genetic epidemiologic investigations have probed the role of GIP and the GIP receptor in modulating CV risk. No association with CVD has been observed for the common *GIP* variant rs2291726 (GRCh38.p14; 17:48961892; NC_000017.11:g.48961892T > A), which leads to a C-terminally truncated GIP due to the lack of coding exons 5 and 6 [867]. Moreover, in a cohort of South Indian subjects, rs2291726 showed no association with T2D, but the minor risk G-allele was associated with higher levels of total and LDL cholesterol [868]. An association with CVD has been reported for the GIPR rs1800437 minor C-allele (GRCh38.p14; 19:45678134; NC_000019.10:g.45678134G > C) [867], which causes an E354Q substitution in the 6th transmembrane domain with result of reduced basal but not ligand-induced GIPR activity [869], whereas other studies have shown unaltered or even increased basal and/or ligand-induced cAMP production [[870], [871], [872]]. This variant has previously been shown to be associated with decreased BMI [873,874] and resistance to diet-induced obesity [872], as well as with elevated postprandial levels of blood glucose [820], and lower bone mineral density [820]. A subsequent association study, however, showed that the association of *GIPR* rs1800437 with CVD is GIPR-independent, and rather mediated by the intronic variant rs1964272 (GRCh38.p14; 19:45687010; NC_000019.10:g.45687010G > A) in the SNRPD2 gene, and which is in linkage disequilibrium with rs1800437 [875]. The minor risk A-allele of rs10423928 (GRCh38.p14; 19:45679046; NC_000019.10:g.45679046T > A) is nonetheless reported to be associated with decreased GIPR function [876] and with an increased risk of stroke in people with T2D [866]. However, more recent genetic epidemiologic analyses have effectively dissociated the CV risk signal from the GIPR locus, contrasting the genetic evidence associated higher fasting GIP levels with increased CVD risk, and are rather attributing this risk to a known CAD risk locus in high linkage disequilibrium with the GIPR locus [875]. Another recent study provided genetic evidence to support a beneficial role of sustained GIP signaling on cardiometabolic health greater than that expected from improved glycaemic control alone [877]. In total, human genetics that assessed the association of GIP/GIPR variants with CV diseases are conflicting, but more recent data support that sustained GIP signaling will have no adverse effect on CV risk and may in fact offer benefit.

### 15.2 GIP effects in the vasculature

As demonstrated in healthy humans, ingestion of a mixed meal increases blood flow in the jejunum and the pancreas, but not the duodenum, and these effects are mimicked by infusion of GIP, but not of GLP-1 [878]. GIP further increases blood flow in the human femoral [852] and splanchnic [878] arteries, as well in the mesenteric artery and portal vein of cats [879] and dogs [880], while decreasing blood flow in the pancreatic and hepatic arteries [879,880]. Along with the observation that GIP increases blood flow in the human adipose tissue [779,881] (Figure 6), these data align with the assumption that GIP increases mesenteric blood flow to optimize nutrient utilization and deposition. Interestingly, local endothelial cells vary in the extent of their responses to GIP with cAMP production and Ca^2+^ signaling, an observation that might be explained by the differential abundance of *Gipr* splice variants in the respective vascular beds [882,883].

Expression of *Gipr*/*GIPR* has been demonstrated in the murine [884] and human [882] aorta, the human pulmonary [882] and myometrial arteries [884], the rat carotid [884] and the pig coronary artery [884]. In all these vascular beds, *Gipr/GIPR* is predominantly expressed in the arterial endothelium, with far lower expression in the smooth muscle cells of the media [884]. *Gipr* is also expressed in the rat endothelium of the large peripheral and central blood vessels [541], the rat cardiac endothelium [541], human umbilical vein endothelial EC304 cells [882], human mesenteric endothelial cells (HMECs) [884], human umbilical vein endothelial cells (HUVECs) [882,884], human pulmonary artery endothelial cells (HPAECs) [882], human aortic endothelial cells (HAECs) [882], and human aortic smooth muscle cells (HASMCs) [885]. Factors implicated in the regulation of arterial blood flow include endothelin-1 (ET-1), a vasoconstrictor that is increased in atherosclerotic plaques [886] and in the circulation of people with CV diseases [887,888], and which is elevated following GIP treatment in isolated canine HAECs [889], cultured intact mouse aortas [866], and HUVECs [883,889]. In portal vein endothelial cells (PVECs), GIP fails to induce ET-1 secretion, but stimulates production of nitric oxide (NO) [866], which is produced from l-arginine through the action of the endothelial NO synthase (eNOS) in the presence of oxygen [890], and which promotes vasodilation by counteracting the vasoconstrictive capacity of ET-1 via activation of the eNOS/NO/cGMP pathway [890,891]. GIP promotes NO production in HUVECs, and this effect is abrogated by inhibition of CaMKK, PLC, or AMPK [885]. Nonetheless, levels of eNOS and NO are decreased in HUVECs under conditions of high glucose, and this effect is reversed by treatment with GLP-1, but not with GIP [892].

The observation that GIP stimulates endothelial expression and/or secretion of ET-1 [866,883,889] suggests that GIP might be pro-atherogenic, given that ET-1 is abundantly expressed in atherosclerotic lesions, where it stimulates proliferation and migration of VSMCs [886,893,894]. In HUVECs, secretion of ET-1 is induced by treatment with LDL but not HDL cholesterol [895], and while circulating levels of ET-1 are elevated in hypercholesterolemic rats [896], rabbits [897], and humans [898], antagonization of the ET-1 receptor decreases the number and size of macrophage-foam cells in hypercholesterolemic hamsters [899], while decreasing atherosclerotic plaque formation and increasing NO mediated endothelium relaxation in ApoE KO mice [895,900]. ET-1 promotes its atherogenic action at least in part by stimulating the expression and secretion of osteopontin (Opn), which promotes macrophage activation and differentiation, monocyte infiltration, as well as VSMC migration and proliferation within the atherosclerotic plaques [901]. Plasma levels of ET-1 and Opn are positively correlated in people with critical limb ischemia, and while GIP-stimulation of ET-1 increases expression and/or secretion of Opn in the cultured intact mouse aorta and in healthy humans, this effect is blocked by pharmacological inhibition of ET-1 [866]. Despite the variable precision of measuring fasting GIP, elevated levels of fasting GIP correlate positively with subclinical atherosclerosis in humans [902]. Nonetheless, in contrast to these data indicating that GIPR agonism might be pro-atherogenic, higher glucose-stimulated GIP secretion is associated with lower LDL cholesterol and higher HDL cholesterol, independent of insulin in both men and women [496], and treatment of hypercholesterolemic LDLR-deficient mice with a long-acting GIPR agonist decreases LDL cholesterol and atherosclerosis at doses subthreshold to affect food intake and body weight [903]. These data align with studies in ApoE KO mice, showing that both incretins decrease atherosclerotic lesions and macrophage infiltration in the aortic wall, and these effects are blocked by antagonization of either GLP-1R or GIPR [904]. A GIP-induced decrease in atherosclerotic lesions is also observed in STZ-treated diabetic ApoE KO mice, and GIP decreases macrophage foam cell formation in db/db and ApoE KO mice [905]. The DPP4 inhibitor vildagliptin, which increases levels of endogenous active GIP, suppresses atherosclerotic lesions, macrophage accumulation and foam cell formation in non-diabetic ApoE Ko mice, and while these effects are partially blocked by inhibition of either GLP-1R or GIPR, they are fully blocked by concomitant antagonization of both incretin receptors [906].

Apart from having beneficial effects on atherosclerosis, GIP is also reported to protect from the detrimental effects of arterial remodeling after endothelial injury [885,907]. Neointimal hyperplasia (NH) is frequently observed upon endothelial injury, and is characterized by migration of VSMCs into the tunica intima with the result of decreased luminal space due to thickening of the arterial walls [907]. *Gipr*-deficient mice show increased NH in response to transluminal wire injury, and NH is suppressed in wildtype mice following treatment with GIP [885]. In agreement with the ability of GIP to suppress arterial remodeling after endothelial injury, GIP inhibits NH in injured femoral murine arteries, decreases vascular cell proliferation in the neointima and media, and accelerates endothelial regeneration without affecting injury-induced vascular inflammation [885]. In HUVECs, GIP inhibits the production of reactive oxygen species (ROS) induced by advanced glycated endproducts (AGEs), while decreasing the expression of *vascular cell adhesion molecule-1* (*VCAM-1*) and *plasminogen activator inhibitor-1* (*PAI*), both markers indicative of vascular damage and enhanced mortality due to CVD [884]. Moreover*, Gipr*^−/−^ mice in the *Apoe*^−/−^ background also exhibit upregulation of inflammation-related gene expression in the aorta and increased atherosclerosis despite lower numbers of circulating myeloid cells and reduction in body weight [908]. In summary, GIP effects on the CV system are conflicting, with amplification and inhibition of GIPR signaling both having beneficial and detrimental CV effects, depending on the species and the genetic and/or experimental model.

## 16 GIP effects on the reproductive system, PCOS and menopause

There is emerging evidence that GIP may play a role in both the female and male reproductive systems and in fertility [909,910]. Much of this is based on observations made in women with PCOS including the observation that bariatric surgery which alters the gut hormone landscape can induce spontaneous ovulation with this condition in association with obesity [911]. Polycystic ovary syndrome (PCOS) is linked to a higher risk of T2D and is reportedly associated with increased GIP and lower GLP-1 responses to meal ingestion or to an oral glucose load [[912], [913], [914]], in agreement with the often elevated BMI in these individuals. Although not fully consistent [915,916], elevated GIP has been proposed as a predictive risk factor for PCOS [912]. Indeed, a possible role for GIP in PCOS is emerging [909,910], with raised GIP concentrations potentially inducing an unfavorable environment by increasing insulin, adrenal cortisol and androgens, while decreasing adipose tissue 11beta-HSD1, pituitary follicle-stimulating hormone (FSH) and ovarian progesterone synthesis [909].

Other studies have assessed the possible contribution of disturbances of GIP secretion in age-related deterioration of glucose tolerance [193,194,917]. Normal or slightly higher GIP responses to oral glucose were observed in elderly women, indicating that changes of insulin secretion and action are more important. The heightened GIP responses in older women may reflect the changing hormonal environment with the menopause, and consistent with that, hormone replacement therapy has been shown to reduce both fasting and postprandial GIP [918]. Enhanced fertility and pregnancy have also been observed in women taking either long-acting GLP-1 or dual GLP-1/GIP mimetics for treatment of obesity [910]. The effect of weight reduction and alleviation of insulin resistance per se may be key behind such effects but *GIPR* gene expression is present in female reproductive tissues including pituitary, ovaries and uterine horn, with corresponding protein expression confirmed histologically in these tissues using *GIPR*-Cre mice where the receptor expressing cells were fluorescently tagged [588]. GIP has also been shown to impair FSH-induced progesterone production via effects on ovarian granulosa cells [919]. Further, functional studies using *Gipr* KO mice have revealed disturbed estrous cycling and reduced fertility and litter size over three breeding cycles [588]. It is also worth noting that HFD feeding of female rats leads to disrupted estrous cycling and fertility which is associated with upregulation of *Gipr* in ovary and adrenal glands [920], presumably reflecting activation of steroidogenesis and increased plasma glucocorticoid concentrations. In male mice, the GIPR has been reported on spermatids and sperm from *Gipr* KO mice was associated with lower rates of *in vitro* fertilization [921]. More research is needed in this area, but these early data are intriguing in terms of uncovering a possible unsuspected role of GIP in the link between nutritional and reproductive status, together with the possibility of opening new approaches to treatment of infertility.

## 17 GIP effects in the brain

**Figure 8:**
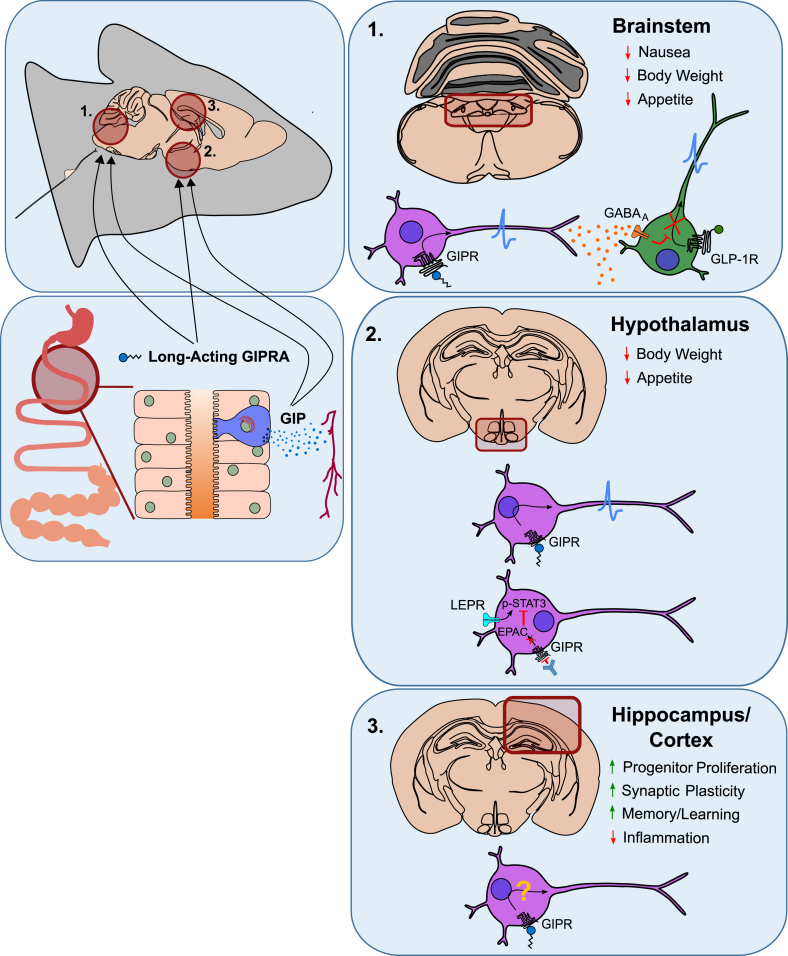
**The central effects of GIP and GIP pharmacology.** The central GIPR signaling axis is engaged by stabilized GIP analogues and GIP released from the gastrointestinal epithelium. In the brainstem, GIPR agonism stimulates the release of GABA which binds GABA_A_ receptors on neighboring GLP-1R cells to inhibit nausea and malaise. Direct activation of GIPR neurons in the area postrema (AP) and the nucleus tractus solitarius (NTS) suppresses appetite. In the hypothalamus, activation of GIPR neurons decreases food intake. Alternatively, GIPR antagonism has been shown to alleviate hypothalamic leptin resistance. In the hippocampus and cortex, GIP promotes synaptic plasticity and progenitor cell proliferation to enhance memory and learning while reducing markers of inflammation.

Data demonstrating that GIP acts centrally originate from studies in the mid 1980's, showing that administration of porcine GIP into the hypothalamic 3rd ventricle of female rats stimulates hormonal release from the anterior pituitary [922]. Later, enzyme-resistant analogues of GIP were shown to reverse defects in hippocampal synaptic plasticity and cognitive function induced in mice either by intracerebral beta-amyloid injection or consumption of HFD [923,924] (Figure 8). *Gipr* KO mice were also shown to exhibit impairments in learning, synaptic plasticity and hippocampal neurogenesis, indicating a direct involvement of GIP [925]. Subsequent studies then showed that GIPR agonism has neuroprotective effects in mouse models for Alzheimer's and Parkinson's disease [[926], [927], [928], [929]], ameliorates drug-induced emesis [[930], [931], [932]], and decreases body weight in rodents through centrally-mediated inhibition of food intake [581,795] (Figure 8). Data related to GIP's effects on body weight and food intake are nonetheless conflicting, not only with discrepant results observed depending on the molecule and the underlying species, but also related to the observation that GIPR agonism and antagonism both decrease body weight and food intake in experimental animals, particular when given in combination with GLP-1R agonists.

### 17.1 Regulation of body weight and food intake by GIPR agonism

Soon after its discovery, GIP was shown to not affect food intake when infused either peripherally [933] or centrally [934] in rats. Along with the obesity-protecting phenotype seen in *Gipr* deficient mice [[801], [802], [803], [804]], and the diminished insulinotropic action of GIP in people living with T2D [144,[147], [148], [149], [150], [151]], these data initially suggested that GIP may have no pharmacological potential for the treatment of obesity and diabetes. But caution is warranted when interpreting these data, since protection from diet-induced obesity was also initially reported with genetic [805,806] or pharmacological [806] inhibition of the GLP-1R, which subsequently was demonstrated to be an excellent drug target for weight loss. In line with the expression of *Gipr* in brain feeding centers of the hypothalamus [587,591,608,609,795] and the hindbrain [607,610,611], more recent studies show that long-acting fatty acid acylated (acyl) GIPR agonists decrease body weight and/or food intake in DIO mice [581,795,806,935,936], and that GIP synergizes with GLP-1R agonism to yield greater weight loss and further inhibition of food intake relative to treatment with GLP-1 alone [177,935]. The ability of peripherally administered long-acting GIPR agonists to decrease body weight is not related to changes in energy expenditure [795] and is preserved in GLP-1R deficient mice, but extinguished in mice with global *Gipr* deficiency [795,935]. When given centrally, native GIP decreases food intake in mice only when infused at doses of 2–6 nmol/day [937,938], but not when given at lower doses [934,[937], [938], [939]]. Chronic infusion of acyl-GIP into the lateral ventricle of DIO mice, however, robustly decreases body weight and food intake at doses of as little as 0.02 nmol/day, and these effects vanish in mice with Nestin Cre-mediated neuronal loss of *Gipr* [795]*.* Collectively, these data indicate that GIP decreases body weight in obese rodents via centrally mediated inhibition of food intake (Figure 8). These effects depend on functional GIPR signaling in the CNS, but require peripherally administered GIP in supraphysiological doses, or with sustained action by long-acting agonists, to affect body weight and food intake. Although supported by multiple lines of evidence, the observation that native GIP affects food intake only at supraphysiological doses [934,[937], [938], [939]] is tempered by the demonstration that chemogenetically-induced elevation of endogenous GIP to near-physiological (postprandial) levels improves glucose tolerance in lean mice, and also decreases food intake, effects that vanish with peripheral or central administration of GIPR antagonizing antibodies [120]. Many preclinical studies do not report which species and sequence of GIP is used. The GIP system is evolutionary less conserved than the GLP-1 system [388,397,398,406], and hGIP is less potent than rodent GIP and a partial agonist for cAMP production at mouse or rat GIP receptors, irrespective of the dose [564]. The use of human GIP in rodent studies may, hence, even under supraphysiological doses, not yield sufficient occupancy and/or downstream signaling at the brain GIPR to affect feeding. Such suboptimal receptor exposure might be especially deleterious for the central effects of GIP on food intake, given that higher doses of GLP-1 are also required to decrease body weight and food intake relative to improving glycemia. Differential potency at the rodent GIPR, or ligand-specific differences in brain penetrance, might also explain why different long-acting GIPR agonists vary substantially in their required dose to affect feeding, with peripherally applied doses ranging from 30 to 3,000 nmol/kg [580,581,591,795,930,935]. Determination of species-specific receptor affinity, potency and efficacy is hence a crucial, but often neglected, requirement when studying the pharmacological effects of GIPR agonism, and this may have contributed to the discrepant results observed using different GIPR agonists.

GIP is found in the cerebrospinal fluid of mice [939] and humans [940], which along with the demonstration that GIP analogues have neuroprotective effects in animal models of Alzheimer's and Parkinson's disease [926,929,941,942] suggests that GIP is able to cross the blood–brain-barrier (BBB). However, even hydrophilic substances incapable of crossing the BBB can still reach the CSF by traversing the blood-cerebrospinal barrier (BCSF) located in the choroid plexus of the brain ventricles [943]. Low amounts of GIP may also be produced in certain brain areas [85,[121], [122], [123],^606^]. But similar to liraglutide and semaglutide, which do not cross the BBB [[944], [945], [946]], fluorescently-labeled GIP accumulates after single peripheral administration predominantly in the area postrema (AP) and the median eminence (ME), but not in the BBB shielded regions of the hypothalamus or the hindbrain [580,581]. Whilst it is possible that fluorescent labeling affects drug penetrance into the brain, axonal and dendritic projections may nonetheless extend from the hypothalamus to the ME to facilitate contact with circulating hormones [947], and consistent with this, peripheral administration of acyl-GIP increases cFos neuronal activity in various regions of the hypothalamus, including the ARC and the PVH [581,795], although in contrast to GIP-induced cFos labelling in the hindbrain, there is a fairly limited colocalization with *GIPR*-Cre labelled cells [120]. Tanycytes in the ME further facilitate BBB transcytosis of circulating peptides [948], and while this may be crucial for liraglutide to inhibit food intake [949], no such transport has yet been demonstrated for GIP.

Both GLP-1R and GIPR agonists reach the hindbrain via the area postrema (AP) [580,581,944,945], but while GLP-1R agonists induce cFos neuronal activity in the AP and the nucleus tractus solitarius (NTS) [54], most GIPR agonists induce cFos activity primarily in the AP [581,932,950,951]. GIP-induced cFos activation in the AP is preserved in vagotomized mice, indicating that vagal afferents are not required for GIP-induced activation of AP neurons [951]. Expression of *Gipr* is further enriched in the AP [932,950], but scarce in the NTS and the nodose ganglion of the vagus nerve [580,932,950]. In the AP and the hypothalamus, neuronal expression of *Gipr* is largely limited to *Vgat*-expressing inhibitory GABAergic neurons [607,611,952], and specific deletion of *Gipr* in these neurons not only extinguishes GIP-induced cFos activity in the AP, ARC, and PVN, but also renders mice resistant to GIP-induced weight loss and inhibition of food intake [581]. The exact mechanisms by which GIPR signaling in GABAergic neurons decreases body weight and food intake in rodents remains to be determined, along with delineation of whether these effects are mediated via GIPR signaling in the AP, the hypothalamus or both. In any case, there is ample evidence indicating that the AP and the ME are first order signaling nodes that facilitate food intake inhibition through GIPR agonism in rodents (Figure 8). Efferent neurons regulating food intake project from the AP to the NTS, and further to the parabrachial nucleus (PBN) [953], the central amygdala (CeA) and the hypothalamus [580,950,954], all of which are implicated in the regulation of homeostatic and/or hedonic food intake [953,955,956]. Although the neurons in the PBN are primarily glutamatergic, and hence do typically not express *Gipr* [957], GIPR neurons project from the dorsal vagal complex (DVC) to the PBN [580], and peripherally injected acyl-GIP increases cFos activity not only on the AP, but also in the PBN [580], CeA [954] and the hypothalamus [581,587,795]. Chemogenetic activation of GIPR neurons in the DVC is sufficient to induce cFos activity in the PVH, and to reduce food intake in mice [580], suggesting that GIP decreases food intake by transmitting satiety signals from the AP to the PBN, CeA and the hypothalamus. The PVH also receives projections from the ARC, and while peripheral administration of acyl-GIP increases cFos activity in the ARC [581,795], chemogenetic activation of hypothalamic GIPR neurons decreases food intake in mice [587]. Viral-mediated knock-down of *Gipr* in the hypothalamus, however, does not extinguish superiority of GLP-1 and GIP co-therapy to yield greater weight loss and further inhibition of food intake relative to GLP-1R agonism alone [580]. Although these data suggest that hypothalamic GIPR signaling plays only a minor role in regulating food intake, viral-mediated gene knock-out is often incomplete and requires confirmation in genetic KO models. Moreover, the observation that a small fraction (<10%) of POMC neurons express *Gipr* [587], and that single peripheral administration of acyl-GIP increases cFos activity in a comparable subset of POMC neurons in the ARC [591] suggests involvement of the hypothalamus in the body weight lowering and food intake suppressing effects of GIP. Activation of ARC POMC neurons has further been shown after treatment of DIO mice with the long-acting GIPR agonist GIPFA-085 [936], but DIO mice with targeted deletion of *Gipr* in cells/neurons that express the *leptin receptor* (*Lepr*) do not show differences in body weight or food intake and remain fully sensitive to GIP-induced weight loss and inhibition of food intake [591]. Nonetheless, GIP and GLP-1 have recently been shown to inhibit AGRP neuronal activity in fasted mice, and that GIP but not GLP-1 is required for full nutrient-induced silencing of AGRP neuronal activity [958]. In summary, there is increasing evidence indicating that long-acting GIPR agonists act centrally to decrease body weight and food intake in DIO mice via the hindbrain-hypothalamus axis, and these effects are mediated via GIPR signaling in GABAergic neurons, with potential implication on AGRP neurons.

While food intake inhibition through peripherally administered acyl-GIP is extinguished in mice with neuronal loss of *Gipr*, acyl-GIP-induced weight loss is in some studies partially preserved [795]. These data indicate that acyl-GIP not only decreases body weight through inhibition of food intake via CNS GIPR neurons, but potentially also by non-CNS mechanisms that are unrelated to food intake. In agreement with this are recent data showing that mice with adipocyte-specific overexpression of GIPR are lean and show decreased body weight when fed with a HFD [799]. Nonetheless, future clinical studies are needed to examine whether GIP effects on body weight and food intake translate to humans.

Infusion of native hGIP at physiological concentrations (0.8–1.5 pmol/kg/min) for 5 h prior to or during a meal did not affect food intake, energy expenditure or plasma triglyceride removal in healthy humans [959]. In humans living with overweight/obesity, infusion of GIP at a dose of 4–6 pmol/kg/min for 4–5 h even abolished food intake inhibition of simultaneously infused GLP-1 [181], or revealed no effect relative to treatment with GLP-1 alone [960]. However, caution is warranted when extrapolating data from acute studies using native peptides to chronic studies using long-acting peptides that have been optimized for enhanced pharmacokinetic properties. In line with this notion, some studies in DIO mice show that acyl-GIP decreases food intake, at the earliest, after 6–8 h of peripheral bolus administration [581,795]. Moreover, two long-acting, selective GIPR agonists, LY3537021 and NNC0480-0389, were recently tested in healthy humans and in individuals with T2D. In a 34 week, phase 2 study in individuals with T2D, NNC0480-0389 lowered body weight by −4.3% compared to −10% in individuals receiving semaglutide and −2.7% in participants receiving placebo (EudraCT Number 2020-004863-14 - Clinical trial results - EU Clinical Trials Register). While the numerical weight loss seen with NNC0480-0389 did not reach statistical significance compared to placebo, two of the four tested doses of NNC0480-0389 in combination with semaglutide 2.4 mg resulted in significantly greater weight losses relative to semaglutide 2.4 mg alone; supporting a weight loss effect of the GIPRA. In a 4-week, phase 1 study, LY3537021 showed a placebo-adjusted decrease in body weight of up to −1.79 kg in healthy volunteers, and up to −2.78 kg in participants with T2D [961]. These data collectively suggest that the body weight lowering effects of GIPR agonism may to some extent translate from rodents into humans.

### 17.2 Use of GIPR agonists in unimolecular formats with GLP-1R agonists for the treatment of obesity and diabetes

With the spotlight on therapeutic exploitation of GLP-1 mimetics, plus doubts about effectiveness of GIPR agonism in humans with T2D, early preclinical studies with stable GIPR agonists had until very recently [961] not progressed to clinical development. However, in 2013, Matthias Tschöp and Richard DiMarchi reported the first unimolecular peptide co-agonists with similar affinities, efficacies and functional cAMP activity at the receptors for GIP and GLP-1 [177]. The rationale for the development of these GIPR:GLP-1R co-agonists was based not only on the assumption that the molecules would act at both target receptors to further improve glucose metabolism, as demonstrated earlier in ob/ob mice [287], but also on the then rather unexpected observation that co-therapy with both peptides yielded greater weight loss and further inhibition of food intake relative to GLP-1R agonism alone in DIO rodents [177]. The first GIPR:GLP-1R co-agonists were based on the glucagon sequence, in which amino acid residues from GLP-1, GIP and exendin-4 were stepwise introduced to achieve near-balanced agonistic activity at GIPR and GLP-1R, while also protecting against DPP4 proteolysis and abolishing activity at the glucagon receptor (GCGR) [177].

The first generation of these DPP4 protected co-agonists was either pegylated or acylated with a C16 fatty acid, and exhibited superiority over liraglutide to decrease body weight and to improve glycemia in DIO and db/db mice, ZDF rats and cynomolgus monkeys [177]. The acylated molecule (known as MAR709, NN9709, RG7697, RO6811135 or NN0090-2746) advanced to clinical development, but despite good tolerability and meaningful reduction in body weight and HbA1c in phase 1 trials [177,962,963], development was discontinued after a short phase 2a study, in which MAR709 showed, at the single tested dose, only moderate superiority over open-label liraglutide in people with T2D after 12-weeks of treatment [964]. While the short duration of treatment and the lack of a dose-escalation regime were distinct limitations of this clinical study [964], in DIO mice, MAR709 caused greater weight loss and further inhibition of food intake relative to treatment with a pharmacokinetically-matched GLP-1 backbone, and this superiority was extinguished in mice lacking GIPR in either the CNS [795] or more specifically in GABAergic neurons [581].

The GIPR:GLP-1R co-agonist tirzepatide (previously known as LY3298176) was first reported by Eli Lilly in 2018 [176]. The DPP4-protected molecule is based on the sequence of GIP, and contains a mixture of residues from GLP-1, GIP, and exendin-4. Tirzepatide has similar affinity and potency for cAMP production at the GIPR as native GIP, and 5-fold lower affinity and 13-fold lower potency for cAMP production at the GLP-1R compared to native GLP-1 in recombinant human cell lines overexpressing these receptors. The molecule has a C20 fatty diacid attached to render the molecule suitable for once-weekly (QW) dosing [176]. Although MAR709 and tirzepatide are potent full agonists at hGIPR, MAR709 maintained high potency at mGIPR, while tirzepatide has at least 10-fold lower potency at mGIPR. Consequentially, at a dose of 10 nmol/kg, MAR709 depends on CNS GIPR signaling to yield superior weight loss over GLP-1R agonism in DIO mice [581,795], whereas tirzepatide, when used at the same dose, does not decrease body weight in GLP-1R KO mice [965]. Nonetheless, at a dose of 10 nmol/kg, tirzepatide improves insulin sensitivity independent of body weight loss via GIPR [965], suggesting that tirzepatide at this dose has sufficient occupancy of mGIPR to affect glycemia but not body weight. Consistent with this are recent observations showing that the body weight lowering effect of tirzepatide is further enhanced in DIO mice when tirzepatide is given together with a long-acting GIPR agonist [966]. In another study, however, tirzepatide was shown to stimulate insulin secretion in isolated human islets predominantly via GIPR, but almost exclusively via GLP-1R in murine islets [967]. The species-specific differences in tirzepatide's potency at the murine vs. human GIPR warrant caution when extrapolating tirzepatide's metabolic effects from mice to humans. At the GLP-1R, tirzepatide and MAR709 further show notable differences to GLP-1, with both exhibiting different degrees of biased agonism at GLP-1R to favor cAMP production over β-arrestin recruitment relative to GLP-1, and delayed receptor internalization [548,549] and, in case of MAR709, enhanced receptor recycling [548]. At both of its target receptors, tirzepatide is further biased towards pERK1/2 relative to cAMP accumulation when compared to the respective endogenous peptide agonists GLP-1 and GIP [968]. While it remains to be determined whether this biased agonism at the GLP-1R affects drug efficacy, depletion of β-arrestin in GIPR/GLP-1R expressing HEK293 cells increases ligand-induced cAMP/PKA signaling [555]. Tirzepatide and GIP further differentiate in their ability to induce phosphorylation of the human GIPR, with both peptides inducing phosphorylation of four serine residues at hGIPR and tirzepatide inducing an additional 5th serine residue phosphorylation [969].

Tirzepatide received FDA approval for the management of T2D in 2022, and for obesity in 2023. The glycemic effects of tirzepatide (at doses of 5, 10, or 15 mg QW) in individuals living with T2D and/or overweight/obesity were evaluated in the SURPASS 1-6 trials. In SURPASS-1, depending on the dose, up to 92% of participants receiving tirzepatide achieved an HbA1c below 7.0% after 40 weeks of treatment, compared to 19% in placebo treated controls, while up to 52% versus 1% achieved an HbA1c reduction below 5.7% [970]. Tirzepatide performed equally well in the SURPASS 2-6 trials, with superior reductions in HbA1c relative to treatment with semaglutide 1 mg [178], insulin degludec [971], insulin glargine [972,973], and insulin lispro [974], and with preserved efficacy and safety in individuals at risk for CV diseases [973]. In Japanese people living with T2D, tirzepatide decreased HbA1c by −2.8% after 52 weeks of treatment, relative to −1.3% in dulaglutide treated controls [975], and similar effects were observed in the SURPASS J-combo trial, in which tirzepatide was given as add-on therapy to sulfonylureas, biguanides, α-glucosidase inhibitors, thiazolidinedione, glinides, or SGLT2 inhibitors in Japanese people with poorly controlled T2D [976].

The multi-center SURMOUNT trials assessed the efficacy of tirzepatide for weight loss in people living with overweight and one or more complications or obesity. In SURMOUNT-1, tirzepatide led to a reduction in body weight after 72 weeks of treatment of up to −20.9% in non-diabetic individuals, compared to −3.1% in the placebo treated-controls [977]. In people with overweight/obesity and T2D, tirzepatide decreased body weight after 72 weeks treatment by −14.7% relative to −3.2% in placebo controls [978]. Although with minor variations, this level of weight loss is broadly confirmed across the SURPASS trials [178,[970], [971], [972], [973], [974], [975], [976],^979^]. Moreover, tirzepatide reduced waist circumference, systolic and diastolic blood pressure, fasting insulin level, and lipid levels and more than 95% of the participants with prediabetes went into remission to normoglycemia, which seems to have additional beneficial effects on the prevention of type 2 diabetes [977,980]. In SURMOUNT-3, tirzepatide's effects were evaluated in people with overweight/obesity without T2D following intensive lifestyle intervention, demonstrating a reduction in body weight of −18.4% after 72 weeks with tirzepatide, compared to a +2.5% weight gain with placebo [981]. In SURMOUNT-4, individuals living with overweight/obesity were treated with tirzepatide for 36 weeks, followed by continuation with tirzepatide or switch to placebo for an additional 56 weeks [982]. After 36 weeks, the mean percent weight change was −20.9%. Individuals continued on tirzepatide showed an additional weight loss of −5.5%, for a total weight loss of −26.4%, while individuals switched to placebo gained 14.0% during the 56 week follow-up period [982]. In summary, along with the recent observation that the body weight lowering effects of GIP may to some extend translate to humans [961], GIPR:GLP-1R co-agonism has emerged as a valuable and highly effective strategy for the management of obesity and T2D, and with comparable safety relative to best-in-class GLP-1R agonists [178,[970], [971], [972], [973], [974], [975], [976],^979^,^983^]. Notably, a head to head comparison of the efficacy of tirzepatide and semaglutide at their highest approved doses demonstrated a mean weight loss of 20.2% vs 13.2% for tirzepatide vs. semaglutide, respectively (https://investor.lilly.com/news-releases/news-release-details/lillys-zepboundr-tirzepatide-superior-wegovyr-semaglutide-head#:∼:text=Lilly's%20Zepbound%C2%AE%20(tirzepatide)%20superior,13.7%25%20%7C%20Eli%20Lilly%20and%20Company). Consistent with these findings, individual trials assessing each drug alone show that tirzepatide at the highest approved dose of 15 mg QW outperforms semaglutide 2.4 mg QW to yield greater weight loss and further improvement of glucose control [971,977,978,984,985], and this has also been confirmed in a recent meta-analysis [986]. Comparing drug efficacies nonetheless remains challenging, since data related to receptor exposure in key target tissues are unavailable, and no human study has yet assessed tirzepatide effects upon blockade of GLP-1R. While the relative contribution of GIPR agonism to the metabolic efficacy of tirzepatide remains to be demonstrated, recent clinical data with two long-acting, selective GIPR agonists, LY3537021 and NNC0480-0389, support a potential body weight lowering effect of GIPR agonism in humans (see above).

The therapeutic potential of GIPR:GLP-1R co-agonism extends beyond managing T2D and obesity. A recent phase 2 study involving individuals with biopsy-confirmed metabolic-associated steatohepatitis (MASH) and moderate to severe fibrosis demonstrated that up to 62% of participants treated with tirzepatide achieved MASH resolution without worsening fibrosis, compared to only 10% in the placebo group after 52 weeks of treatment [987]. Additionally, 51% of individuals treated with tirzepatide showed an improvement of at least one fibrosis stage, compared to 30% of participants in the placebo group [987]. In a phase 3 trial involving individuals with moderate-to-severe obstructive sleep apnea and obesity, tirzepatide decreased the number of apneas and hypopneas during an hour of sleep (apnea-hypopnea index, AHI) by −29.3 events per hour, relative to −5.5 events in placebo controls [988]. Along with further decreases in patient-reported sleep impairment and disturbance, high-sensitivity C-reactive protein (hsCRP) levels and systolic blood pressure [988], these data prompted the FDA on December 20th 2024 to approve tirzepatide for the treatment of moderate-to-severe obstructive sleep apnea and obesity in adults. A recent clinical study assessing measured and patient self-reported alcohol drinking behavior further showed that individuals treated with tirzepatide (or semaglutide) for ≥30 days exhibit reduced alcohol cravings, desire to drink alcohol, and binge drinking episodes, which along with decreased Alcohol Use Disorders Identification Test (AUDIT) scores indicates that these medications may hold therapeutic value for also treatment of addictive behavior [989]. Whether this holds true for also drug addiction and nicotine use disorder, however, warrants further clarification.

### 17.3 Use of GIPR:GLP-1R:GCGR triple agonists for the treatment of obesity and diabetes

Expanding the concept of unimolecular incretin-based polyagonism, Tschöp and DiMarchi reported in 2015 the first acylated peptide with 10-fold higher potency for cAMP accumulation than the respective endogenous agonist at the receptors for GLP-1, GIP and glucagon [295]. The rational for adding glucagon receptor (GCGR) action to GIPR:GLP-1R co-agonism was anchored on the preclinical success of GLP-1R:GCGR agonism, which yielded superior weight loss with preserved (or even improved) glycemic benefits relative to GLP-1R agonism in obese rodents [294]. In keeping with glucagon's complementary pharmacology to decrease food intake, increase energy expenditure and enhance hepatic lipid utilization [745,990,991], the triagonist outperformed GIPR:GLP-1R co-agonism to further decrease body weight while improving glucose control and hepatic lipid metabolism in rodents [295]. The functional importance of each peptide entity was verified by genetic or pharmacological silencing of each individual biological activity [295], and was further evident by the classical glucagon effects to increase energy expenditure and hepatic lipid utilization [295,992]. The triagonist further improved dyslipidemia and ameliorated hepatic steatosis in obese mice, and these effects were observed even at doses where the drug had only marginal effects on body weight and food intake [993]. A series of GIPR:GLP-1R:GCGR triagonists were additionally developed, each with notable differences in structure, duration of action, and activity at each target receptor [292,293,[994], [995], [996], [997], [998]]. The clinically most advanced triagonist is retatrutide (LY3437943), which is based on the GIP sequence with stepwise amino acid substitutions introduced to achieve triple agonism [176,997]. The molecule further includes non-natural amino acids at positions 2, 13, and 20, to protect from DPP4 recognition and to enhance the activity at the receptors for GIP and glucagon, while maintaining activity at the GLP-1R, and a C20 fatty-diacid was attached to lysine at position 17 to extend the circulating half-life through albumin binding. In recombinant human cell lines, retatrutide exhibits similar activity at GCGR and GLP-1R (∼2–3 fold lower potency relative to GCG and GLP-1, respectively), but ∼9-fold enhanced potency at GIPR relative to GIP [997]. In obese rodents, it decreases body weight with superior efficacy relative to treatment with tirzepatide [176]. In line with the thermogenic action of GCGR agonism, retatrutide increased energy expenditure, and this effect contributed 30–35% of the total weight loss in DIO mice and disappeared upon pharmacological inhibition of GCGR [997]. In Phase 1 clinical trials, retatrutide exhibited a safety profile comparable to dulaglutide [997,999]. Treatment-emergent adverse events (TEAEs) were mainly gastrointestinal and occurred in 63%, 60%, and 54% of participants treated with retatrutide, dulaglutide, and placebo, respectively [999]. Retatrutide dose-dependently decreased systolic/diastolic blood pressure by up to 12/2 mmHg, while mean pulse rate increased by up to +10 bpm during the last four weeks of treatment [999]. In phase 2 trials, retatrutide decreased body weight in individuals living with overweight/obesity after 48 weeks of treatment, and at the highest tested dose of 12 mg QW, by −24.2%, relative to −2.1% in placebo-treated controls, and with 26% of patients achieving weight loss >30% compared to 0% for placebo [1000]. Weight loss was associated with a decrease in plasma triglycerides and LDL cholesterol, and although heart rate dose-dependently increased by +7 bpm at week 24 when corrected for placebo, it declined to +5.7 by week 48 [1000]. Retatrutide also caused a dose-dependent decrease in liver fat, with up to 86% of individuals with metabolic dysfunction associated fatty liver disease reaching normal liver fat after 48 weeks of treatment [1001]. In individuals with T2D, retatrutide resulted in a decrease in HbA1c of up to −2.02% compared to −0.01% for placebo and −1.41% for the selective GLP-1R agonist dulaglutide, demonstrating that in the context of GLP-1R and GIPR agonism, activation of glucagon receptors does not impair glycemic control [1002]. In summary, incretin-based polyagonism has emerged as a powerful approach to extend the pharmacological potential of GLP1-1R agonism, and with favorable tolerance and translation of metabolic effects from rodents to humans.

Despite such major strides in the development and clinical use of these unimolecular polyagonists which target the GIPR, a number of stable GIPR monoagonist peptides are in preclinical development or phase 1 or 2 clinical trials. These include the yet unpublished XW-017 (Hangzhou Sciwind Biosciences Co), ZP6590 (Zealand) and several long-acting GIPR agonists, including NNC0480-0389 (Novo Nordisk), LY3537021 (Eli Lilly) [961], LY3532226 (Eli Lilly) and GIPFA-085 (Eli Lilly) [936]. Beneficial effects of these agents have been noted in conference presentations and most recently for LY3537021, which is reported to induce significant weight loss in subjects with T2D but without nausea or vomiting [961], and NNC0480-0389, which in a phase 2 study in individuals with overweight/obesity and T2D enhanced weight loss relative to treatment with semaglutide 2.4 mg (EudraCT Number 2020-004863-14 - Clinical trial results - EU Clinical Trials Register). It is evident therefore that these GIP analogues can overcome the resistance reported when administering native GIP [144,151,152]. Nevertheless, it seems questionable whether any of these monoagonists in development will, when administered alone, prove superior to the clinical benefits of the established polyagonist peptides. One advantage could be less severe GI-side effects, greater compliance and fewer discontinuations compared with GLP-1 agonism [1003]. Another possibility could be simple co-administration of these agents with long-acting analogues of other therapeutic peptides as advocated for CCK-8, GLP-1, OXM and PYY [1004,1005]. However, even then it might prove more attractive to develop appropriate unimolecular polyagonists and a GIP/xenin hybrid has already been shown to be effective in DIO rodents [440,1006]. Interestingly, dual GIP/GLP-2 agonists have also been developed for potential treatment of bone fragility and osteoporosis [819,847]. In contrast to peptide therapeutics, there has been little success in attempts to develop small molecule agonists acting at the GIPR. One exception is 4-hydroxybenzoic acid 2-bromobenzylidene hydrazide (4H2BH) which appears to antagonize both GIP and glucagon action [1007].

### 17.4 Regulation of body weight and food intake, insulin action by GIPR antagonism and by unimolecular GIPR antagonism/GLP-1R agonism

The benefits of GIPR agonism can largely be attributed to weight loss and stimulation of insulin secretion [281,581,591,795]. It is therefore counterintuitive that GIPR signal inhibition also shows clear benefit in obesity and T2D. These include protection of *Gipr* deficient mice from diet-induced obesity and associated glucose intolerance [800,[804], [805], [806],[1008], [1009], [1010]], and amelioration of obesity and glucose intolerance in ob/ob and/or DIO mice by low molecular weight GIPR antagonists [1011,1012], Pro(3)GIP analogues [272,[1013], [1014], [1015]], active or passive GIP vaccination [[1016], [1017], [1018], [1019]], or targeted destruction of GIP-secreting K-cells [439]. Moreover, genetic *GIPR* variants that lead to decreased receptor function are associated with decreased BMI in humans [560,856,1020,1021]. The most extensively studied scenario concerns the antidiabetic effects of Pro(3)GIP described by the Coleraine group where, unlike GIPR agonists, amelioration of diabetes is associated with lower rather than raised insulin concentrations [272,280]. This is attributed to compromised effects of native GIP on adipose tissue resulting in clearance of triglyceride from adipose and liver stores, thereby substantially improving insulin sensitivity and alleviating insulin resistance. In such circumstances, insulin demand is less and any deficit in GIP action from the enteroinsular axis is not missed. As discussed later, an alternative view is that GIPR antagonism may result in the promotion of GLP-1 action. However, that does not necessarily fit with phenotype induced in these animal models where the major effect is to improve insulin action. Such action is not restricted to these scenarios or Pro(3)GIP and later studies published in 2015 using more specific GIPR antagonists, GIP(3-30)Cex-K(40)[PAL] and Pro(3)GIP(3-30)Cex-K(40)[PAL], similarly ameliorated insulin resistance and glucose intolerance in both DIO and db/db mice [169,1022]. However, how GIPR inhibition protects from obesity and associated insulin resistance remains puzzling, and may include increased lipid utilization [804], decreased intestinal nutrient uptake [1023], decreased food intake [805,1008], and/or increased energy expenditure [803,805]. In support of this, a recent study showed that the GIPR antagonist AT-7687 prevented weight gain when given as monotherapy, in HFD-fed non-human primates [1024]. Although mice with adipose-specific loss of *Gipr* are not protected from diet-induced obesity [589,807,808], isoproterenol-induced lipolysis is increased in isolated GIPR deficient white adipocytes [803]. Nonetheless, oral administration of lipids increases lipid storage in global *Gipr* deficient mice [803], which is in line with the observation that GIP induces lipolysis under conditions where insulin action is low to absent [597,598,769,[794], [795], [796]], and that mice with adipocyte-specific overexpression of *Gipr* show decreased fat mass and protection from diet-induced obesity [799].

In contrast to mice with adipose-specific loss of *Gipr* [589,807], mice with Nestin Cre-mediated neuronal loss of *Gipr* [795], or with deletion of *Gipr* specifically in inhibitory GABAergic neurons [581,966], show decreased body weight and fat mass when fed with a HFD, and this is paralleled by decreased food intake without changes in energy expenditure. However, although these data indicate that protection from diet-induced obesity in *Gipr* deficient mice originates, at least in part, from the lack of GIPR signaling in the CNS, the obesity-protecting phenotype of mice with neuronal loss of *Gipr* is rather mild and does not fully explain the resistance to weight gain seen after global *Gipr* deletion.

Protection from diet-induced obesity is paradoxically also observed in some studies with GLP-1R deficient mice [805,806]. Furthermore, chronic antagonization of GLP-1R decreases body weight gain during HFD-feeding with equal efficacy relative to antagonization of GIPR, and body weight gain is even further reduced upon adjunct antagonization of both incretin receptors [806]. Antagonism of either GIPR or GLP-1R enhances the sensitivity to the alternate incretin, as demonstrated *in vivo* by enhanced glycemic action of GIP following inhibition of GLP-1R [806] and by enhanced insulinotropic action of GLP-1 in the perfused GIPR-deficient pancreas [1025]. Other studies, however, show that genetic loss of either GIPR or GLP-1R does not enhance the insulin response to and plasma concentrations of the alternate incretin after oral glucose administration [[1026], [1027], [1028]]. Nonetheless, consistent with the potential anti-obesity effect of GIPR signal inhibition, evident from early studies, are more recent reports showing that certain GIPR antagonists, or GIPR neutralizing antibodies, prevent HFD-induced weight gain, improve insulin sensitivity, and/or decrease body weight and food intake in DIO mice [939,1029,1030] and non-human primates [1024,1029], particularly when given in adjunct to GLP-1R agonism [1029,1031,1032].

AMG133 (maridebart cafraglutide, maritide, Amgen) is a bispecific hybrid that comprises two GLP-1R agonists moieties conjugated to a monoclonal anti-GIPR antagonist [1032,1033]. In DIO mice and non-human primates, AMG133 decreases body weight with superior efficacy relative to treatment with the respective monotherapies alone, and in DIO mice, drug-induced weight loss is superior to treatment with dulaglutide [1032]. Weight loss induced by AMG133 is primarily driven by inhibition of food intake, and is paralleled by improved insulin sensitivity, a decrease in blood glucose and a decrease in plasma levels of insulin, triglycerides and cholesterol [1032,1033]. In phase 1 trials, AMG133 was well tolerated, and a once monthly injection schedule over three months led to more than 10% weight loss in healthy human subjects [1033]. Although the relative contributions of blocking the GIPR vs. activating the GLP-1R cannot be ascertained in these clinical studies, these data have encouraged progression to phase 2 development. These data were recently corroborated by a study in non-human primates, showing that the peptide-based GIPR antagonist AT-7687 in combination with the GLP-1R agonist liraglutide results in greater weight loss relative to placebo as compared to treatment with liraglutide alone [1024].

The mechanisms of how GIPR antagonism decreases body weight and food intake remain elusive. Central immunoneutralization of GIPR was recently shown to decrease SOCS3, a negative regulator of leptin signaling, while central GIPR agonism had the opposite effect, hence indicating that GIPR antagonism may decrease body weight by improving leptin sensitivity [939]. Improvement of leptin sensitivity can, however, not fully explain protection from obesity in *Gipr* deficient mice, since lack of GIPR decreases body weight in obese leptin deficient ob/ob mice [804]. Mice with specific loss of *Gipr* in *Lepr* neurons are further not protected from diet-induced leptin resistance [591]. Another popular hypothesis is that GIPR agonism desensitizes the GIP receptor, and hence leads to functional antagonism [1034]. However, this is not supported by distinct differences in the metabolic phenotype induced by GIPR agonism vs. antagonism in various animal models of obesity-diabetes, with the latter intervention alleviating insulin resistance and inducing β-cell rest [169,272,1014,1015,1022]. The hypothesis, however, is anchored on the observation that repetitive GIPR stimulation decreases the sensitivity of the GIP receptor in DIO mice and in isolated adipocytes [592], and is supported by the phenotype of the common naturally occurring GIPR variant E354Q (rs1800437) with enhanced internalization rate and altered intracellular sorting [[869], [870], [871]], resulting in overall loss-of-function phenotypes [820,1035,1036]. Desensitization of GIPR by GIPR agonism, however, seems to be restricted to GIP effects on the islets, and does not occur for GIP's effect on CNS-mediated food intake suppression [1037]. Ligand-induced receptor desensitization has also been shown for GLP-1 in rat insulinoma INS-1 cells [617] and for both incretins in hamster β-cell HIT-T15 cells [616], but is not observed *in vivo* in healthy human subjects [158]. Here, it is, however, important to note that the desensitization of the GIPR is different from that of the GLP-1R, as GIPR internalization relies on arrestins [559], while that is not the case for the GLP-1R [558]. The same pattern goes for the cAMP production elicited by these two receptors, as the GIP receptor's Gs-mediated signaling is weakened in the absence of arrestins, while the opposite is observed for the GLP-1R [856]. Thus, from a molecular and cellular perspective, these two incretins are distinct when it comes to long-term agonist exposure. Prolonged treatment of DIO mice with acyl-GIP does, however, not decrease expression of *Gipr* in either the hypothalamus or the adipose tissue [795]. Yet, this could be due to a generally impairment of mouse GIPR in arrestin recruitment and receptor internalization compared to the human GIPR [562]. GIPR agonists and antagonists may also affect systemic energy metabolism via different central and/or peripheral mechanisms. While GIPR agonists depend on GABAergic GIPR neurons in the CNS to decrease food intake [581,966], GIPR antagonists may silence these or other neuronal populations to enhance the anorectic action of adjacent glutamatergic neurons. In line with such an assumption is the demonstration that a significant portion of food-intake inhibiting GLP-1R neurons are glutamatergic [612], and that GIPR antagonists primarily decrease food intake when given in adjunct to GLP-1 [1029,1031].

### 17.5 GIP effects on drug-induced emesis

Gastrointestinal (GI) discomfort, such as constipation, diarrhea, nausea and emesis are common adverse effects associated with the use of numerous pharmacotherapies, including antibiotics, antidepressants, opioids, chemo- or radiotherapy and GLP-1R agonists. Related to GLP-1R agonists, GI-adverse effects may occur in >50% of people at treatment initiation and represent the most frequent reasons for temporary or permanent treatment discontinuation [[1038], [1039], [1040], [1041]]. Although GI-side effects associated with the use of GLP-1R agonists are often for the majority of patients reported as transient and often resolve after 4–6 weeks of treatment without permanent drug withdrawal [[1038], [1039], [1040], [1041]], they decrease the quality of life of the affected individuals and may prevent the use of higher doses otherwise required to optimize treatment outcome. Appreciably, GIPR agonism attenuates the emetic effects of GLP-1R agonism, as demonstrated by amelioration of GLP-1 induced pica behavior in rats, conditioned taste avoidance in mice and emesis in musk shrews [930] (Figure 8). In all of these animal models, the co-therapy of long-acting GIPR agonists and GLP-1R agonists reduced indices of malaise observed with GLP-1R agonism alone. GIP also decreases conditioned taste avoidance induced by treatment with PYY in mice [932] and attenuates chemotherapy-induced nausea and vomiting in ferrets and shrews [931]. Recent clinical studies show that the selective, long-acting GIPR agonists attenuate GI adverse events of GLP-1R agonists, providing evidence that anti-aversive effects of GIP may translate to humans [1003,1042]. The mechanisms by which GIPR agonism exerts its anti-emetic effects are not well understood but seem to reside in its ability to act on the hindbrain GIP receptor [1043].

Drug-induced emesis is for the most part mediated via the dorsal vagal complex (DVC), which comprises the AP, the NTS and the DMV [[1043], [1044], [1045], [1046]], hindbrain areas also implicated the regulation of body weight and satiety by the incretin hormones. Similar to the incretins, many emetic drugs elicit cFos neuronal activation in the AP and the NTS [[1047], [1048], [1049], [1050]] and ablation of the AP in cats or dogs abolishes emesis induced by radiation- and chemotherapy [1051,1052] nicotine [1053], PYY [1054], and apomorphine [1052]. GIP further suppresses conditioned flavor avoidance in mice by inhibiting GDF15/GFRAL responsive excitatory neurons in the AP, and these effects vanish when *Gipr* is deleted in a specific cluster of AP inhibitory neurons [951]. In the NTS, the emetic chemotherapeutic cisplatin induces cFos activity in GLP-1R neurons, and blockade of these neurons using the GLP-1R antagonist exendin 9-39 attenuates cisplatin-induced anorexia, weight loss and pica in rats [1047]. Pharmacological blockade of GLP-1R further attenuates lithium chloride (LiCl)-induced cFos activation in the AP, NTS, and PBN [1055] and further attenuates LiCl-induced dopamine suppression in the VTA [1056]. The emetic effect of PYY is paralleled by increased cFos activation in the PBN and GIP treatment decreases PYY-induced cFos activation in this area [932]. In line with a role of hindbrain GIPR signaling in the anti-emetic effect of GIPR agonism is the observation that central (i.c.v.) administration of GIP into the 4th ventricle of rats decreases kaolin intake induced by systemic GLP-1 treatment in rats [930], and that GIPR agonism attenuates GLP-1-induced cFos neuronal activation in the AP and NTS of rats and shrews [930].

Notably, the anorectic and aversive effects of GLP-1R agonism seem to be mediated by a heterogenous group of neurotransmitter/neuropeptide-expressing neurons in the DVC. Of relevance to GLP-1-mediated effects on nausea and malaise, it is interesting to note that GLP-1R expressed on a subset of CCK/glutamatergic neurons in the AP and NTS co-express the receptor complex Gfral-Ret for the emetogenic cytokine GDF15. Although these neurons are not directly targeted by GIPR agonism (as they do not co-express the GIPR), GIP ameliorates GLP-1-induced conditioned taste avoidance in mice, and this is paralleled by reduced GLP-1 induction of cFos activity in these CCK/glutamatergic neurons [950]. The antiemetic effect of GIPR agonism is undoubtedly an appreciable merit, which may have contributed to the near comparable tolerability of tirzepatide and semaglutide at the highest approved doses, i.e. 15 mg for tirzepatide versus 2.4 mg for semaglutide.

## 18 GIP effects on neurodegenerative diseases

The first data indicating that GIPR agonism has neuroprotective effects in the brain date back to 2005, where studies in rats showed that GIP stimulates proliferation of hippocampal progenitor cells *in vitro* and *in vivo* [122,1057]. The rate of progenitor cell proliferation in the hippocampal dentate gyrus correlates positively with the expression of *Gipr* in the hippocampus [122], and is accordingly decreased in *Gipr* deficient mice [122,925]. Treatment with [D-Ala_2_]GIP further improves mitochondrial function while decreasing autophagy and apoptosis in human neuroblastoma SH-SY5Y cells exposed to rotenone, a pesticide and inhibitor of complex 1 of the mitochondrial electron transfer chain, that can induce Parkinson's disease (PD) in humans [1058]. In line with these data, GIP promotes neuronal survival in cultured cerebellar granule neurons by inhibiting apoptosis [606,1059], and mice deficient for *Gipr* show impaired learning and memory, along with reduced synaptic plasticity in the hippocampus [925], a key region that is implicated in spatial learning and memory [1060,1061] and involved in the development of neurodegenerative diseases [929]. Conversely, transgenic mice overexpressing *Gipr* show enhanced exploratory behavior, increased motor performance and improved working memory [1062], while treatment of wildtype mice or rats with [D-Ala_2_]GIP enhances learning, synaptic plasticity and neurogenesis in the hippocampus [923,1063]. In line with these data, central (icv) administration of GIP prevents spatial memory impairments induced by icv infusion of β−amyloid oligomers [1064]. Expression of *Gipr* further increases after nerve injury in the lumbar dorsal root ganglia [1065] and spinal cord [1066] which, along with the observation that regeneration of the crushed sciatic nerve is impaired in *Gipr* deficient [1065], implies that GIPR agonism may promote axonal repair and regeneration after neuronal injury. In 12 month old APP/PS1 mice, a transgenic mouse model for AD [284,1067], treatment with [D-Ala_2_]GIP protects from impairment of learning and memory, and these effects are paralleled by reduced synaptic loss, preservation of synaptic plasticity, decreased amyloid plaque formation and amelioration of inflammation, oxidative stress, and DNA damage [1068,1069]. The beneficial effects of [D-Ala_2_]GIP to decrease synaptic loss and inflammation are preserved in 19 month old APP/PS1 mice [1070], suggesting that GIPR agonism may be beneficial even in more advanced stages of the disease. GIPR agonism also decreases central inflammation in aged wildtype mice, and improves synaptic plasticity in the hippocampus of aged wildtype and APP/PS1 mice, hence indicating that GIPR agonism ameliorates the disease- and age-related loss of synapses [1070]. In APP/PS1 mice, GIP-induced improvement of learning and memory is paralleled by enhanced *in vivo* long-term potentiation (LTP) in the hippocampus, reduced brain β−amyloid deposition, lower astrocyte and microglia activation and decreased expression of IL-1β, TNF*α* and NF-kB [1071]. In agreement with this, GIPR agonism reduces in APP/PS1 mice the number of β−amyloid plaques, central inflammation and ER stress, while preventing the decline in neuronal progenitor cell proliferation and cognitive impairment [941,1068].

Neuroprotective effects of GIPR agonism have also been shown in animal models of Parkinson's Disease (PD). In mice treated with 1-methyl-4-phenyl-1, 2, 3, 6-tetrahydropyridine (MPTP), a chemical that blocks mitochondrial activity and induces Parkinson-like symptoms in humans [1072], [D-Ala_2_]GIP improves motor activity, protects synapses in the substantia nigra from the MPTP-induced toxicity, decreases central inflammation and normalizes cAMP/PKA/CREB signaling in the substantia nigra [1073]. The latter is consistent with the observation that [D-Ala_2_]GIP increases cAMP/PKA/CREB signaling in the hippocampus of APP/PS1 mice [1071]. Normalization of motor activity and protection of dopaminergic neurons is further observed after GIP treatment in mice subjected to low dose MPTO treatment, a treatment regime that is considered to more realistically mimic PD in mice [1074]. In this study, [D-Ala_2_]GIP reduced chronic inflammation, oxidative stress and levels of lipid peroxidation, while increasing the expression of brain-derived neurotrophic factor (BDNF) [1074], a key central growth factor implicated in neuronal protection [[1075], [1076], [1077]]. GIP protection against MPTP toxicity has also been shown in other studies, along with the demonstration that this effect can be antagonized using (Pro^3^)GIP [1078]. Reduction of oxidative stress by GIPR agonism is paralleled by decreased central levels of malondialdehyde and dopamine, and increased levels of glutathione, hence markers indicative of improved oxidative stress control [1078]. In rats centrally treated with 6-OHDA, a toxin that selectively destroys dopaminergic neurons, continuous infusion of GIP reduces 6-OHDA toxicity and improves motor impairments [1079].

A minor neuroprotective effect was also recently shown for tirzepatide in APP/PS1 mice [1080], and in human neuroblastoma SHSY5Y cells, where tirzepatide stimulated the expression of biomarkers indicative of neuronal growth (CREB, BDNF) and differentiation (MAP2, GAP43) while decreasing the expression of markers indicative of apoptosis (BCL-2 and BAX) [1081]. Unfortunately, however, this study did not assess the effects of GLP-1R agonism alone [1081], which seems crucial given that some studies show GLP-1R agonism to exhibit neuroprotective effects in patients with AD or PD [[1082], [1083], [1084], [1085], [1086], [1087], [1088]]. Long-term treatment with liraglutide, however, did not affect β-amyloid plaque load in two transgenic mouse models for AD [1089], and tirzepatide was recently shown to not improve brain function in 5XFAD and APP/PS1 mice [1090]. It warrants clarification whether limitations of liraglutide and tirzepatide to cross the blood–brain barrier may have constituted a limitation of these studies [1091]. Other GIPR:GLP-1R co-agonists evaluated for their effects in animal models of AD or PD include DA1-JC, a C16 fatty acid acetylated co-agonist [177], DA3-CH, the non-acylated version of DA1-JC, as well as DA4-JC and DA5-CH, which are further optimized for enhanced BBB permeability [946,1091,1092]. In SH-SY5Y cells, DA1-JC decreases rotenone-induced cellular stress with greater significance over placebo relative to GLP-1R or GIPR agonism alone, but without significance of DA1-JC over the respective receptor monoagonists [1058]. DA1-JC also protects from MPTP-induced toxicity in mice [1093,1094] and from 6-OHDA toxicity in rats [1095,1096], and these effects are paralleled by reduced motor impairments, increased neuronal protection, and/or decreased central inflammation [[1093], [1094], [1095], [1096]], albeit without superiority of DA1-JC to GLP-1R or GIPR monoagonist controls [1073,1097]. Similar neuroprotective effects are observed using DA3-CH in APP/PS1 mice [1098], and in MPTP treated mice, DA3-CH shows superiority to liraglutide to reverse MPTP-induced motor impairment, neuronal damage and activation of microglia and astrocytes [1099]. In mice treated with MPTP [1092,1100], or rats treated with 6-OHDA [1101,1102], GIPR:GLP-1R co-agonists optimized for BBB penetrance (DA4-JC and/or DA5-CH) show greater dopaminergic neuron protection and decreased inflammation and/or apoptosis relative to liraglutide, semaglutide or the less BBB permeable co-agonist DA1-JC. Similar beneficial effects of DA4-JC on motor performance and/or inflammation are observed in APP/PS1 mice, in which DA4-JC was superior to liraglutide in improving memory formation and LTP in the hippocampus [1103], and further in rats treated icv with streptozotocin [1104], a model for sporadic AD [1105,1106]. In agreement with these data, DA4-JC was superior to liraglutide in reducing β−amyloid plaques, reversing memory loss, enhancing synaptic plasticity in the hippocampus, and lowering central pro-inflammatory cytokine levels in APP/PS1/Tau mice [1107]. Improved motor performance is further observed in A53T mice after treatment with DA5-CH, albeit without significance over treatment with liraglutide [1108]. In summary, GIPR agonism improves motor performance and memory while decreasing central inflammation and apoptosis in a variety of rodent models for AD and PD, and these effects are preserved, or even enhanced relative to GLP-1R agonism using GIPR:GLP-1R co-agonists, particularly when using molecules that have been optimized for enhanced BBB permeability [927].

## 19 Summary and outlook

The isolation of GIP and the subsequent recognition of its role as an insulin-releasing incretin hormone generated much interest and research effort. This was nevertheless eclipsed by the advent of molecular biology and the emergence plus subsequent therapeutic exploitation of GLP-1. However, recent years have witnessed a strong resurgence of interest in GIP biology which has not only established GIP as the major incretin hormone but has also uncovered it's many important pleiotropic metabolic effects. Synthesized by intestinal K-cells as pro-GIP and processed by PC1/3, the active form GIP(1-42) is secreted in response to nutrients with possible modulation by neural and hormonal factors. In the blood, it is rapidly inactivated by DPP4, but important physiological effects are mediated rapidly though GIP receptors present on the various target cells. Key actions include enhancing glucose-stimulated insulin secretion, promoting β-cell survival/growth, modulating lipid storage/lipolysis in adipose tissue, reducing body weight through centrally mediated appetite suppression, alleviating drug-induced nausea, lowering peripheral and central inflammation, and promoting both cognition and bone formation. In consequence, changes in the secretion and/or action of GIP have been shown to make important contributions to pathogenesis of obesity-diabetes plus a range of other disorders. GIP insensitivity as encountered in T2DM is not irreversible, so therapeutic enhancement of GIP action through DPP4 inhibition or design of stable GIP analogues is possible. Although not fully understood, both GIP agonism and antagonism surprisingly benefit obesity-diabetes but neither approach is superior to the substantial benefits of recently introduced unimolecular peptides targeting both GIP and GLP-1 receptors. Such agents are now well-established for treating obesity and T2D in humans, and therapies combining GIPR antagonism with GLP-1R agonism have also shown promising results in clinical studies. Although research on GIP continues to generate conflicting views, modifying GIPR signaling has now emerged fully as a strong drug partner in the therapy of various diseases. These include disturbances of energy, glucose and lipid metabolism but benefits may extend in the future to treatment of neurodegenerative, bone and reproductive disorders.

## CRediT authorship contribution statement

**Timo D. Müller:** Writing – original draft, Conceptualization. **Alice Adriaenssens:** Writing – review & editing. **Bo Ahrén:** Writing – review & editing. **Matthias Blüher:** Writing – review & editing. **Andreas L. Birkenfeld:** Writing – review & editing. **Jonathan E. Campbell:** Writing – review & editing. **Matthew P. Coghlan:** Writing – review & editing. **David D'Alessio:** Writing – review & editing. **Carolyn F. Deacon:** Writing – review & editing. **Stefano DelPrato:** Writing – review & editing. **Jonathan D. Douros:** Writing – review & editing, Conceptualization. **Daniel J. Drucker:** Writing – review & editing. **Natalie S. Figueredo Burgos:** Writing – review & editing. **Peter R. Flatt:** Writing – review & editing, Writing – original draft. **Brian Finan:** Writing – review & editing. **Ruth E. Gimeno:** Writing – review & editing. **Fiona M. Gribble:** Writing – review & editing. **Matthew R. Hayes:** Writing – review & editing. **Christian Hölscher:** Writing – review & editing. **Jens J. Holst:** Writing – review & editing. **Patrick J. Knerr:** Writing – review & editing. **Filip K. Knop:** Writing – review & editing. **Christine M. Kusminski:** Writing – review & editing. **Arkadiusz Liskiewicz:** Writing – review & editing. **Guillaume Mabilleau:** Writing – review & editing, Conceptualization. **Stephanie A. Mowery:** Writing – review & editing. **Michael A. Nauck:** Writing – review & editing. **Aaron Novikoff:** Writing – review & editing. **Frank Reimann:** Writing – review & editing. **Anna G. Roberts:** Conceptualization. **Mette M. Rosenkilde:** Writing – review & editing, Writing – original draft. **Ricardo J. Samms:** Writing – review & editing, Conceptualization. **Philip E. Scherer:** Writing – review & editing. **Randy J. Seeley:** Writing – review & editing. **Kyle W. Sloop:** Writing – review & editing. **Christian Wolfrum:** Writing – review & editing, Conceptualization. **Denise Wootten:** Writing – review & editing. **Richard D. DiMarchi:** Writing – review & editing. **Matthias H. Tschöp:** Writing – review & editing.

## Dedication

This review is dedicated to the memory and research of all pioneers who have contributed to the present knowledge of GIP, and in particular the giants John Brown, Viktor Mutt, Ray Pederson, Werner Creutzfeldt and Vincent Marks.

## Declaration of competing interest

DJD has served as a consultant or speaker within the past 12 months to Amgen, AstraZeneca, Boehringer Ingelheim, Kallyope and Novo Nordisk Inc. Neither DJD or his family members hold issued stock directly or indirectly in any of these companies. DJD holds non-exercised options in Kallyope. S.A.M, J.D.D., B.F., and P.J.K. are shareholders and former employees of Novo Nordisk. RJS has received research support from Novo Nordisk, Fractyl, Astra Zeneca, Congruence Therapeutics, Eli Lilly, Bullfrog AI, Glycsend Therapeutics and Amgen. RJS has served as a paid consultant for Novo Nordisk, Eli Lilly, CinRx, Fractyl, Structure Therapeutics, Crinetics, Amgen and Congruence Therapeutics. RJS has equity in Bullfrog AI and Rewind. MMR and JJH are co-founders and shareholders of Antag Therapeutics and Bainan Biotech. MB received honoraria as a consultant and speaker from Amgen, AstraZeneca, Bayer, Boehringer-Ingelheim, Daiichi-Sankyo, Lilly, Novo Nordisk, Novartis, Pfizer and Sanofi. DW is shareholder and on the scientific advisory board of Septerna Inc. and a co-founder and shareholder of Dacra therapeutics. MRH receives research SRA fundining from Boehringer Ingelheim, Eli Lilly & Co., Pfizer, Gila Therapeutics, and Novo Nordisk. MRH is a named inventor of patents pursuant to work that is owned by Syracuse University and the University of Pennsylvania. MRH is a founding scientists and shareholder of Coronation Bio. Inc. FMG and FR have received research support from Eli Lilly and AstraZeneca. FKK has served on scientific advisory panels, been part of speaker's bureaus for, served as a consultant to, owns stocks in and/or received research support from 89bio, Amgen, AstraZeneca, Boehringer Ingelheim, Carmot Therapeutics, Eli Lilly, Gubra, MedImmune, MSD/Merck, Norgine, Novo Nordisk, Sanofi, ShouTi, SNIPR Biome, Zealand Pharma and Zucara. FKK is a co-founder of and minority shareholder in Antag Therapeutics. FKK is currently employed by Novo Nordisk; the present work was done independent of Novo Nordisk. CH is a named inventor on patents that cover GIP or dual GLP-1/GIP receptor agonists as treatments for AD/PD. He is the CSO of Kariya Pharmaceutics Ltd. JJH appears on advisory boards for Novo Nordisk. SDP has served as president of EASD/European Foundation for the Study of Diabetes (EFSD) (2020–2022) and is current president of Fondazione Menarini; has received research grants to the institution from AstraZeneca and Boehringer Ingelheim; has served as advisor for Abbott, Amarin Corporation, Amplitude, Applied Therapeutics, AstraZeneca, Biomea Fusion, Eli Lilly & Co., EvaPharma, Menarini International, Novo Nordisk, Sanofi, and Sun Pharmaceuticals; and has received fees for speaking from AstraZeneca, Boehringer Ingelheim, Eli Lilly & Co., Laboratori Guidotti, Menarini International, Merck Sharpe & Dohme, and Novo Nordisk. PRF has served as consultant for Amgen, Ipsen, Novo Nordisk, Sanofi, Zealand and is co-founder and shareholder of Dia Beta Labs. MHT is a member of the scientific advisory board of ERX Pharmaceuticals, Cambridge, Mass. He was a member of the Research Cluster Advisory Panel (ReCAP) of the Novo Nordisk Foundation between 2017 and 2019. He attended a scientific advisory board meeting of the Novo Nordisk Foundation Center for Basic Metabolic Research, University of Copenhagen, in 2016. He received funding for his research projects by Novo Nordisk (2016–2020) and Sanofi-Aventis (2012–2019). He was a consultant for Bionorica SE (2013–2017), Menarini Ricerche S.p.A. (2016), and Bayer Pharma AG Berlin (2016). As former Director of the Helmholtz Diabetes Center and the Institute for Diabetes and Obesity at Helmholtz Zentrum München (2011–2018), and since 2018, as CEO of Helmholtz Zentrum München, he has been responsible for collaborations with a multitude of companies and institutions, worldwide. In this capacity, he discussed potential projects with and has signed/signs contracts for his institute(s) and for the staff for research funding and/or collaborations with industry and academia, worldwide, including but not limited to pharmaceutical corporations like Boehringer Ingelheim, Eli Lilly, Novo Nordisk, Medigene, Arbormed, BioSyngen, and others. In this role, he was/is further responsible for commercial technology transfer activities of his institute(s), including diabetes related patent portfolios of Helmholtz Zentrum München as, e.g., WO/2016/188932 A2 or WO/2017/194499 A1. MHT confirms that to the best of his knowledge none of the above funding sources were involved in the preparation of this paper. MAN has been member on advisory boards or has consulted with Boehringer Ingelheim, Eli Lilly & Co., Medtronic, Merck, Sharp & Dohme, NovoNordisk, Pfizer, Regor, Sun Pharma, and Structure Therapeutics (ShouTi, Gasherbrum). He has received grant support from Merck, Sharp & Dohme. He has also served on the speakers' bureau of Eli Lilly & Co., Merck, Sharp & Dohme, Medscape, Medical Learning Institute, and NovoNordisk. MAN has been member on advisory boards or has consulted with Boehringer Ingelheim, Eli Lilly & Co., Medtronic, Merck, Sharp & Dohme, NovoNordisk, Pfizer, Regor, Sun Pharma, and Structure Therapeutics (ShouTi, Gasherbrum). He has received grant support from Merck, Sharp & Dohme. He has also served on the speakers' bureau of Eli Lilly & Co., Merck, Sharp & Dohme, Medscape, Medical Learning Institute, and NovoNordisk. MAN has been member on advisory boards or has consulted with Boehringer Ingelheim, Eli Lilly & Co., Medtronic, Merck, Sharp & Dohme, NovoNordisk, Pfizer, Regor, Sun Pharma, and Structure Therapeutics (ShouTi, Gasherbrum). He has received grant support from Merck, Sharp & Dohme. He has also served on the speakers' bureau of Eli Lilly & Co., Merck, Sharp & Dohme, Medscape, Medical Learning Institute, and NovoNordisk. TDM receives funding from Novo Nordisk and has received speaking fees from Novo Nordisk, Eli Lilly, Boehringer Ingelheim, Merck, AstraZeneca, Mercodia and Berlin Chemie AG. TDM further holds stocks from Novo Nordisk and Eli Lilly. BA has served as a speaker within the past 12 months to Mankind and Novartis and is a shareholder of Astra Zeneca, Eli Lilly and Novo Nordisk AS. R.J.S., M.P.C., K.W.S., B.F. and R.E.G. are employees of Eli Lilly and Company and may own company stock.

## Data Availability

No data was used for the research described in the article.
